# Ohm’s Law, the Reconnection Rate, and Energy Conversion in Collisionless Magnetic Reconnection

**DOI:** 10.1007/s11214-025-01142-0

**Published:** 2025-02-10

**Authors:** Yi-Hsin Liu, Michael Hesse, Kevin Genestreti, Rumi Nakamura, James L. Burch, Paul A. Cassak, Naoki Bessho, Jonathan P. Eastwood, Tai Phan, Marc Swisdak, Sergio Toledo-Redondo, Masahiro Hoshino, Cecilia Norgren, Hantao Ji, Takuma K. M. Nakamura

**Affiliations:** 1 Department of Physics and Astronomy, Dartmouth College, Hanover, NH 03750 USA; 2 Ames Research Center, NASA, Moffett Field, CA 94035 USA; 3 Southwest Research Institute, Durham, NH 03824 USA; 4 Space Research Institute, Austrian Academy of Sciences, Schmiedlstraße 6, 8042 Graz, Austria; 5 International Space Science Institute, Bern, Switzerland; 6 Southwest Research Institute, San Antonio, TX 78238 USA; 7 Department of Physics and Astronomy and Center for KINETIC Plasma Physics, West Virginia University, Morgantown, WV 26506 USA; 8 NASA, Goddard Space Flight Center, Greenbelt, MD 20771 USA; 9 Department of Astronomy, University of Maryland, College Park, MD 20742 USA; 10 Department of Physics, Imperial College London, London, UK; 11 Space Science Laboratory, UC Berkeley, Berkeley, CA 94720 USA; 12 IREAP, University of Maryland, College Park, MD 20742 USA; 13 Department of Electromagnetism and Electronics, University of Murcia, Murcia, Spain; 14 Department of Earth and Planetary Science, The University of Tokyo, Tokyo, 113-0033 Japan; 15 Swedish Institute of Space Physics, Uppsala, Sweden; 16 Department of Physics and Technology, University of Bergen, Bergen, Norway; 17 Department of Astrophysical Sciences, Princeton University, Princeton, NJ 08544 USA; 18 Krimgen LLC, Hiroshima, 7320828, Japan

## Abstract

Magnetic reconnection is a ubiquitous plasma process that transforms magnetic energy into particle energy during eruptive events throughout the universe. Reconnection not only converts energy during solar flares and geomagnetic substorms that drive space weather near Earth, but it may also play critical roles in the high energy emissions from the magnetospheres of neutron stars and black holes. In this review article, we focus on collisionless plasmas that are most relevant to reconnection in many space and astrophysical plasmas. Guided by first-principles kinetic simulations and spaceborne in-situ observations, we highlight the most recent progress in understanding this fundamental plasma process. We start by discussing the non-ideal electric field in the generalized Ohm’s law that breaks the frozen-in flux condition in ideal magnetohydrodynamics and allows magnetic reconnection to occur. We point out that this same reconnection electric field also plays an important role in sustaining the current and pressure in the current sheet and then discuss the determination of its magnitude (i.e., the reconnection rate), based on force balance and energy conservation. This approach to determining the reconnection rate is applied to kinetic current sheets with a wide variety of magnetic geometries, parameters, and background conditions. We also briefly review the key diagnostics and modeling of energy conversion around the reconnection diffusion region, seeking insights from recently developed theories. Finally, future prospects and open questions are discussed.

## Introduction

**Fig. 1:**
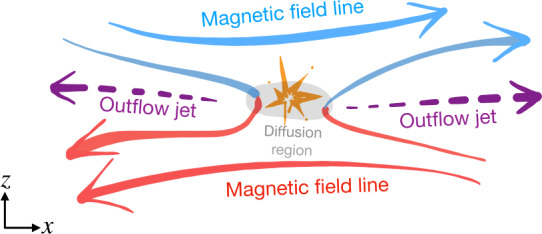
*Artist’s rendition of magnetic reconnection*. The breaking and rejoining of magnetic field lines (red and blue) in the diffusion region (gray) drive plasma outflow jets (purple arrows)

Magnetic reconnection is a ubiquitous process that converts magnetic energy into plasma thermal and kinetic energy in laboratory, space, and astrophysical plasmas (Zweibel and Yamada 2009; Yamada et al. 2010). This efficient energy conversion process involves the effective “breaking” and “rejoining” of magnetic field lines (although note that reconnection does not violate Gauss’ law, $\nabla \cdot {\mathbf{B}}=0$). By altering their connectivity within the so-called “diffusion region” in the microscopic scale, the gray area in Fig. 1, this process imparts energy into outflow plasma jets, the purple arrows. While this picture captures the local process, the resulting change in the magnetic connectivity has far-reaching consequences as it can lead to energy release at large scales in the surrounding plasma systems, causing solar flares (Carmichael 1964; Sturrock 1966; Hirayama 1974; Kopp and Pneuman 1976; Priest and Forbes 2000), planetary geomagnetic substorms (Dungey 1961), and superflares from other astrophysical objects, for example the Crab nebula (Tavani et al. 2011; Abdo et al. 2011; Cerutti et al. 2014).

In a nutshell, magnetic reconnection is a nonlinear, dynamical process that involves electromagnetism, magnetic field geometry and topology, and complex charged particle motions in a multi-dimensional, multiscale system, where physics occurring at a singular point can lead to tremendous energy release at the macroscale. For these reasons, the study of magnetic reconnection has been a fascinating and challenging subject since it was first formulated in 1953 (Dungey 1953). Its study will continue to thrive with our increasing capability to observe electromagnetic phenomena in the universe (e.g., Bale et al. 2023; Burch and Torbert 2016; Raouafi et al. 2023a; Müller et al. 2020). The development of reconnection theories is guided and constrained by a wealth of data from numerical simulations, in-situ and remote space observations, and laboratory experiments. We do not intend to exhaustively include the many great efforts performed in various communities over the past 70 years in this review paper. Here instead we focus on the progress in the past 20 years on collisionless reconnection, where our understanding has been accelerated by kinetic simulations and in-situ spacecraft observations of NASA’s ongoing Magnetospheric Multiscale (MMS) mission (Burch and Torbert 2016), THEMIS/ARTEMIS (Angelopoulos 2008; Sweetser et al. 2011), and Cluster (Escoubet et al. 2001). More exciting results are expected from the Parker Solar Probe (Raouafi et al. 2023a) and Solar Orbiter (Müller et al. 2020) missions, but are not discussed here. It is worth noting that Earth’s magnetosphere and the solar wind are the most ideal testing grounds for reconnection physics reachable by human probes with current space technology. Because the size of a single spacecraft is relatively small compared to the electron kinetic scale, and now the cadence of measurement well resolves the dynamic time scale of reconnection therein; see Genestreti et al. (2025, this collection) for the review on current sheets in geospace. A companion review of collisionless reconnection research in the laboratory over the past 20 years, in comparisons with kinetic simulations and space observations, is given by Ji et al. (2023, this collection).

The fundamental questions of reconnection discussed in this review are: (1) what breaks the ideal-magnetohydrodynamic frozen-in flux condition, enabling reconnection to occur on a microscopic/kinetic scale? And what roles does the non-ideal electric field play (Sect. 2)? (2) what determines the rate at which reconnection processes the incoming magnetic flux (Sect. 3)? and (3) how plasmas are energized around the reconnection diffusion region (Sect. 4)? Each topic can be read independently, and we point out connections between different sections. This article serves as a review but also, hopefully, a tutorial for graduate students and early career scientists.

This review focuses on the fluid-type descriptions of reconnection physics within and around the diffusion region but is based on fully kinetic simulations and in-situ space measurements. It is not our intention to discuss all the details of each topic, but to integrate them into a bigger picture. Nevertheless, references that contain the full treatment are provided to interested readers. The discussion of the rich kinetic features and particle distribution functions is beyond the scope of this paper but can be found in Norgren et al. (2025, this collection). For discussions of a broader scope or emphasis on other areas of study, a variety of other papers complement this review (Vasyliunas 1975; Priest and Forbes 2000; Birn and Priest 2007; Zweibel and Yamada 2009; Mozer and Pritchett 2010; Yamada et al. 2010; Gonzalez and Parker 2016; Burch and Torbert 2016; Lee and Lee 2020; Ji et al. 2022; Pontin and Priest 2022; Yamada 2022).

## Breaking the Frozen-in Flux Condition

Alfvén’s frozen-in flux theorem (Alfvén 1942) shows that perfectly conducting fluids, such as those in ideal magnetohydrodynamics (MHD), and embedded magnetic fields are constrained to move together. Mathematically, this occurs when ${\mathbf{E}} +{\mathbf{V}} \times {\mathbf{B}}/c$ vanishes (e.g., Stern 1966; Newcomb 1958).^1^ In a hypothetical plasma for which the frozen-in flux theorem is satisfied, the total magnetic flux going through any closed Ampèrian loop in the plasma does not change in time. Note that the magnetic field self-consistently evolves with the moving plasma, which can generate currents that modify the magnetic fields. If the frozen-in condition works everywhere within the system of interest, the connectivity of magnetic field lines within this system cannot change because doing so would change the flux through a closed loop somewhere within the system.

The field line connectivity, nevertheless, can change when some dissipation breaks the frozen-in condition. For instance, the condition breaks down within the diffusion region (DR) in Fig. 1 that is sandwiched by magnetic field lines that point in opposite directions. Within this diffusion region, the inflowing magnetic field lines are “rewired” to form highly curved (blue-red) field lines, which are again frozen to the plasma outside the diffusion region and act like a slingshot, shooting plasma out as outflow jets. Once the plasma is jetted out, the plasma pressure within the diffusion region drops, and plasma flows in from the top and bottom along with the magnetic field for further reconnection. It is thus a self-driven (i.e., spontaneous) non-linear process; once it starts, it does not want to stop as long as more magnetic field is available in the inflow region. In addition, because of Ampère’s law, the anti-parallel fields sandwich a current sheet where the DR resides. The singular point inside the DR where field lines reconnect is referred to as the “X-point” because the adjacent reconnected field lines form an X-shape. In 3D, the collection of these X-points extends in the out-of-plane direction to form an “X-line”.

### The Generalized Ohm’s Law

Magnetic reconnection is the process that changes the field line connectivity in plasmas, and it requires the existence of a reconnection electric field to break the frozen-in flux condition, either at a topological boundary (Vasyliunas 1975), or, more generally, in a localized region parallel to the magnetic field (Hesse and Schindler 1988; Hesse et al. 2005). This requirement is a simple consequence of Maxwell’s equations and the need to transport magnetic flux from the inflow to the outflow regions. While it has long been shown that reconnection cannot proceed without the presence of such a reconnection electric field, we only recently are understanding its full physical foundations.

Beginning with Vasyliunas (1975), it was recognized that the reconnection electric field has to be balanced by one or more terms in the generalized Ohm’s law (Vasyliunas 1975; Cai and Lee 1997; Hesse et al. 2011). Writing the electron momentum equation in collisionless plasmas and solving for ${\mathbf{E}}$ gives,

1 $$ {\mathbf{E}}+\frac{{\mathbf{V}}_{e} \times{\mathbf{B}}}{c}=- \frac{\nabla \cdot {\mathbf{P}}_{e}}{ne}-\frac{m_{e}}{e}({\mathbf{V}}_{e}\cdot \nabla ){\mathbf{V}}_{e}-\frac{m_{e}}{e}\frac{\partial}{\partial t}{\mathbf{V}}_{e}, $$

where variables ${\mathbf{E}}$, ${\mathbf{B}}$, ${\mathbf{V}}_{e}$, ${\mathbf{P}}_{e}$, $n$, $e$, $m_{e}$ and $c$ are electric field, magnetic field, electron velocity, electron pressure tensor, number density, proton charge, electron mass and the speed of light, respectively, and Gaussian units are used.

Since $m_{e}$ is small, the last two terms are only appreciable if the electron speed $V_{e}$ is much larger than the ion speed $V_{i}$, so in those terms we can replace ${\mathbf{V}}_{e}\simeq -{\mathbf{J}}/ne$, where ${\mathbf{J}}$ is the current density. Then, we obtain the generalized Ohm’s Law close to that discussed in Vasyliunas (1975),

2 $$ {\mathbf{E}}+\frac{{\mathbf{V}}_{i} \times{\mathbf{B}}}{c}= \frac{{\mathbf{J}}\times{\mathbf{B}}}{nec}-\frac{\nabla \cdot {\mathbf{P}}_{e}}{ne}- \frac{m_{e}}{e^{2}}\left (\frac{\mathbf{J}}{ne}\right )\cdot \nabla \left ( \frac{{\mathbf{J}}}{n}\right )+\frac{m_{e}}{e^{2}} \frac{\partial}{\partial t}\left (\frac{{\mathbf{J}}}{n}\right ). $$

The left-hand side (LHS) of Eq. (2) measures the ion frozen-in condition, which is violated when its value is non-zero. Terms on the right-hand side (RHS) contribute to this violation. In collisionless plasmas, it includes, from left to right, the Hall electric field [$({\mathbf{J}}\times {\mathbf{B}})/nec$], the divergence of electron pressure term, the spatial derivative of the electron inertia term, and the temporal derivative of the electron inertia term. With collisions, one also needs to include the resistive electric field $\eta {\mathbf{J}}$, but it is omitted from our treatment.

**Fig. 2:**
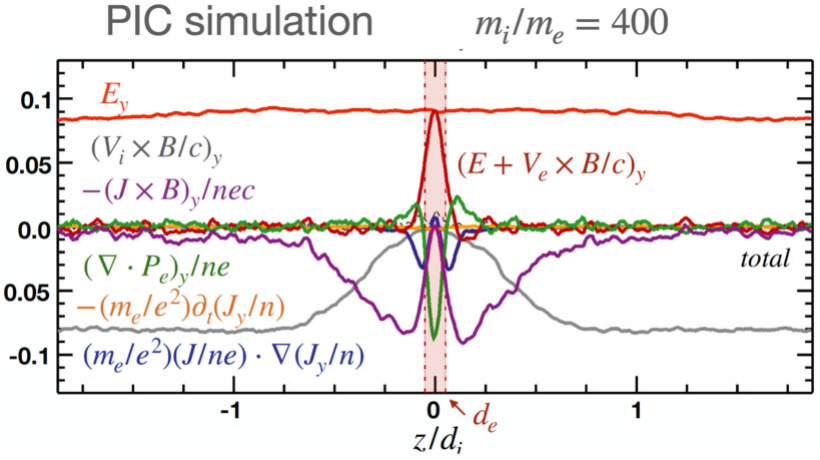
*The generalized Ohm’s law* in symmetric, antiparallel low-$\beta $ reconnection. The out-of-plane component of terms in the generalized Ohm’s law (normalized by $B_{x0}V_{A0}/c$) across the X-line in the inflow ($z$) direction, based on a particle-in-cell simulation of reconnection. The vertical red transparent band marks the electron diffusion region (EDR), while the ion diffusion region (IDR) expands between $z\in [{-d_{i},d_{i}}]$. Adapted from Liu et al. (2022), reproduced by permission of Springer Nature

Here we consider a symmetric, anti-parallel low-$\beta $ reconnection in a Particle-in-Cell (PIC) simulation. Figure 2 shows the out-of-plane ($y$) component of the terms in the generalized Ohm’s law (Eq. (2)) in a cut through the X-line in the inflow ($z$) direction. Upstream of the ion diffusion region (IDR) at $\vert z \vert >d_{i}$ (the ion inertial scale $d_{i}\equiv c/\omega _{pi}$, where $\omega _{pi}=\sqrt{m_{i}/(4\pi n_{i} e^{2})}$ is the ion plasma frequency), ion convection brings magnetic field in, inducing the motional electric field (in gray). The Hall electric field (in purple) becomes the dominant term supporting the reconnection electric field $E_{y}$ (in red) between the $d_{i}$ and the electron inertial scale ($d_{e}\equiv c/\omega _{pe}$, where $\omega _{pe}=\sqrt{m_{e}/(4\pi n_{e} e^{2})}$ is the electron plasma frequency). The Hall term arises because of the decoupling of the relatively immobile ions from the motion of electrons that remain frozen to the magnetic field (Sonnerup 1979), which becomes significant beneath the ion inertial ($d_{i}$)-scale. The divergence of the electron pressure tensor is important within the electron gyro-scale because the off-diagonal component of a species’ pressure tensor becomes pronounced only when the gradient scale of the magnetic field is small or comparable to particles’ thermal gyro-radius ($\rho _{e}=m_{e} cv_{the}/e B$) or bounce lengths (Hesse et al. 2011). The spatial derivative of electron inertia is important within the electron inertial scale. The maximum of the gyro-scale and $d_{e}$ determines the scale of the electron diffusion region (EDR). The time-derivative electron inertial term is negligible in the steady-state shown here, but it is significant in the initiation stage of reconnection, or in the presence of very fast fluctuations with time scales on the order of the electron plasma period (Vasyliunas 1975).

By inspection of Eq. (2), we see that the Hall term vanishes at the X-line, and so does the spatial-derivative inertia term in the symmetric case where the flow stagnation point (${\mathbf{V}}_{e,xz}=0$) coincides with the X-line. In addition, $\partial /\partial t=0$ in the steady state. These leave us with the divergence of the electron pressure tensor, $(\nabla \cdot {\mathbf{P}}_{e})_{y}=\partial _{x} P_{exy}+\partial _{z} P_{ezy}$, which, at a quasi-2D reconnection X-line (i.e., $\partial /\partial y=0$), needs to have off-diagonal pressure components in order to balance the reconnection electric field. These off-diagonal terms around the X-line arise from the non-gyrotropic feature of the electron distributions. Hence, it has been proposed that the electron pressure tensor term should be the main contributor to the reconnection electric field at the reconnection site, at least in 2D symmetric situations (Vasyliunas 1975; Dungey 1988; Lyons and Pridmore-Brown 1990; Cai and Lee 1997; Hesse et al. 1999). The physical origin of the existence of a non-gyrotropic pressure tensor can be traced back to the free acceleration of electrons by the reconnection electric field but only within the unmagnetized EDR (Kulsrud et al. 2005; Hesse et al. 2011).

In an asymmetric configuration (discussed further in Sect. 3.2), the situation is slightly different in that the inertial term in Eq. (2) does not necessarily vanish at the X point. Instead, it is possible that the inertial term contributes part of, or even the majority of the reconnection electric field at this location (Hesse et al. 2014). However, we see from Eq. (2) that non-gyrotropic pressure tensor effects still need to exist at the flow stagnation point (Hesse et al. 2014), which is typically shifted toward the inflow region with a stronger magnetic field (Cassak and Shay 2007, 2008). A simple analysis shows that non-gyrotropic pressure effects are not only expected at the flow stagnation point, but are essential for consistent magnetic flux transport (Hesse et al. 2014). Recent research has further indicated that the reconnection electric field is a consequence of the need to maintain the current density in the electron diffusion region, which would otherwise be reduced by non-gyrotropic electron pressure effects (Hesse et al. 2018). These authors also showed that the thermal interaction of accelerated particles with the adjacent magnetic field, which gives rise to non-gyrotropic pressures and quasi-viscous current reductions, simultaneously leads to electron heating. This electron heating appears to be the key contributor to maintaining pressure balance in the electron diffusion region (see Sect. 2.3 for more discussion).

### Observational Analysis of the Generalized Ohm’s Law

Determining which non-ideal terms are responsible for violating the frozen-in flux condition in EDRs was one of the major objectives of NASA’s Magnetospheric Multiscale (MMS) mission (Burch et al. 2016a). Note that in the decades preceding MMS observations from many previous satellite missions had confirmed the predominance of the Hall term in the IDR (Nagai et al. 2001; Øieroset et al. 2001; Mozer et al. 2002; Eastwood et al. 2010). The four identical MMS spacecraft are each capable of measuring the three-dimensional electromagnetic field vector (Torbert et al. 2016b) and electron and ion velocity space distribution functions (Pollock et al. 2016) at very high time resolution. The spacecraft orbits are typically maintained such that the fleet flies in a tightly-spaced tetrahedral formation with inter-spacecraft separations that can be on the order of the electron inertial length (Fuselier et al. 2016). During crossings through an EDR, differences in the electron and ion fluid moments are obtained between spacecraft pairs, such that, for the first time, the gradient terms in Eq. (2) can be approximated (Chanteur 1998). For more information on the methods, readers are directed to Hasegawa et al. (2024, this collection) and Paschmann and Daly (1998).

**Fig. 3:**
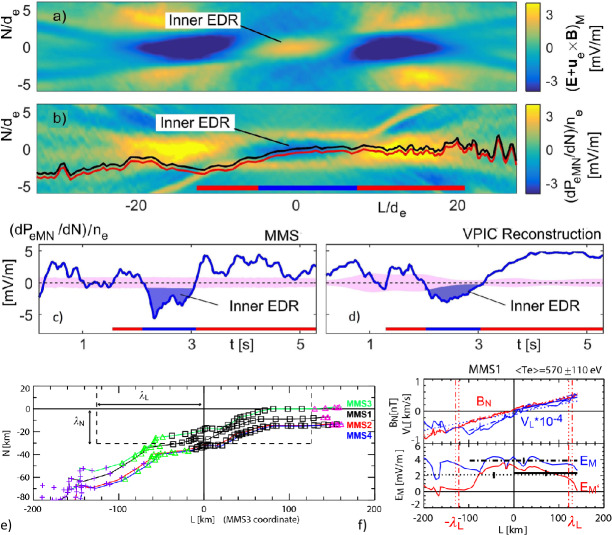
*Non-gyrotropic pressure gradient in the inner EDR* Simulated 2D profiles of (a) $({\mathbf{E}} + {\mathbf{V}}_{e} \times {\mathbf{B}}/c)_{M}$ and (b) $\partial P_{eMN}/\partial N$. Based on the MMS data, the 1D profile of $\partial P_{eMN}/\partial N$ in (c) was obtained, representing a close match to the simulated profile along the MMS trajectory in (d). Adapted from Egedal et al. (2019). (e) Four MMS spacecraft orbit relative to the inner diffusion region (bounded by dashed lines). (f) $B_{N}$ and $V_{eL}$ (top panel) and profile of $E_{M}$ and $E'_{M}$ around the X-line. The three horizontal lines (i.e., dash-dotted, solid, and dotted) in the bottom panel show the electric field value estimated from Hesse et al. (1999) formula using $\partial _{L}V_{eL}$ obtained from data points between different intervals indicated by the length of these lines. Adapted from Nakamura et al. (2019)

MMS has confirmed that the divergence of the electron pressure tensor dominates other non-ideal terms in EDRs near reconnection X-lines. This finding is consistent with the fact that most reconnection events observed by MMS have small or negligible electron flows in the reconnection plane at the X-line when measured in the co-moving frame of the X-line. Egedal et al. (2019) analyzed a symmetric and nearly-anti-parallel EDR observed by MMS on 11 July 2017, evaluated the electron pressure gradient using MMS data, and compared it with a 2D PIC simulation that used initial conditions based on the observations. Egedal et al. (2019) found that the non-gyrotropic pressure components $\partial _{x}P_{exy}+\partial _{z}P_{ezy}$ were predominantly responsible for balancing the reconnection electric field $E_{y}$, especially the latter term. Figure 3(a) and (b) show the numerical profile of $({\mathbf{E}} + {\mathbf{V}}_{e} \times {\mathbf{B}}/c)_{M}$ and the pressure gradient $\partial P_{eMN}/\partial N$ with the projected trajectory of the spacecraft, determined by matching the observed magnetic field data to the simulated profile of the current sheet (see more detail in Egedal et al. 2019). Note that the $LMN$ coordinate system is often used for reconnection observations once the quasi-2D reconnection plane is determined. It corresponds to the $XYZ$ coordinate system shown in Fig. 1, used in most theoretical discussions of this review. The inner EDR is marked with a blue color in Fig. 3(c). The profile of $\partial P_{eMN}/\partial N$ is in excellent agreement with both theory and the simulation, being the main contribution to the non-ideal electric field within the inner electron diffusion region (EDR) where the electron frozen-in condition is broken.

An alternative method to calculate the non-gyrotropic pressure term at the inner EDR is to use the theory by Hesse et al. (1999, 2011), where the spatial scale of the electron diffusion is given by the electron orbit excursion in a field reversal, the so-called “bounce widths”, ${\lambda}_{L}$ and ${\lambda}_{N}$ in the $L$ and $N$ directions respectively, and expressed as ${\lambda}_{L,N} = [2m_{e}T_{e}/(e^{2}(\partial _{L,N}B_{N,L})^{2})]^{1/4}$. The electric field in the electron diffusion region, i.e., the non-gyrotropic pressure term, can then be expressed as $E_{\mathrm{M},\mathrm{model}}\simeq (1/e)(\partial _{L}V_{eL})(2m_{e}T_{e})^{1/2}$. This result was confirmed by Nakamura et al. (2019) based on MMS analysis of the same event by determining the spacecraft orbit relative to an X-line as shown in Fig. 3(e). As expected for the inner EDR, the observed $E_{\mathrm{M}}$ and $E_{\mathrm{M'}}=({\mathbf{E}} + {\mathbf{V}}_{e} \times {\mathbf{B}}/c)_{M}$ in Fig. 3(f) coincide when the spacecraft was inside the inner diffusion region (bounded by the dashed lines in Fig. 3(e)). The dash-dotted horizontal line is the $E_{\mathrm{M},\mathrm{model}}$ calculated from the velocity gradients obtained from the upper panel of Fig. 3(d). The model shows a good agreement with the observed electric field in the inner EDR, indicating that the theoretical concept of the inner EDR for laminar reconnection presented by Hesse et al. (1999, 2011) is well recovered for this event. This means that the divergence of the non-gyrotopic pressure term obtained with this model is also consistent with the reconnection electric field. The same scheme used by Hesse et al. (1999, 2011) to determine the off-diagonal pressure gradient has been also applied to an EDR event during more turbulent magnetotail (symmetric) reconnection and also obtained good agreement with the observed electric field (Ergun et al. 2022), indicating that the Ohm’s law for laminar reconnection can be maintained even in a turbulent environment.

**Fig. 4:**
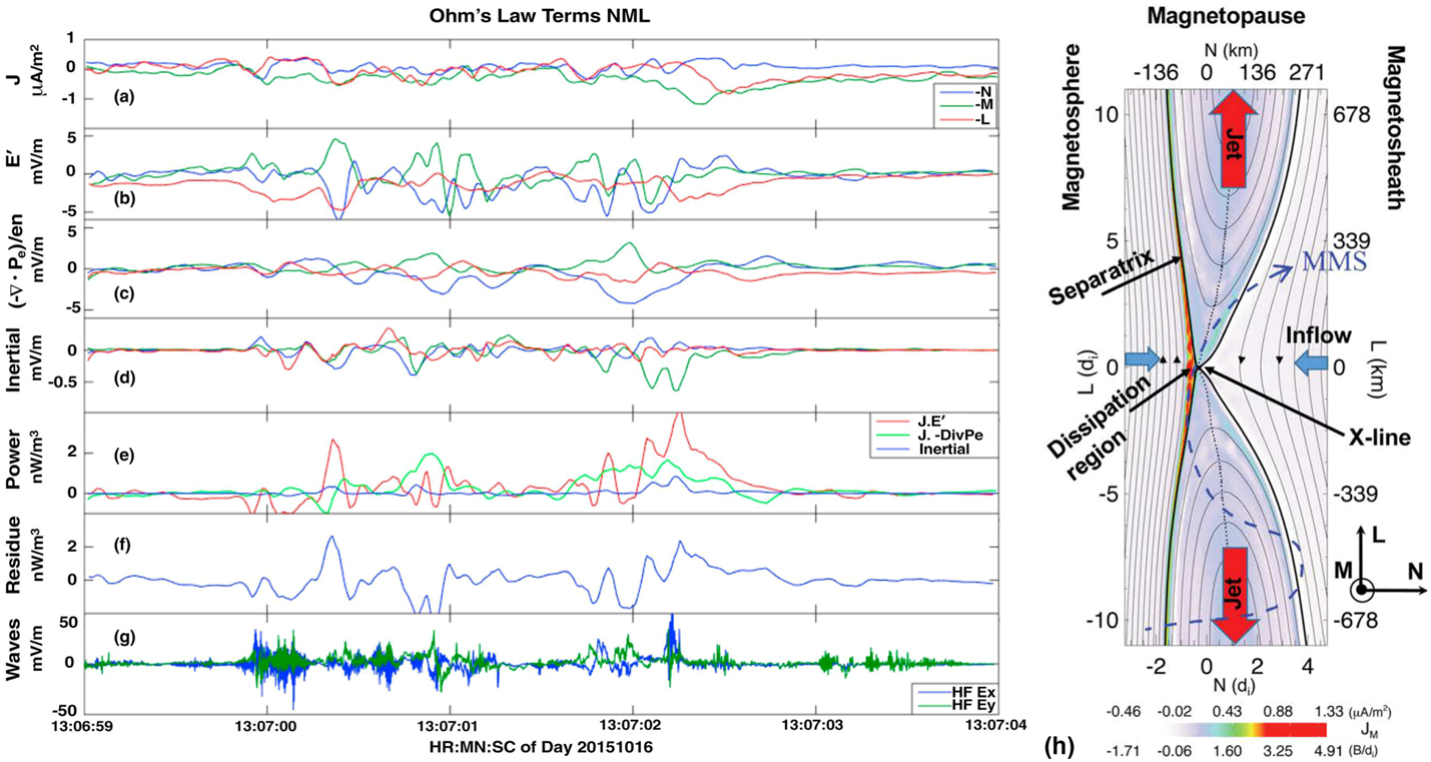
*The generalized Ohm’s law for an asymmetric reconnection event observed by MMS* (a) Three components of the magnetopause current density. (b–d) The comparison of terms in Ohm’s law for interval around 13:07:02 UT when the MMS fleet traversed an EDR (e) the total power dissipation from individual terms. (f) The residue, ${\mathbf{J}}\cdot \{{\mathbf{E'}}-(-\nabla \cdot{\mathbf{P}}_{e}/{en_{e}})-{m_{e}} \nabla \cdot{[n_{e}}({\mathbf{V}}_{i}{\mathbf{V}}_{i}-{\mathbf{V}}_{e}{\mathbf{V}}_{e})]/e{n_{e}} \}$, (g) The full wave amplitude at frequencies up to 4 kHz, (h) The schematic of MMS path through EDR. Adapted from Torbert et al. (2016a) and Burch et al. (2016b)

During anti-parallel asymmetric reconnection, the non-ideal electric field is balanced by a combination of the electron inertial and pressure terms, as described at the end of Sect. 2.1 and confirmed by MMS observations, as shown in Fig. 4. MMS encountered the EDR at around 13:07:02 UT at a negative $J_{M}$ peak. It is seen that the contributions of the inertial term (Fig. 4d) are generally smaller than the pressure term (Fig. 4c), but at times can be comparable, in particular for the M (green) component only, which is primarily along the reconnection electric field. Overall both electron pressure gradients and electron inertial effects are important, with a ratio of about 4:1. Yet, there are residuals of a few mV/m (30-50% of the $\mathbf{E'}$) during the encounters with the electron stagnation point (Fig. 4f) and it was also found that the error in the gradient approximation was considerable (Torbert et al. 2016a). Rager et al. (2018) analyzed the same event with higher time resolution (7.5 ms) electron data and concluded that Ohm’s law could not be fully accurately resolved even with the 7.5 ms data due to time variability on the scale of the energy sweep of the particle instrument and smoothing of spatial structures by the four spacecraft gradient operator. One possibility of the violation of the Generalized Ohm’s law has been suggested to be evidence of anomalous resistivity (Torbert et al. 2016a). Yet, the results of the kinetic simulation performed for the event (Torbert et al. 2016a) suggested that its effect is not significant. The small contribution from the anomalous resistivity to the Generalized Ohm’s law is supported also by Graham et al. (2022) based on direct estimation of the anomalous resistivity, viscosity, and cross-field electron diffusion (see Eq. (45) in Sect. 3.10.1) associated with lower hybrid waves during another asymmetric reconnection event measured by MMS. It was shown that the anomalous resistivity is approximately balanced by anomalous viscosity. Hence, although waves do produce an anomalous electron drift and diffusion across the current layer associated with magnetic reconnection, their contribution to the reconnection electric field is considered to be negligible during this observation. More discussions of Ohm’s law during the presence of 3D fluctuations will be deferred to Sect. 3.10.1.

The dominant role of the pressure divergence over the inertial term in Ohm’s law has also been seen for cases of guide field reconnection in both a symmetric (Wilder et al. 2017) and asymmetric (Genestreti et al. 2018c) current sheet. Genestreti et al. (2018c) also found that both out-of-the-reconnection-plane gradients $\partial _{M}$ and in-plane $\partial _{L,N}$ in the pressure tensor contribute to energy conversion near the X-point. A finite $\partial _{M} P_{eMM}\simeq \partial _{\|}P_{e,\|}$ near the X-line was also observed in 3D guide field reconnection simulations that have a significant 3D structure (Liu et al. 2013; Stanier et al. 2019).

### The Nature of the Reconnection Electric Field

A question of more than just academic nature is how there is a reconnection electric field at all. This question transcends the simple conclusion from above that there has to be a reconnection electric field for flux to be transferred from inflow to outflow. This existence question was raised by Hesse et al. (2018), who investigated the current and energy balance in the electron diffusion region.

During the initial phase of an evolving symmetric reconnecting current sheet, the time-derivative of the electron inertia term, $(m_{e}/e)\partial {\mathbf{V}}_{e}/\partial t$ in Eq. (1), is the only non-ideal term available to break the frozen-in condition at the X-line. This dominance causes the continuous intensification of the current density (${\mathbf{J}}\simeq -en {\mathbf{V}}_{e}$) at the X-line, leading to a sharp current density peak around the electron gyro-scale $\rho _{e}$, generating a non-gyrotropic particle distribution (Hesse et al. 2011; Aunai et al. 2013; Zenitani and Nagai 2016) that eventually makes $\nabla \cdot {\mathbf{P}}_{e}$ the dominant non-ideal term in the quasi-steady ($\partial _{t}=0$) phase; note again that $\nabla \cdot {\mathbf{P}}_{e}$ is the only term available to support the reconnection electric field at the X-line in the steady state of a symmetric case. This transition of the dominant non-ideal term during this current density intensification is clearly demonstrated in PIC simulations (Liu et al. 2014b).

Focusing on the quasi-steady state, Hesse et al. (2018) investigated the electron momentum equation Eq. (1), rewritten in the form of a current evolution equation:

3 $$ \frac{\partial J_{ey}}{\partial t} =\frac{e^{2}n_{e}}{m_{e}}E_{y}+ \frac{e^{2}n_{e}}{m_{e}c} ({\mathbf{V}}_{e}\times{\mathbf{B}})_{y}+ \frac{e}{m_{e}}\left (\frac{\partial P_{eyz}}{\partial z}+ \frac{\partial P_{exy}}{\partial x}\right )-\nabla \cdot ({\mathbf{V}}_{e} J_{ey}). $$

The terms on the right-hand side (RHS) of this equation describe, in order, the electric field force (due to the reconnection electric field $E_{y}$), conversion of in-plane to out-of-plane current by Lorentz forces, pressure gradient forces, and current convection into or out of the volume of interest.

**Fig. 5:**
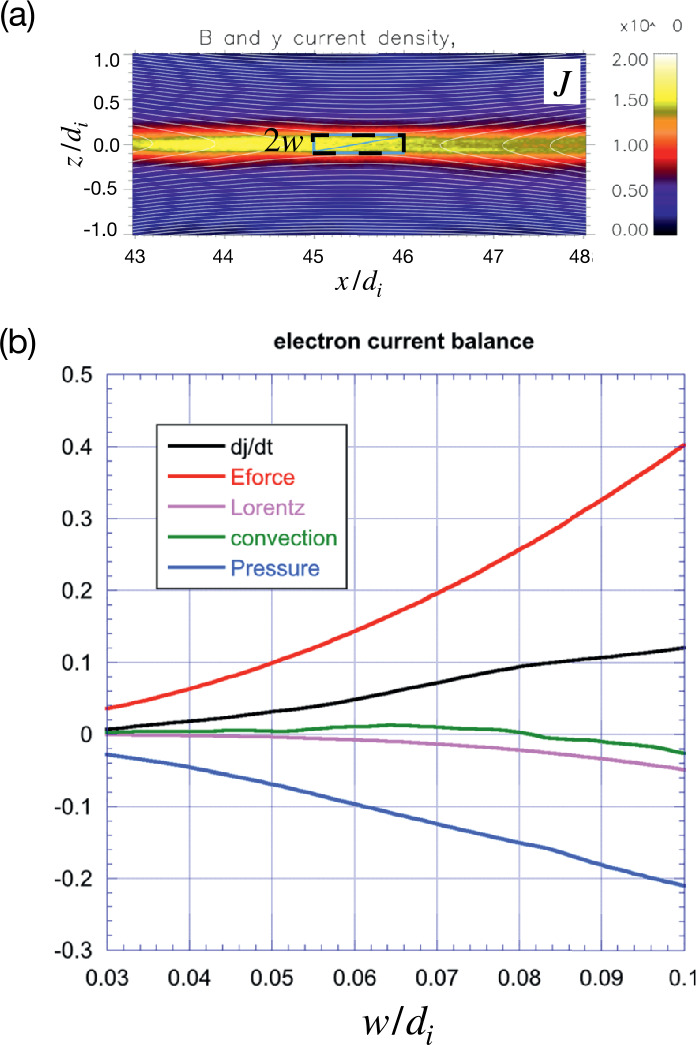
*Terms that balance the current density in the quasi-steady state*. (a) The largest integration region centered on the X-point is shown as the blue-black rectangle with a thickness of 0.2 $d_{i}$ and a lateral extent of one $d_{i}$. The box size varies from an initially much smaller size while keeping the aspect ratio fixed. (b) Integration of the electron current balance equation as a function of the integration region half-thickness “w”. The integrated time derivative of the out-of-place current density is indicated by the black curve, which is also equal to the sum of the four other curves in agreement with Eq. (3). The major contributors are the current increase by the reconnection electric field (red), and the current reduction by pressure effects (blue). The dominance of these two terms holds over the entire range rather than at the X-point alone. Adapted from Hesse et al. (2018)

To evaluate the balance of these terms over a larger domain that contains the singular X-line in the steady state, Hesse et al. (2018) integrated the individual terms of this equation over rectangles of different sizes, centered about the X-line location in a PIC simulation of symmetric magnetic reconnection, as shown in Fig. 5(a). The results of this integration are displayed in Fig. 5(b). The only terms of importance are the non-gyrotropic pressure terms (i.e., $\partial P_{eyz}/\partial z+\partial P_{exy}/\partial x$), which act to reduce the current density, and the electric field term, which acts to increase it. These terms roughly balance each other, keeping the electron current density constant, or varying on very slow time scales to account for the overall system evolution.

Hesse et al. (2018) further found that the source of the electron pressure, $P_{e}\equiv{\mathrm{Tr}}({\mathbf{P}}_{e})/3$, within the EDR is essentially exclusively due to the non-gyrotropic effect, i.e., due to complex particle orbits rather than simple, gyrotropic behavior. For this purpose, the equation

4 $$ \frac{\partial P_{e}}{\partial t}=-\nabla \cdot ({\mathbf{V}}_{e}P_{e})- \frac{2}{3}\sum _{l} P_{e,ll}\frac{\partial V_{el}}{\partial x_{l}}- \frac{1}{3}\sum _{l,i}\frac{\partial Q_{e,lii}}{\partial x_{i}}- \frac{2}{3}\sum _{l,i\neq l}P_{e,li} \frac{\partial V_{el}}{\partial x_{i}} $$

was integrated over the varying rectangle, as before, in a way similar to Fig. 5(b). The result (not shown) was that among the terms on the RHS of Eq. (4), the last term provided a positive contribution, whereas negative contributions were provided by the first two terms. The heat flux term $Q_{e,lii}$ is negligible. The dominant non-gyrotropic pressure $P_{e,li}$ contributions here appeared to be the same as the ones acting to reduce the current density in Eq. (3), suggesting that the conversion of the current carrier motion to the plasma pressure plays an important role. This last term plus the second term on the RHS of Eq. (4) is basically the “pressure-strain interaction” (Yang et al. 2017b,a), $-({\mathbf{P}}\cdot \nabla )\cdot {\mathbf{V}}$, that will be further discussed in Sect. 4.3).

**Fig. 6:**
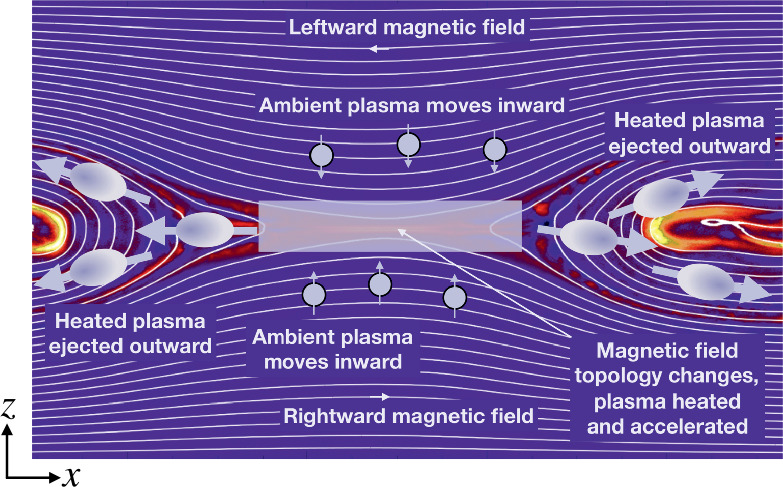
*The nature of the reconnection electric field*. Shown are in-plane magnetic field lines (white), and the out-of-plane current density contour. In addition to breaking the frozen-in condition and transporting the flux into and out of the diffusion region, the reconnection electric field also sustains the electric current and increases the thermal pressure through the $-({\mathbf{P}}\cdot \nabla )\cdot {\mathbf{V}}$ term within the diffusion region. Reprinted from Hesse and Cassak (2020), with the permission of Wiley

In short, the reconnection electric field within the diffusion region converts incoming electromagnetic energy into the current carrier bulk kinetic energy through direct acceleration. The current at the electron gyro-scale is intensified until the current density gradient is strong enough to generate complex, non-gyrotropic particle distribution (i.e., non-zero $P_{eyz}$ and $P_{exy}$), which funnels the current carrier kinetic energy into the thermal energy through the $-({\mathbf{P}}\cdot \nabla )\cdot {\mathbf{V}}$ term (Eq. (4)). This makes the steady state possible, where $\partial _{t} J_{ey}$ vanishes and the $\nabla \cdot {\mathbf{P}}$ becomes strong enough to balance the reconnection electric field in the Ohm’s law (Eq. (3) or Eq. (2)). This multifaceted nature of the reconnection electric field is highlighted in Fig. 6.

An alternative viewpoint is also offered in Hesse et al. (2018), which argues that the electric field exists as a consequence of Maxwell’s equations, specifically, Ampère’s law:

5 $$ \frac{\partial {\mathbf{E}}}{\partial t}=c\nabla \times {\mathbf{B}}-4\pi {\mathbf{J}}. $$

Imagine that the current density is reduced by “a mechanism” below what is required to balance the $\nabla \times {\mathbf{B}}$ term. Then Ampère’s law, Eq. (5), will immediately signal the need to increase the electric field, which accelerates the current carriers and re-establishes balance in the steady state.

In other words, the steady-state reconnection electric field exists because there is a mechanism at work, which attempts to “dissipate” the current density. This conclusion holds irrespective of the dissipation mechanism – for example, classical collisions would have the same effect, as captured in Ohm’s law ${\mathbf{J}}=\sigma {\mathbf{E}}$ where $\sigma $ is the conductivity determined by the collisions. In a collisionless plasma, however, the current dissipation is provided by non-gyrotropic pressure effects, which are a manifestation of complex particle orbits, which lead to the scattering of directed motion by the local magnetic geometry. Hesse et al. (2021) extended this investigation to asymmetric systems (defined in Sect. 3.2) and found that the overall conclusions also hold there, even though some of the current reduction was found to be due to convective effects, in addition to the above-discussed non-gyrotropic pressure effects.

## Collisionless Magnetic Reconnection Rate

In this section, we discuss how one can determine the magnitude of the reconnection electric field $E_{R}$ (i.e., the $E_{y}$ at the X-line), which is essentially the reconnection rate that measures how fast reconnection processes the incoming magnetic flux. We will organize the discussions of different regimes using the governing force-balance equation, as it determines the characteristic reconnection outflow speed, being critical to the rate. To avoid a common confusion in the normalization of reconnection rates, we normalize $E_{R}$ by the “asymptotic” value of reconnecting magnetic field component $B_{R}$ (or $B_{x0}$) and the associated proton Alfvén speed in the upstream region $V_{A}\equiv B_{R}/\sqrt{4\pi n m_{i}}$ to define the “normalized reconnection rate” $R\equiv cE_{R}/B_{R}V_{A}$. While we have aimed to unify the notation throughout this review, some differences in subsections are unavoidable in order to strike the balance between simplicity and consistency. New notations, if needed, are defined with respect to the coordinates shown in the relevant figures.

### Standard Symmetric Anti-Parallel Reconnection

**Fig. 7:**
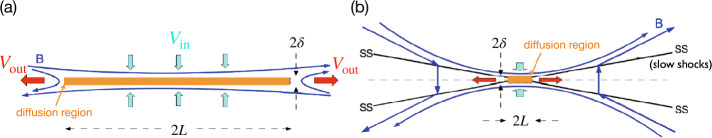
*Classical reconnection models*. (a) Sweet-Parker solution. Adapted from Sweet (1958), Parker (1957). (b) Petschek solution. Adapted from Petschek (1964)

We begin with the simplest current sheet, one that has symmetric, antiparallel magnetic fields, as illustrated in Fig. 7. Combining the electron and ion momentum equations, we can derive the MHD force balance equation. In the steady state, it reads

6 $$ \frac{({\mathbf{B}}\cdot \nabla ){\mathbf{B}}}{4\pi}\simeq \frac{\nabla B^{2}}{8\pi}+\nabla \cdot {\mathbf{P}}+nm_{i}({\mathbf{V}}\cdot \nabla ){\mathbf{V}}. $$

This force-balance equation works in most regions, including the ideal MHD region and the ion diffusion region, as long as the electron inertial term is negligible and the quasi-neutral condition holds; that is usually valid in the non-relativistic limit. As we will see, the scaling of reconnection rates in diverse regimes can be more or less captured by the force balance along the inflow and outflow symmetry lines.

#### Sweet-Parker Scaling

The first quantitative model of the magnetic reconnection rate was derived by Sweet and Parker (Parker 1957; Sweet 1958). From mass conservation $\nabla \cdot (n{\mathbf{V}})\simeq 0$ in steady state and the incompressible assumption,

7 $$ V_{\mathrm{in}}L \simeq V_{\mathrm{out}}\delta , $$

where $\delta $ and $L$ are the half-thickness and half-length of the diffusion region, respectively. From the momentum equation, balancing the magnetic tension and inertia force in Eq. (6), $({\mathbf{B}}\cdot \nabla ) {\mathbf{B}}/4\pi \simeq n m_{i} ({\mathbf{V}}\cdot \nabla ){\mathbf{V}}$, one gets

8 $$ V_{\mathrm{out}}\simeq \frac{B_{R}}{\sqrt{4\pi n m_{i}}}=V_{A}. $$

Thus, the outflow speed is the characteristic Alfvén speed based on the upstream magnetic field $B_{R}$. It then makes sense to define the *normalized reconnection rate* as

9 $$ R\equiv \frac{V_{\mathrm{in}}}{V_{A}}, $$

which measures how fast the inflowing plasma transports magnetic flux for processing. Combining Eqs. (7) and (8), one realizes that the normalized reconnection rate is basically the aspect ratio of the diffusion region, i.e., $R\simeq \delta /L$. Note that $E_{y}$ is uniform in the 2D steady-state per Faraday’s law and $E_{y}=V_{\mathrm{in}}B_{R}/c$ at the inflow boundary of the diffusion region, thus the definition of the reconnection rate can also be expressed as $R\equiv cE_{R}/(B_{R} V_{A})$.

After coupling the inflow region to a diffusion region dominated by resistivity (which requires collisions), the full Sweet-Parker solution (omitted here) was derived in 1957. It has a system size long current sheet (Fig. 7(a)), resulting in a low $\delta /L$ and thus a reconnection rate that is too low to explain the energy release during solar flares (Parker 1963, 1973). Petschek (1964) proposed that standing slow shocks that bound the exhaust can resolve this challenge by opening out the outflow geometry, and localizing the diffusion region (Fig. 7(b)). However, Petschek reconnection, unlike Sweet-Parker reconnection, is not a solution of resistive-MHD with uniform resistivity; it tends to collapse into the long Sweet-Parker layer in (uniform) resistive-MHD simulations (Sato and Hayashi 1979; Biskamp 1986). Such an elongated reconnection layer can be unstable to the plasmoid instability if collisions are weak enough (Biskamp 1982; Shibata and Tanuma 2001; Bhattacharjee et al. 2009; Loureiro et al. 2007; Pucci and Velli 2014; Comisso et al. 2016), but we will not discuss this resistive-MHD mode further.

#### $R-S_{\mathrm{lope}}$ Relation and the Maximum Plausible Rate

For collisionless reconnection, the rate in Eq. (9) is not bounded, since $\delta /L$ can, in principle, be any value. To fix this problem, one needs to consider force balance along the inflow direction and recognize there is a scale separation between the regions “immediately upstream” and “far (asymptotic) upstream” of the ion diffusion region.

**Fig. 8:**
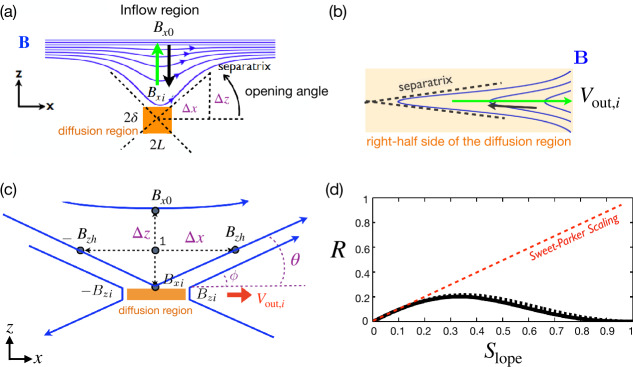
*General constraints on the maximum plausible rate from mesoscale force-balance.* (a) Field line geometry and force balance upstream of the diffusion region. (b) Field line geometry and force balance along the outflow. (c) The scheme used to analyze the force balance. (d) The predicted reconnection rate $R$ as a function of the separatrix slope $S_{\mathrm{lope}}$. Adapted from Liu et al. (2017)

Per geometry, this diffusion region aspect ratio $\delta /L$ is also the slope of the separatrix $S_{\mathrm{lope}}=\Delta z/\Delta x$, as shown in Fig. 8(a). The $R\simeq \delta /L$ scaling only works when $S_{\mathrm{lope}}=\delta /L\ll 1$. In the $S_{\mathrm{lope}}\rightarrow 1$ limit (i.e., a localized diffusion region with an open outflow geometry as illustrated in Fig. 8(a)), the upstream magnetic field is indented, which unavoidably induces a magnetic tension force $({\mathbf{B}}\cdot \nabla ){\mathbf{B}}/4\pi $ pointing to the upstream region, as illustrated by the green arrow in Fig. 8(a). In the low-$\beta $ limit, both the upstream $\nabla \cdot {\mathbf{P}}$ and $nm_{i}({\mathbf{V}}\cdot \nabla ){\mathbf{V}}$ terms are negligible; thus, the only term that can counterbalance this tension force is the magnetic pressure gradient force ($-\nabla B^{2}/8\pi $, black arrow), which requires the reduction of the reconnecting field when it is convected into the diffusion region. Similarly, a finite magnetic pressure gradient force also arises in the outflow direction in the $S_{\mathrm{lope}}\rightarrow 1$ limit, as depicted by the black arrow in Fig. 8(b), which slows down the outflow.

Quantitatively, through discretizing the inflow force-balance at point ${\mathbf{1}}$ of Fig. 8(c), one can relate the ratio of the magnetic field immediately upstream of the ion diffusion region $B_{xi}$ and the asymptotic value at far upstream $B_{x0}$ to the slope of the reconnection separatrix $S_{\mathrm{lope}}$ (Liu et al. 2017) as

10 $$ \frac{B_{xi}}{B_{x0}}\simeq \frac{1-S_{{\mathrm{lope}}}^{2}}{1+S_{{\mathrm{lope}}}^{2}}. $$

In the large opening limit ($S_{\mathrm{lope}}\rightarrow 1$), the magnetic field $B_{xi}$ that actually reconnects is reduced, as is the rate $R$.

By analyzing the outflow force balance, including the $nm_{i}({\mathbf{V}}\cdot \nabla ){\mathbf{V}}$ term at a point within the diffusion region (Fig. 8(b)), one can derive the outflow speed at the outflow edge of the ion diffusion region,

11 $$ V_{{\mathrm{out}},i}\simeq V_{Ai}\sqrt{1-S_{{\mathrm{lope}}}^{2}}, $$

where $V_{Ai}\equiv B_{xi}/\sqrt{4\pi nm_{i}}$. Equation (11), coupled with Eq. (10), recovers the Alfvén speed $V_{A0}=B_{x0}/\sqrt{4\pi n m_{i}}$ in the small opening limit, as in the Sweet-Parker analysis. In the large opening ($S_{\mathrm{lope}} \rightarrow 1$) limit, the outflow speed is reduced, and so is the rate.

The reconnection rate is $R=cE_{y}/B_{x0}V_{A0}=(B_{zi}/B_{xi})(B_{xi}/B_{x0})(V_{{ \mathrm{out}},i}/V_{A0})$. Using Eqs. (10) and (11), and noting that $B_{zi}/B_{xi}\simeq S_{\mathrm{lope}}$, we then find the $R-S_{\mathrm{lope}}$ relation

12 $$ R=S_{{\mathrm{lope}}}\left ( \frac{1-S_{{\mathrm{lope}}}^{2}}{1+S_{{\mathrm{lope}}}^{2}}\right )^{2}\sqrt{1-S_{{ \mathrm{lope}}}^{2}}, $$

which is shown as the black solid curve in Fig. 8(d). Clearly, these two geometrical constraints along the inflow and outflow bring down the reconnection rate to zero in the $S_{\mathrm{lope}}\rightarrow 1$ limit, where the separatrix makes a right angle. The maximum plausible rate is around the value of 0.2. For reference, the Sweet-Parker scaling is shown by the red dashed line, which is unbounded in the large $S_{\mathrm{lope}}$ limit. Importantly, the profile of the predicted black curve is relatively flat for a wide range of $S_{\mathrm{lope}}$. Thus as long as there is some degree of localization, the predicted rate will be on the order of $\mathcal{O}(0.1)$. Note that this prediction does not depend on the dissipation physics or the thickness of the current sheet. Thus, this value likely also constrains the maximal plausible rate in theorized “turbulent reconnection” where the diffusion region is turbulent and thick, and has large-scale outflow exhausts (Lazarian and Vishniac 1999). On the other hand, this maximum plausible reconnection rate of value $\simeq 0.2$ is clearly demonstrated using a large and spatially localized anomalous resistivity right at the X-line in MHD simulations (Lin et al. 2021; Jiménez et al. 2022).

#### Localization Mechanism That Leads to Fast Reconnection

Petschek (1964) provided the correct steady-state outflow solution of reconnection that predicts slow shocks and rotational discontinuities farther downstream, but the solution failed to capture the essential *localization mechanism*, that leads to the open geometry in the first place. While Eq. (12) provides the general $R-S_{\mathrm{lope}}$ relation, to determine the rate, we still need to identify the (primary) mechanism that localizes the diffusion region, determining the opening geometry captured by $S_{\mathrm{lope}}$.

**Fig. 9:**
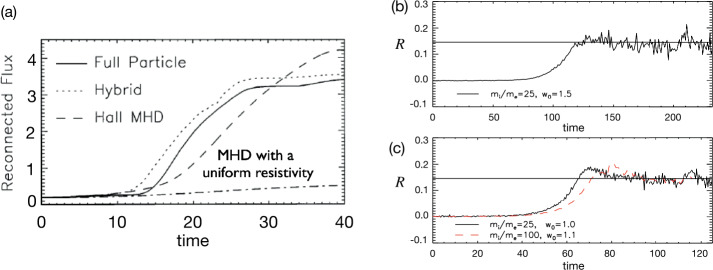
(a) The GEM reconnection challenge result that compares reconnection rates in four different numerical models. Reprinted from Birn et al. (2001), with the permission of Wiley. Panels (b) and (c) show the normalized reconnection rate in PIC simulations over a range of mass ratio $m_{i}/m_{e}$ and initial thickness $w_{0}$. Adapted from Shay et al. (2007)

Kinetic simulations beyond the MHD model suggest that antiparallel reconnection with an open outflow geometry occurs when the current sheet thins down to the ion inertial scale (Bhattacharjee 2004; Cassak et al. 2005; Daughton et al. 2009; Jara-Almonte and Ji 2021). When this occurs, the Hall term in the generalized Ohm’s law (Vasyliunas 1975; Swisdak et al. 2008) dominates the electric field in the ion diffusion region (IDR), where the ions become demagnetized. The correlation between the Hall effect and fast reconnection was clearly demonstrated in the GEM reconnection challenge study (Birn et al. 2001), as shown in Fig. 9(a). This study showed that simulation models with the Hall term in the generalized Ohm’s law (particle-in-cell (PIC), hybrid, and Hall-MHD) realize fast reconnection, while only the uniform resistive-MHD model, which lacks the Hall term, exhibits a slow rate (Parker 1957; Sweet 1958). The value of the fast rate in collisionless plasmas is on the order of 0.1 over a range of electron-ion mass ratios and initial thicknesses, as shown in Fig. 9(b) and (c). For decades, it had been unclear “how” the Hall term localizes the diffusion region, producing an open geometry. The dispersive property of waves arising from the Hall term was proposed as an explanation (Mandt et al. 1994; Shay et al. 1999; Rogers et al. 2001; Drake et al. 2008), but the role of dispersive waves derived from the linear analysis was called into question because reconnection can be fast even in systems that lack dispersive waves (Bessho and Bhattacharjee 2005; Liu et al. 2014b; TenBarge et al. 2014; Stanier et al. 2015a).

**Fig. 10:**
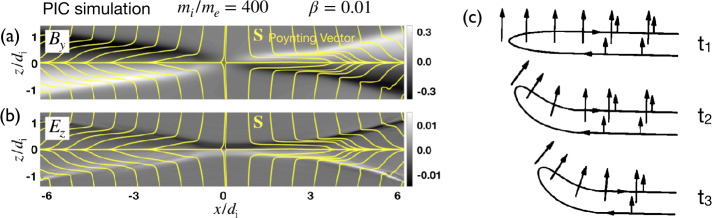
*Hall electromagnetic fields* (a) The Hall magnetic field $B_{y}$ and (b) the Hall electric field $E_{z}$ (normalized by $B_{x0}$) overlaid with Poynting vector ${\mathbf{S}}$ streamlines (yellow). Adapted from Liu et al. (2022), reproduced by permission of Springer Nature. (c) Electrons drag the reconnected field out of the reconnection plane to form the Hall quadrupole magnetic field. Adapted from Mandt et al. (1994)

While the Hall electromagnetic fields were well-known for being the key feature of the ion diffusion region (Sonnerup 1979), their role in transporting the incoming magnetic energy was less recognized. Figure 10a shows the out-of-plane magnetic field $B_{y}$ in the non-linear stage. This out-of-plane quadrupolar Hall magnetic field arises because electrons, the primary current carrier within the IDR (i.e., ${\mathbf{J}} \simeq -e n {\mathbf{V}}_{e}$), drag both reconnected and not-yet reconnected magnetic field lines out of the reconnection plane (Mandt et al. 1994; Ren et al. 2005; Drake et al. 2008; Burch et al. 2016b), as illustrated in Fig. 10(c); the laboratory evidence is reviewed in Ji et al. (2023, this collection). Importantly, this Hall quadrupole magnetic field $B_{y}$ along with the inward-pointing Hall electric field $E_{z}\simeq V_{ey} B_{x}/c$, shown in Fig. 10b, constitute a Poynting vector $S_{x}=-cE_{z} B_{y}/4\pi $ in the $x$-direction. This component diverts the inflowing electromagnetic energy toward the outflow direction. This is shown by the streamlines of ${\mathbf{S}}=c{\mathbf{E}}\times{\mathbf{B}}/4\pi $ in yellow, which bend in the $x$ direction significantly before reaching the outflow symmetry line at $z=0$.

**Fig. 11:**
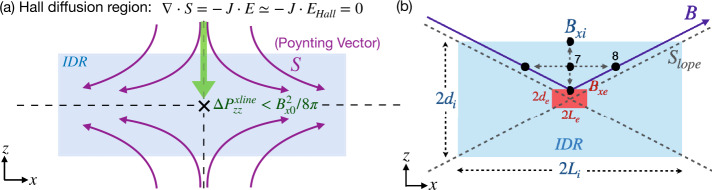
*Transport patterns of electromagnetic energy in Hall reconnection and the diffusion region structure.* (a) The Hall effect results in this intrinsic, “diverting”, Poynting vector ${\mathbf{S}}$ streamline pattern in purple, which limits the pressure increase of inflowing plasma (green arrow) that reaches the X-line. (b) The diagram used to derive the slope of the separatrix, $S_{{\mathrm{lope}}}$. The blue (red) box represents the IDR (EDR). The solid blue line depicts an upstream magnetic field $\mathbf{B}$ line adjacent to the separatrix shown by diagonal dashed lines. Adapted from Liu et al. (2022), reproduced by permission of Springer Nature

Since the Hall term dominates the electric field ${\mathbf{E}}\simeq {\mathbf{E}}_{{\mathrm{Hall}}} = {\mathbf{J}}\times {\mathbf{B}}/nec$ inside the IDR, then $\nabla \cdot {\mathbf{S}} = -{\mathbf{J}}\cdot {\mathbf{E}} \simeq 0$ per Poynting’s theorem in the steady state (n.b., further discussion of Poynting’s theorem can be found in Sect. 4.2). When the divergence of a vector field vanishes, like that for magnetic fields (i.e., $\nabla \cdot{\mathbf{B}}=0$), Gauss’ theorem indicates that the associated flux into a closed volume equals the flux out. The associated flux of ${\mathbf{S}}$ can be quantified by the number of streamlines equally spaced at the inflow boundary. These ${\mathbf{S}}$ streamlines (purple lines in Fig. 11(a)) into the IDR (blue region) do not end within the region of $\nabla \cdot {\mathbf{S}}=0$. Approaching the X-line, the magnetic field strength decreases and eventually vanishes due to the symmetry of this system. The ${\mathbf{S}}$ streamlines thus need to get around this singular point and exit at the outflow direction. This results in an intrinsically “diverting” ${\mathbf{S}}$ streamline pattern around the X-line, consistent with the presence of $S_{x} = -cE_{z}B_{y}/4\pi $, as discussed in Fig. 10(a) and (b). These streamlines play a role analogous to railroad tracks in guiding the transport of incoming (magnetic) energy through the IDR. From Fig. 11(a), we realize that if ${\mathbf{E}}={\mathbf{E}}_{\mathrm{Hall}}$ none of the upstream magnetic energy can be transported to the X-line due to this diverting ${\mathbf{S}}$ streamline pattern.

As time proceeds, an energy void centered around the X-line develops. Without energy input, no pressure (either thermal or magnetic) can be built up at the X-line. The upstream magnetic pressure around the energy void will then locally pinch the upstream magnetic field lines. This is a localization mechanism needed for the open outflow geometry and fast reconnection (Liu et al. 2022). Here we point out three more important observations. First, the diverted energy is deposited on the outflow symmetry line (i.e., $z=0$) downstream of the X-line, which helps establish the pressure balance across the exhaust (in the normal direction), keeping the exhaust open. This difference of energy content at the X-line and its downstream region itself also implies the localization of the diffusion region. Second, this diverting **S** streamline pattern persists even in an (initially) elongated reconnection layer, but such a layer is not sustainable, as noted above. Third, in resistive-MHD, $\nabla \cdot {\mathbf{S}} = -{\mathbf{J}}\cdot{\mathbf{E}} \simeq -\eta J_{y}^{2} < 0$ since the resistivity $\eta $ is always positive. Thus, diverting ${\mathbf{S}}$ streamlines are not required (i.e., $S_{x}\simeq 0$ is possible). The $\mathbf{S}$ streamlines can end and distribute energy uniformly on the outflow symmetry line, in favor of maintaining the pressure balance across the X-line. This is why the diffusion region in Sweet-Parker reconnection is not localized.

To quantify the degree of localization, we need to estimate the thermal pressure at the X-line. The key is that ${\mathbf{J}}\cdot{\mathbf{E}}\simeq 0$ inside the Hall-dominated IDR, which limits the energy conversion to particles and thus also limits the difference in the $zz$-component of the pressure tensor between the X-line and the far upstream asymptotic region $\Delta P_{zz}^{\mathrm{xline}} \equiv P_{zz}\vert _{\mathrm{xline}}- P_{0}$ (illustrated as the green arrow in Fig. 11a). Given that magnetic pressure $B^{2}/8\pi =0$ at the antiparallel reconnection X-line, as long as $\Delta P_{zz}^{\mathrm{xline}} < B_{x0}^{2}/8\pi $, the inflowing reconnecting field bends toward the X-line to restore the force-balance condition $\nabla (P+B^{2}/8\pi )=({\mathbf{B}}\cdot \nabla ){\mathbf{B}}/4\pi $ (Liu et al. 2020a). This bending makes the outflow exhausts open out.

These observations can be used to determine the separatrix slope $S_{\mathrm{lope}}$, then using the $R-S_{\mathrm{lope}}$ relation discussed in the previous Sect. 3.1.2; it will provide a first-principles prediction of reconnection rate in collisionless plasmas. Specifically, after recognizing that some limited energy still goes to the ballistically accelerated incoming ions (Wygant et al. 2005; Aunai et al. 2011), one can derive the pressure difference between the $d_{e}$- and $d_{i}$-scale (Liu et al. 2022),

13 $$ P_{izz}\vert _{d_{i}}^{d_{e}} \simeq \frac{2}{3}\left ( \frac{B_{xi}^{2}-B_{xe}^{2}}{8\pi}\right ), $$

where $B_{xe}$ is the reconnecting magnetic field at the inflow boundary of the EDR. and the slope of the separatrix associated with the open outflow geometry can then be determined by analyzing the inflow force balance at point ${\mathbf{7}}$ of Fig. 11(b),

14 $$ S_{{\mathrm{lope}}}\simeq \sqrt{\frac{1}{3}\left [ \frac{1-(B_{xe}/B_{xi})}{1+(B_{xe}/B_{xi})}\right ]}, $$

where

15 $$ \frac{B_{xe}}{B_{xi}} \simeq \left (\frac{m_{e}}{m_{i}}\right )^{1/4} $$

is derived through coupling to the EDR. The cross-scale coupling from the mesoscale upstream region down to the IDR, and then the EDR is achieved by recognizing that the magnetic field line tends to straighten itself out (when it is possible). Thus, the separatrix slope $S_{\mathrm{lope}}$ is similar in these different regions. For the real proton-to-electron mass ratio $m_{i}/m_{e}=1836$, the total pressure increase along the inflow symmetry line to the X-line was derived to be $\Delta P^{xline}_{zz}\simeq 0.25 B_{x0}^{2}/8\pi $, and the resulting reconnection rate $R\simeq 0.16$ from Eq. (12), consistent with numerical simulations in Fig. 9, in-situ observations (Genestreti et al. 2018b; Nakamura et al. 2018; Torbert et al. 2018; Nakamura et al. 2019) discussed in Sect. 3.4.1, and other examples discussed in a previous review (Cassak et al. 2017b).

### Asymmetric Reconnection

While “symmetric” magnetic reconnection discussed in the previous subsection (Sect. 3.1) is a reasonable approximation to the energy release process during geomagnetic substorms at Earth’s magnetotail (e.g. Angelopoulos et al. 2008; Paschmann et al. 2013), magnetic reconnection at Earth’s magnetopause is “asymmetric”, as it occurs at the boundary layer between the magnetosphere plasmas and magnetosheath plasmas (e.g. Paschmann et al. 2013, 2005, 1979), where the plasma and magnetic field conditions on two sides of the current sheet can be very different. In this subsection, we will generalize the theoretical modeling into this configuration.

#### Cassak-Shay Scaling

To predict the rate of asymmetric reconnection in terms of upstream plasma parameters, we make the same simplifying assumptions typically made for such studies: two-dimensionality, steady state, upstream asymptotic magnetic fields are straight and anti-parallel, no bulk flow upstream except for the inflow, and the upstream plasmas are in local thermodynamic equilibrium. The analysis is carried out in the reference frame in which the X-line is stationary. We use subscripts “1” and “2” to denote the two upstream sides of the reconnection site, and the upstream reconnecting magnetic field strengths are $B$, number densities are $n$, and temperatures are $T$. For definiteness, if the magnetic field strength is stronger on one side than the other, we take the stronger magnetic field side to be “2”, so that $B_{2} \geq B_{1}$.

First, we note that there must be a pressure balance in the MHD sense across the current sheet in the upstream asymptotic regions

16 $$ P_{1} + \frac{B_{1}^{2}}{8 \pi} = P_{2} + \frac{B_{2}^{2}}{8 \pi}. $$

Here the total plasma pressure is $P= \sum _{s}^{i,e} n k_{B} T_{s}$. In writing Eq. (16), we ignore the ram pressure due to the inflow speed $V_{\mathrm{in}}$. This is justifiable *a posteriori* because the resulting normalized reconnection rate $R$ is $\mathcal{O}(0.1)$ and the ratio of the inflow kinetic energy density $(1/2) nm_{i} V_{\mathrm{in}}^{2}$ to the upstream magnetic pressure $B^{2}/8 \pi $ scales like $R^{2}$, so the contribution of the ram pressure due to the inflow is at the $1\%$ level. Pressure balance follows from the momentum equation; if pressure balance were not satisfied, the current sheet would have a net force on it and would accelerate, which would violate the assumption that the system is in a steady state.

**Fig. 12:**
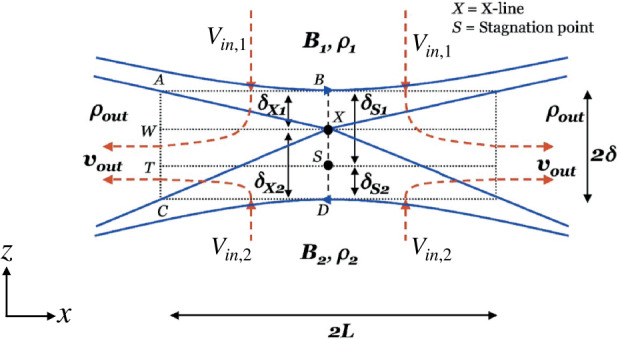
*Sketch of asymmetric reconnection diffusion region.* Magnetic field lines (blue solid lines) and bulk flow streamlines (red dashed lines) in asymmetric reconnection. The outer gray rectangle denotes the edge of the diffusion region. $X$ denotes the location of the X-line, and $S$ denotes the location of the stagnation point, where the in-plane magnetic field and bulk flow go to zero, respectively. Reprinted from Cassak and Shay (2007), with the permission of AIP Publishing

The most basic estimate of the asymmetric reconnection rate in terms of upstream parameters is obtained using a generalization of the classical Sweet-Parker analysis (Cassak and Shay 2007). This approach simply relies on conservation laws, which impose that the flux of particles, energy, and magnetic flux coming in the upstream edge of the diffusion region must equal their fluxes leaving at the downstream edge in the steady state. The diffusion region is assumed to be a rectangular box of half-thickness $\delta $ in the inflow direction and half-length $L$ in the outflow direction, as sketched in Fig. 12. We first treat the limit in which the process is incompressible (Cassak and Shay 2007).

In the steady-state in two dimensions, Faraday’s law implies that the out-of-plane electric field $E_{y}$ must be uniform. At the upstream edge of the (ion) diffusion region, the ideal-MHD Ohm’s law is expected to be valid, so ${\mathbf{E}} + {\mathbf{V}} \times {\mathbf{B}} / c \simeq 0$. An important result follows; defining the inflow speeds as $V_{{\mathrm{in}},1}$ and $V_{{\mathrm{in}},2}$, the constancy of $E_{y}$ implies

17 $$ V_{{\mathrm{in}},1} B_{x1} \sim V_{{\mathrm{in}},2} B_{x2}. $$

Since we assume $B_{x2} \geq B_{x1}$, this result implies the stronger magnetic field convects into the diffusion region more slowly. By conservation of particles, the flux of particles entering the diffusion region must equal its flux as it leaves, which is quantified as

18 $$ (n_{1} V_{{\mathrm{in}},1} + n_{2} V_{{\mathrm{in}},2}) L \sim 2 n_{{\mathrm{out}}} V_{{ \mathrm{out}}} \delta , $$

where $n_{{\mathrm{out}}}$ and $V_{{\mathrm{out}}}$ are the number density and (outflow) bulk speed at the downstream edge of the diffusion region. Similarly, the conservation of energy implies

19 $$ \left (V_{{\mathrm{in}},1} \frac{B_{x1}^{2}}{8\pi} + V_{{\mathrm{in}},2} \frac{B_{x2}^{2}}{8\pi}\right ) L \sim 2 \left (\frac{1}{2} n_{{\mathrm{out}}}m_{i} V_{{\mathrm{out}}}^{2}\right ) V_{{\mathrm{out}}} \delta . $$

Finally, it was argued that the outflow number density scales as

20 $$ n_{{\mathrm{out}}} \sim \frac{n_{1} B_{x2} + n_{2} B_{x1}}{B_{x1} + B_{x2}}, $$

which follows from the plasmas mixing in proportion to the volume of the flux tubes on either upstream side, since the weaker magnetic field side reconnects more volume than the stronger magnetic field. Putting the results of Eqs. (17) through (20) together give predictions for the outflow speed $V_{\mathrm{out}}$ and the reconnection electric field $E_{R,asym}$:

21 $$\begin{aligned} V_{{\mathrm{out}}} \simeq V_{A,{\mathrm{asym}}} \sim & \sqrt{ \frac{B_{x1} B_{x2}}{4 \pi n_{{\mathrm{out}}}m_{i}}}, \end{aligned}$$

22 $$\begin{aligned} E_{R,{\mathrm{asym}}} \sim & 2\left ( \frac{B_{x1} B_{x2}}{B_{x1} + B_{x2}}\right ) \left (\frac{\delta}{L} \right ) \frac{V_{{\mathrm{out}}}}{c}. \end{aligned}$$

This gives the desired asymmetric reconnection rate as a function of upstream parameters. For each expression, the result reduces to the standard incompressible Sweet-Parker scaling $V_{{\mathrm{out}}} \sim V_{A}$ and $E_{R} \sim (\delta /L) V_{A} B_{R} / c$ in the symmetric limit.

The analysis described thus far did not take compressibility into account, which allows for the heating of the plasma as it passes through the diffusion region. The analysis was extended (Birn et al. 2010) to include these effects. The way to do so involves replacing magnetic energy $B^{2}/8\pi $ with magnetic enthalpy $B^{2}/4\pi $ and including the enthalpy flux $[\gamma /(\gamma -1)] P \equiv \kappa P$ (where $\gamma $ is the ratio of specific heats in the fluid description) in the energy flux balance in Eq. (19). The predicted outflow speed ends up being unchanged from Eq. (21), but the predicted reconnection rate is multiplied by a factor of $r$ given by

23 $$ r = \frac{\kappa (B_{x1} + B_{x2})}{\lambda _{1} B_{x2} + \lambda _{2} B_{x1}}, $$

where $\lambda _{j} = (1 + \kappa \beta _{j})/(1 + \beta _{j})$, and plasma $\beta _{j} = 8 \pi P_{j} / B_{xj}^{2}$ for $j = 1, 2$. Taking the incompressible limit with either $\beta _{j} \rightarrow \infty $ or $\gamma \rightarrow \infty $ reproduces Eq. (22). A similar scaling analysis was extended to relativistic asymmetric magnetic reconnection (Mbarek et al. 2022).

#### $R-S_{\mathrm{lope}}$ Relation and the Maximum Plausible Rate

Both results from the previous section predict the reconnection rate in terms of asymptotic upstream parameters but have a factor of $\delta / L$. It can be calculated for resistive reconnection analogously to the Sweet-Parker model (Cassak and Shay 2007), but for collisionless reconnection $\delta /L$ remained as a free parameter. Empirically from two-fluid simulations, it was found that $\delta / L \sim 0.1$ (Cassak and Shay 2008) for collisionless asymmetric reconnection, just like it does for symmetric reconnection (Shay et al. 1999). However, it is important to better understand why this is the case. The analysis for symmetric reconnection used to show that 0.1 is approximately the maximum reconnection rate allowed (Liu et al. 2017) was extended to asymmetric reconnection (Liu et al. 2018a).

**Fig. 13:**
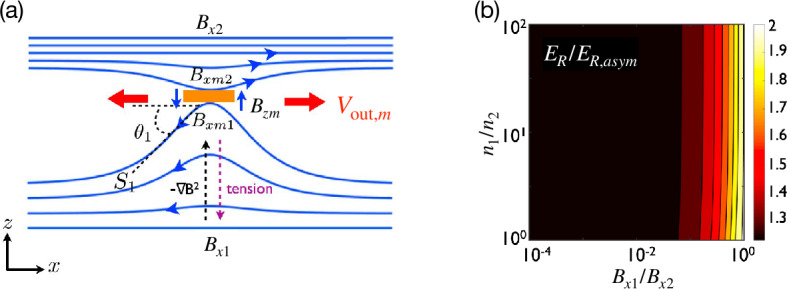
*Estimation of the maximum plausible reconnection rate in asymmetric reconnection.* (a) The magnetic field geometry of asymmetric reconnection, where the field strength is different on two sides of the diffusion region (orange box). (b) The predicted ratio of the maximum reconnection electric field $E_{R}$ and $E_{R,asym}$ in Eq. (22) (with an effective $\delta /L=0.1$) over a wide range of magnetic field and density asymmetries is within a factor of 2. Reprinted from Liu et al. (2018a), with the permission of Wiley

In this model, the reconnecting magnetic fields at the mesoscale bend in towards the reconnection site as they do in symmetric reconnection. Force balance in the inflow direction is analogous to the symmetric reconnection case, where the magnetic curvature force opposes the magnetic pressure force between the X-line and the asymptotic region, reducing the magnetic field at the microscopic scale, as illustrated in Fig. 13(a). For asymmetric reconnection, however, the geometry on the two sides of the current sheet is different, specifically the slopes of the separatrix on the two sides of the current sheet. Analogous to Eq. (10) for the magnetic field strength at the upstream edge of the diffusion region in symmetric reconnection, one gets

24 $$ \frac{B_{xmj}}{B_{xj}}\simeq \frac{1-S_{{\mathrm{lope}},j}^{2}}{1+S_{{\mathrm{lope}},j}^{2}}, $$

for the two sides $j=1,2$ and the subscript “$m$” denotes the edges of the “microscopic” ion diffusion region. Here $S_{{\mathrm{lope}},j} = \delta _{j} / L$ is the slope made by the separatrix within the diffusion region, which can be different on each side and $\delta _{1}+\delta _{2}=\delta $. The reconnected magnetic field $B_{zm}$ at the downstream edge is assumed to be the same for each side.

A brief analysis predicts the outflow speed when taking into account the reduction of the upstream magnetic field and magnetic pressure, giving

25 $$ V_{{\mathrm{out}},m} \simeq \sqrt{ \frac{B_{xm1}B_{xm2}}{4\pi n_{{\mathrm{out}},m}m_{i}}} \sqrt{1-4 \frac{B_{xm1}B_{xm2}}{(B_{xm1}+B_{xm2})^{2}}\left (\frac{\delta}{L} \right )^{2}}, $$

which generalizes Eq. (21) and captures that the outflow speed decreases when the exhausts open out. The subscript “$m$” again indicates quantities at the edges of the ion diffusion region. With Eqs. (24) and (25), an expression for the normalized reconnection rate $R$ can be obtained that is only a function of $S_{{\mathrm{lope}},1}$ (or $S_{{\mathrm{lope}},2}$) and the upstream plasma parameters, as was done for symmetric reconnection in Fig. 8(d). The prediction of the maximum rate shows a similar scaling with magnetic field ratio and density ratio as Eq. (22), as shown in Fig. 13(b). Importantly, this analysis reveals that it is the reduction of the reconnecting magnetic field on the weak field side (i.e., $B_{x1}$ in Fig. 13) that limits the reconnection rate. The slopes of separatrix on two sides are also predicted (Liu et al. 2018a). As of now, there has not been a first-principles calculation of the reconnection rate for collisionless asymmetric reconnection, generalizing the symmetric result in Sect. 3.1.3.

#### Structure of the Diffusion Region During Asymmetric Reconnection

In addition to the asymmetric conditions modifying the macroscale properties of the reconnection, such as the outflow speed and reconnection rate, they also impact the microscale physics within the diffusion region. One key result is that the X-line (the location at which the magnetic topology changes) and the stagnation point (the location at which the in-plane bulk flow goes to zero) are not in the same location (Hoshino and Nishida 1983; Scholer 1989; La Belle-Hamer et al. 1995; Nakamura and Scholer 2000; Priest et al. 2000; Dorelli et al. 2004; Mirnov et al. 2006; Cassak and Shay 2007). The reason follows from conservation laws (Cassak and Shay 2007). From Eq. (17), the inflow is slower on the high magnetic field side. Counter-intuitively, the rate at which the magnetic energy enters the diffusion region is higher on the high field side: $(V_{{\mathrm{in}},2} B_{x2}^{2}/8 \pi )/ (V_{{\mathrm{in}},1} B_{x1}^{2}/8 \pi ) \sim B_{x2}/B_{x1}$. Since no magnetic flux passes through the X-line, the X-line is displaced in the inflow direction toward the low magnetic field side so that the distance from the X-line to each side, $\delta _{X1}$ and $\delta _{X2}$ in Fig. 12, has a ratio $\delta _{X1}/\delta _{X2}\simeq B_{x2}/B_{x1}$.

Similarly, the stagnation point has no particle flux across it. The ratio of the incoming particle flux from the 2-side to the 1-side is $n_{2} V_{{\mathrm{in}},2}/n_{1} V_{{\mathrm{in}},1} \simeq n_{2} B_{x1}/n_{1} B_{x2}$ using Eq. (17). This implies that the stagnation point is offset from the center of the diffusion region toward whichever side has the smaller $n/B$ (Cassak and Shay 2007) as is sketched in Fig. 12. It was further shown that this analysis implies the displacement of the X-line and stagnation point both in the ion diffusion region and the electron diffusion region during collisionless reconnection (Cassak and Shay 2009).

The relative location of the X-line and stagnation point has important implications for the microphysics of reconnection, including the structure of the Hall fields in the ion diffusion region, transport of plasma through the diffusion region, and energizing the plasma. The discussion above treated only asymmetries in the inflow direction. It has been similarly shown that an asymmetry in the outflow direction leads to the X-line and stagnation point being displaced in the outflow direction (Oka et al. 2008; Murphy et al. 2010).

### Guide Field Reconnection

Theories in the previous two subsections (the symmetric case in Sect. 3.1 and the asymmetric case in Sect. 3.2) do not include the effect of an external guide field, $B_{g}$, that points out of the reconnection plane. However, in many situations, there is such a magnetic component during reconnection, and we call these cases “guide-field reconnection”. For instance, solar wind magnetic fields (interplanetary magnetic field, IMF) in the magnetosheath plasma can touch Earth’s magnetopause in all possible orientations, making a wide range of magnetic shear angles with respect to the magnetosphere magnetic fields. The strength of the guide field will depend on this magnetic shear angle and the X-line orientation, as illustrated in Fig. 25(b) in Sect. 3.8.1, and also discussed in Gershman et al. (2024, this collection).

#### Theory and Simulations

**Fig. 14:**
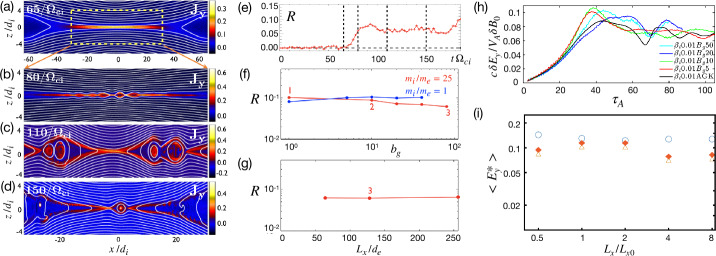
*Guide field reconnection simulations*. (a)-(d) Evolution of an ion-scale current sheet with the guide field strength $b_{g}\equiv B_{g}/B_{R}=8$, white contours are flux surfaces. They correspond to the four different times indicated as vertical dashed lines in panel (e), where the time evolution of the normalized reconnection rate is shown. (a) shows the formation of the intensified out-of-plane current density arising from the initial perturbation before the onset of reconnection; (b) formation of secondary magnetic islands within this sheet; (c) coalescence of magnetic islands and their ejection from the X-line; (d) formation of another secondary island. Panels (f) and (g) show the results of runs where the initial current sheets are on the electron scale. Panel (f) shows the rate $R$ as a function of guide field $b_{g}$ for a fixed system size $L_{x}/d_{e}=128$, while panel (g) shows the rate as a function of system size for fixed guide field $b_{g}=80$. Reprinted from Liu et al. (2014b), with the permission of AIP Publishing. Panel (h) shows the time-evolution of reconnection rate with different $b_{g}$ in gyrokinetic simulations. Adapted from TenBarge et al. (2014), with the permission of AIP Publishing. Panel (i) shows the comparison of reconnection rates between PIC and the two-fluid model in different system sizes. Adapted from Stanier et al. (2015b,a), with the permission of AIP Publishing. They all show a reconnection rate $R$ on the order $\mathcal{O}(0.1)$

Early studies of guide field reconnection were actually motivated by the Sawtooth crashes in fusion devices (von Goeler et al. 1974; Kadomtsev 1975; Aydemir 1991, 1992; Denton et al. 1987; Yamada et al. 1994; Biskamp and Drake 1994; Beidler and Cassak 2011). Reduced fluid simulations (Kleva et al. 1995) suggest the importance of the ion sound Larmor radius $\rho _{s}=\sqrt{k_{B}T_{e}/m_{i}}/\Omega _{ci}$, that is, the ion gyro-radius based on the electron temperature. (Some authors use the total temperature $T_{e}+T_{i}$ in the definition of the ion sound Larmor radius, e.g., Rogers et al. (2001)). This kinetic spatial scale is a different ion length scale than what appears in anti-parallel reconnection, namely where $\nabla _{\|} P_{e\|}$ contributes significantly to $E_{\|}$ in the generalized Ohm’s law (Eq. (2)); more complete review on this scale and the relevant diffusion region signature can be found in Ji et al. (2023, this collection). Fluid simulations show that fast reconnection with an open geometry can be realized when $\rho _{s}$ is much larger than the resistive current sheet thickness of the Sweet-Parker solution and the electron inertial scale $d_{e}$ (Aydemir 1991; Biskamp and Drake 1994; Cassak et al. 2007). If $\rho _{s}$ is smaller than these length scales, the current sheet tends to form an elongated Sweet-Parker-type layer but is often prone to secondary island generation, as seen in panels (a)-(d) in Fig. 14 (Drake et al. 2004; Liu et al. 2014b; Stanier et al. 2015b).

In the collisionless limit, there is no dispute that reconnection with a guide field of order $B_{x0}$ or smaller has a similar reconnection rate $R\sim \mathcal{O}(0.1)$ as antiparallel reconnection. However, no consensus has been reached on the large guide field limit. It was suggested that the reconnection rate drops when the guide field weakens the dispersive property of the kinetic Alfvénic wave (KAW) (Rogers et al. 2001; Tharp et al. 2013), that drives the reconnection outflow. However, in several later simulations, including PIC (Liu et al. 2014b), gyrokinetic (TenBarge et al. 2014), and reduced two-fluid models (Huba 2005; Stanier et al. 2015a,b), the reconnection rate $R\equiv c E_{R}/B_{R}V_{A}$, that is normalized to the reconnecting component, appears to be insensitive to a strong guide field that modifies the dispersive nature of the kinetic Alfvén wave (KAW). These results are highlighted in Fig. 14. One should keep in mind that the relevant Alfvén speed $V_{A}\equiv B_{R}/\sqrt{4\pi n m_{i}}$ is based on the reconnecting magnetic field component only, independent of the guide field strength. Even though empirically from simulations, the presence of a guide field may not affect the value of the collisionless reconnection rate significantly in both symmetric and asymmetric reconnection, a first-principles explanation of why it is this case remains missing.

### Reconnection Rate Observation by the MMS Mission

The normalized reconnection rate has been determined from in-situ plasma particle and field measurements in a variety of ways (e.g. Hasegawa et al. 2024, this collection, and references therein), e.g., the normalized reconnection electric field in the diffusion or inflow regions $E_{y}/B_{x0}V_{Ai0}$, the normal magnetic field component in the exhausts $B_{z}/B_{x0}$, the ion inflow speed in the asymptotic inflow region $V_{iz0}/V_{Ai0}$, the electron inflow speed at the inflow edge of the electron diffusion region (EDR) $V_{ez}/V_{Ae}$, the magnetic flux transport rate across the separatrices $\partial A_{y}/\partial t$, the opening angle of the separatrices (see Eq. (12)), the aspect ratio of the EDR $(\partial B_{z}/\partial x)/(\partial B_{x}/\partial z)$, etc. Here, all quantities are evaluated in the co-moving frame of the X-line, and subscript “0” denotes quantities evaluated in the asymptotic inflow region. For most observations of reconnection, only a small number of these methods may be applicable, depending on where, relative to the X-line, the spacecraft collected measurements and the types of measurements that were made.

Having a large number of rate measurements is a necessary foundation for determining how the background plasma conditions impact the rate. However, reconnection rate measurements typically are associated with large error bars, making comparative analysis difficult. Errors can arise from the determination of the appropriate coordinate system; for instance, $E_{y}$ in the EDR is significantly smaller than the normal electric field $E_{z}$, meaning that small errors in the coordinate axes can lead to large errors in the rate (e.g. Genestreti et al. 2018b). Additionally, errors may be introduced by the determination of the co-moving frame of the X-line; for instance, this frame velocity is often comparable to the upstream ion inflow speed at the magnetopause or magnetotail current sheets. Lastly, remote quantities that are determined by spacecraft far from the X-line (e.g., quantities determined in the inflow region that are used for normalization) are difficult to associate with measurements near the EDR when reconnection is time-varying and/or occurring in spatially inhomogeneous plasma conditions.

Nevertheless, in many ways, MMS data are ideally suited for determining the reconnection rate. Unlike previous missions, MMS particle measurements are made at a rapid enough cadence to resolve the EDR, the spacecraft measures the full 3-D electric field vector, and the tightly-spaced tetrahedral formation of four spacecraft allows gradients of plasma quantities to be determined accurately. Below, we first review the reconnection rate observations derived by various methods listed above for symmetric antiparallel reconnection cases, followed by the reconnection rate observations for different background conditions, such as the asymmetry across the current sheet and the external guide field strength.

#### Rate Observations for Symmetric Anti-Parallel Reconnection

The EDR crossing observation shown in Fig. 3 of Sect. 2.2 took place when the average inter-probe separation was approximately 17 km. It is about half of the asymptotic electron inertial length, $d_{e} \simeq 30\text{ km}$. This close spacecraft distance enabled the application of multi-point analysis methods to determine the detailed characteristics of the current sheet for this event in a quantitative way, such as current sheet orientation, structure, and the spacecraft orbits within the EDR. Genestreti et al. (2018b) estimated the reconnection rate $R = E_{M}/B_{L0}V_{Ai0}$ for this event by using several techniques to find the out-of-plane, $M$, direction along the reconnection electric field and estimated also the error bars using virtual data from a 2D PIC simulation (Nakamura et al. 2018) performed using the initial conditions from the observation.

**Fig. 15:**
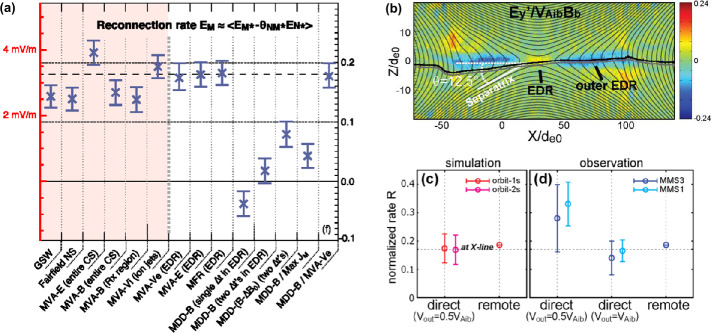
*Reconnection rate estimation for symmetric anti-parallel reconnection event on 11 July 2017*. (a) The reconnection rate in the X-line frame determined over the period 22:34:03–22:34:04 UT. The error bars mark the standard deviation of the reconnection rate over this period. The reconnection rate determined from near-EDR separatrix method (Nakamura et al. 2018) is marked by the long dashed horizontal line. The data in the red-shaded region are determined using coordinate systems that are not solely based on MMS data from within the EDR. GSW = solar-wind-aberrated geocentric solar magnetospheric; MVA = minimum variance analysis; MDD = maximum directional derivative; MFR = minimization of Faraday residue. Adapted from Genestreti et al. (2018b). (b) Simulated $E_{y}'$ (or $E_{R}$) with the in-plane field lines and the paths of two MMS virtual orbits. The opening angle $\theta $ of the separatrix from the simulation is estimated to be 12.5 degree. The normalized reconnection rates $R$ directly obtained from the electric field near the EDR and remotely estimated at the separatrix (c) for two virtual orbits (red and magenta) in the simulation shown in panels (b) and (d) from the MMS3 (blue) and MMS1 (cyan) observations. Adapted from Nakamura et al. (2018)

Figure 15 shows the reconnection electric field $E_{M}$ (left axis) and normalized reconnection rate (right axis) estimated using different analysis methods to obtain the $LMN$ coordinate systems, as reviewed in Hasegawa et al. (2024, this collection). The average values for the upstream Alfvén speed and lobe magnetic field, $B_{L0}$ and $V_{Ai0}$, were used for the normalization: $B_{L0}V_{Ai0} = 18.12\text{ mV/m}$. $E_{M}$ is further corrected to minimize the contamination from the large Hall field ($E_{N}$) in the estimation. A similar reconnection rate was obtained from different methods after reasonable adjustments were performed, and the normalized rate ranged between 0.14 and 0.22. The estimated reconnection rate is $E_{M} = 3.2\text{ mV/m} \pm 0.6\text{ mV/m}$, which corresponds to a normalized rate of $R = 0.18 \pm 0.035$. This value well agrees with the normalized reconnection rate $R = E_{M}/B_{L0}V_{Ai0} = 0.18$ inside the simulated EDR by Nakamura et al. (2018) as shown in Fig. 15c, which was obtained for the virtual MMS trajectory inside the simulation shown in Fig. 15b. This value is also consistent with the reconnection rate, $R = 0.1\text{-}0.2$, approximated using the aspect ratio, which is estimated from the scale-size of the current sheet from the spacecraft motion inside the EDR and the average current density (Torbert et al. 2018).

Nakamura et al. (2018) showed that the observed reconnection rate was consistent with that found in the simulation. Furthermore, the reconnection rate was estimated from the slope of the separatrix using a method introduced by Liu et al. (2017) (see Sect. 3.1.2) giving $R = 0.186$ for the simulation and, from the MMS observations, $R = 0.17$ as shown in (Fig. 15c). Hasegawa et al. (2019) obtained the opening angle of the separatrix field line from the 2D map of the magnetic field and electron streamlines by applying the electron-MHD (EMHD) reconstruction method (see details in Hasegawa et al. 2024, this collection) and obtained a similar reconnection rate, $R= 0.17$. The aspect ratio was also determined directly from the magnetic field gradients by Heuer et al. (2022) for three magnetotail symmetric anti-parallel reconnection events, including the 11 July 2017 event and a similar reconnection rate, $R= 0.1\text{-}0.2$, was obtained.

**Fig. 16:**
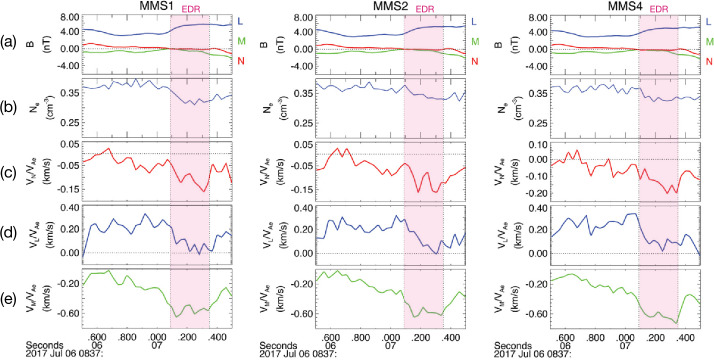
*Plasma and field parameters near the EDR edge.* The transparent red bands mark the EDR for MMS1, 2, and 4. (a) Magnetic field LMN components. (b) Electron density. (c) $V_{eN}$/$V_{AeL}$. (d) $V_{eL}/V_{AeL}$. (e) $V_{eM}/V_{AeL}$, where $V_{AeL}$ is the mean electron Alfvén speed with $B=B_{L}$ for each spacecraft over the first half of each plot (08:37:06.5–08:37:07.0 UT). Adapted from Burch et al. (2022)

Burch et al. (2022) determined the normalized reconnection rate from the inflow velocities normalized to the electron Alfvén speed ($V_{Ae}$) at the edge of the EDR, and compared with other methods during another magnetotail (symmetric) reconnection event on 6 July 2017. Figure 16 shows the plasma and field parameters near the EDR region. The vertical lines show the edge of the EDR (i.e., red transparent bands) for each spacecraft. For this event, the spacecraft was northward of the current sheet, and the converging inflow toward the current sheet center can be seen in the negative $V_{N}$. The normalized reconnection rates derived from the electron inflow velocity measurements, $V_{N}/V_{AeL}$, were 0.11–0.14 using average values of the inflow among 3 MMS spacecraft and 0.15-0.20 when maximum inflow velocity values were used. In comparison, $E_{M}$ normalized to the lobe inflow quantities $V_{iA}B_{L}$ indicates reconnection rates of 0.1-0.17. If $E_{M}$ is normalized to the EDR inflow quantities $V_{eA}B_{L}$, a lower reconnection rate, around 0.06, was found.

#### Current Sheet Structures and Rate Observations for Asymmetric and/or Guide Field Reconnection

**Fig. 17:**
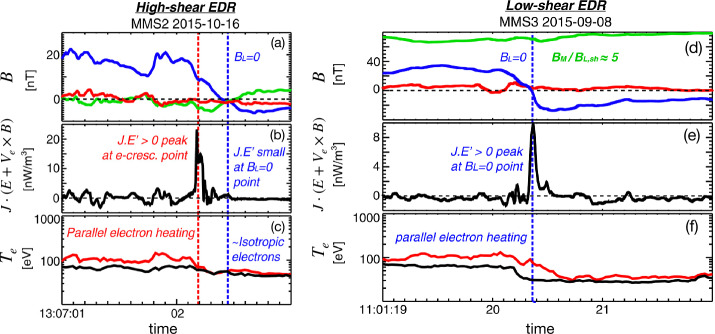
*Observation of asymmetric reconnection events for antiparallel and guide field geometry.* (a, d) The L(blue), M(green), N(red) components of magnetic fields, (b, e) the local energy conversion rate, ${\mathbf{J}}\cdot{\mathbf{E'}}$, (c, f) the parallel (red) and perpendicular (black) electron temperatures. The vertical dashed blue and red lines mark the $B_{L} = 0$ point and the electron “crescent point”. Adapted from Genestreti et al. (2017)

As discussed in the two previous sections, the background asymmetry across the current sheet and/or the existence of the guide fields significantly modifies the structure of the reconnection current sheet, an effect that has been identified in observations. Figure 17 shows two examples of MMS observations from magnetopause reconnection events. The left panels show an event on 16 October 2015 with anti-parallel field geometry, i.e., $B_{M}/B_{L} \simeq 0.1$, at the magnetosheath and a large asymmetry, i.e., the magnetosheath to magnetosphere density ratio $n_{sh}/n_{sp}= 16$. The right panels show an event on 8 September 2015 that has a strong guide field $B_{M}/B_{L} \simeq 5$ and a smaller density asymmetry, $n_{sh}/n_{sp}= 2.5$.

The 16 October 2015 event (left panels) was first reported by Burch et al. (2016b). The current sheet crossing took place from the magnetosphere (low density) to the magnetosheath (high density). The most pronounced feature of asymmetric reconnection is the deviation between the X-point where the $B_{L}=0$ (blue line) and the electron “crescent point” (red line), where the electron velocity distribution function (VDF) has a crescent shape indicating non-gyrotropic distribution in the flow stagnation point; this special point coincides with the peak ${\mathbf{J}}\cdot{\mathbf{E'}}= {\mathbf{J}}\cdot ({\mathbf{E}} + {\mathbf{V}}_{e} \times { \mathbf{B}}/c)$, that was used to measure the dissipation (Zenitani et al. 2011b).

The 8 September 2015 event (right panels of Fig. 17), first reported by Eriksson et al. (2016b), on the other hand, shows a clear peak in the energy conversion rate around the X-point, $B_{L}=0$ due to the dominant parallel components of the current and the electric field. Such features of the strong guide field event (i.e., $B_{M}/B_{L}>0.5$) were obtained also in a statistical study of the energy conversion rate in the magnetosheath reconnection by Wilder et al. (2018). There was no significant non-gyrotropic electron distribution detected at the X-point during the 8 September 2015 event, indicating the effect of small gyroradius relative to the scale size of the current sheet. In modest guide field events, such as the one reported by Chen et al. (2017), energy conversion rate enhancement takes place both at the X-line and the flow stagnation point, and the parallel heating of electrons occurs at both locations. Overall, the separation of the flow stagnation point and the X point is found with density asymmetry and magnetic field asymmetry (Genestreti et al. 2017), consistent with the prediction in Sect. 3.2.3.

Although the asymmetry, as well as the guide field, significantly modifies the structure of the reconnection current sheet, the observed range of reconnection rates is similar to that in standard anti-parallel symmetric reconnection. Burch et al. (2020) determined the normalized reconnection rate from the electron inflow velocities $V_{eN}$ for four MMS events, including three previously published crossings (Chen et al. 2017; Phan et al. 2018; Pritchard et al. 2019), and obtained values between 0.05 and 0.25. Among these four events, one event was an “electron-only” reconnection event in the magnetosheath (Phan et al. 2018) that will be discussed in the next section.

A survey of asymmetric reconnection rates has been performed by Pritchard et al. (2023), including seven magnetopause events that show values of $0.14\pm 0.09$ and seven magnetosheath events that show values of $0.16\pm 0.12$. There was no correlation between the normalized reconnection rate and guide field, as has been suggested by simulation (see Sect. 3.3.1). A finite guide field has been also reported for reconnection events in the magnetotail in a current sheet with wave fluctuations (Chen et al. 2019) and varying guide field, 0.14-0.5; the normalized reconnection rate ranges between 0.05 and 0.3. A transient current sheet at the dipolarization front (Hosner et al. 2024) with a guide field 1.8 shows a normalized reconnection rate of 0.16-0.18, which is comparable to that observed during reconnection at the magnetopause and in the magnetosheath.

### Electron-Only Reconnection

#### Observational Evidence

In turbulent plasma, reconnection has long been suggested to play a role in the dissipation of turbulent energy (e.g., Matthaeus and Lamkin 1986; Servidio et al. 2009). The turbulent magnetosheath region downstream of Earth’s quasi-parallel bow shock often contains hundreds of small-scale current sheets in which reconnection could occur (Retinò et al. 2007; Sundkvist et al. 2007; Yordanova et al. 2016; Vörös et al. 2017; Wilder et al. 2018). If standard reconnection were to operate in turbulent current sheets, the ion jets in the extended exhausts would be the easiest reconnection signature to detect. However, the ultra-high time resolution plasma and field measurements of MMS have revealed a lack of ion scale exhausts, although some electron jets were observed. It was suggested that this implies the existence of a new form of reconnection in which ions do not participate, but electrons do. This was dubbed “electron-only” reconnection (Phan et al. 2018; Stawarz et al. 2019, 2022).

**Fig. 18:**
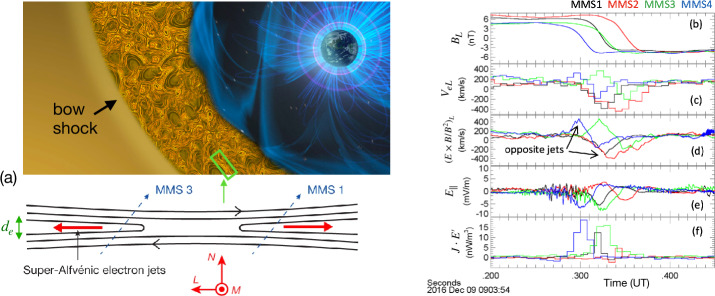
*Observation of electron-only reconnection*. (a) Schematics showing electron-only reconnection in an electron-scale current sheet embedded in turbulent structures downstream of Earth’s quasi-parallel shock, (b) reconnecting magnetic field component (L), (c) electron outflow velocity, (d) $E\times B$ velocity in the outflow direction, (e) parallel electric field, and (f) non-ideal energy conversion ${\mathbf{J}}\cdot ({\mathbf{E}}+{\mathbf{V}}_{e}\times {\mathbf{B}}/c)$. MMS 3 and 4 observed positive $V_{eL}$ outflow jets, while MMS 1 and 2 observed negative $V_{eL}$ jets inside the current sheet. Adapted from Phan et al. (2018), reproduced by permission of Springer Nature. The illustration in Panel(a) is credited to NASA’s Goddard Space Flight Center

In this type of reconnection, the electron outflow jets from the reconnection X-line have speeds comparable to the electron Alfvén speed based on $B_{L}$ upstream of the electron diffusion region, and the current sheet width is substantially narrower than the ion Larmor radius or ion inertial scale. Importantly, in contrast to the electron diffusion region of standard reconnection, electron-only reconnecting current sheets are not embedded inside ion-scale current sheets (Phan et al. 2018). Figure 18 shows a fortuitous event where pairs of MMS spacecraft simultaneously detected oppositely directed super-ion-Alfvénic electron outflow jets emanating from an X-line (Fig. 18(c), (d)) in an electron-scale current sheet (Fig. 18(b)) (Phan et al. 2018). Strong parallel electric fields (Fig. 18(e)) and enhanced energy conversion (Fig. 18(f)) were present in the current sheet. This current sheet was one of hundreds of electron-scale current sheets in a 10-minute interval downstream of a quasi-parallel shock. Analysis of the statistical properties of this and other magnetosheath intervals measured by MMS reveals that the presence of electron-only reconnection is linked to the correlation length of the turbulence (i.e., the driving scale of the turbulence), with the correlation length of the electron-only events being several ion inertial lengths or less (Stawarz et al. 2019, 2022). These observations suggest that electron-only reconnection occurs in small-scale current sheets when there is insufficient space and/or time for the ions to couple to the reconnected magnetic field.

MMS has also detected sites of magnetic reconnection within the bow shock transition layer itself (Gingell et al. 2019; Wang et al. 2019). The predominantly electron-only reconnection events in the shock disentangle the turbulent shock fields and may contribute to the overall shock heating. Along with complementary studies quantifying how current sheets and reconnection in the magnetosheath fit into the energy budget (Schwartz et al. 2021) and are influenced by the properties of the bow shock (Bessho et al. 2019; Gingell et al. 2020; Yordanova et al. 2020; Bessho et al. 2022), MMS has brought together three fundamental areas of plasma physics research – turbulence, shocks, and reconnection. In addition to the bow shock and magnetosheath, MMS has measured electron reconnection in other contexts, most of which involve kinetic-scale turbulent structures. These included foreshock transients (Liu et al. 2020b), electron-scale substructures inside macro-scale magnetic flux ropes (Man et al. 2020), reconnection exhausts (Huang et al. 2021; Norgren et al. 2018), magnetotail dipolarization fronts (Marshall et al. 2020), and the early phase of magnetotail reconnection (Lu et al. 2020). See also the review by Hwang et al. (2023, this collection) for further discussion of many of these phenomena. These findings suggest that electron-only reconnection is prevalent in kinetic-scale current sheets in space plasmas and could play an important role in the dissipation of turbulence energy. In the wake of this MMS discovery, electron-scale reconnection is now also studied in laboratory experiments (Shi et al. 2022; Greess et al. 2022; Chien et al. 2023; Shi et al. 2023).

#### Theory

**Fig. 19:**
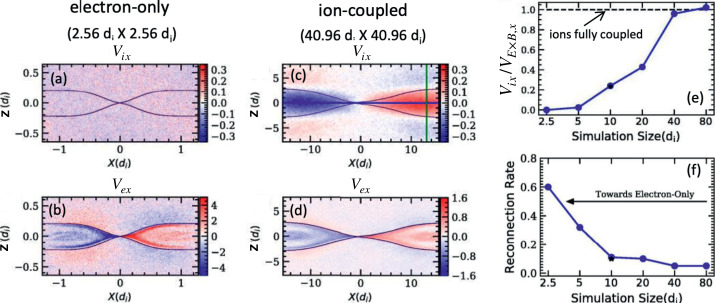
*Transition from standard ion-coupled reconnection to electron-only reconnection*. (a, b) Ion and electron velocities in the exhaust (x or L) direction for a small 2D PIC simulation. Velocities are normalized to the asymptotic upstream proton Alfvén speed $V_{A}$, and $V_{Ae}=42.8V_{A}$ in this simulation. (c, d) ion and electron velocities for a larger simulation, (e) ion outflow velocity normalized to the $E \times B$ velocity as a function of simulation domain size, and (f) reconnection rate $R$ (normalized to $B_{x0}V_{A}$) versus simulation domain size. Adapted from Pyakurel et al. (2019)

A hybrid simulation study of resistive reconnection with no guide field by Mandt et al. (1994) found that ions become decoupled from the magnetic field when the length of the current sheet (in the outflow direction) falls below ${\sim} 10 d_{i}$. Such reconnection without ion-coupling has also been modeled using electron magnetohydrodynamics (EMHD) simulations (Jain and Sharma 2009, 2015). Prompted by the MMS observation (Phan et al. 2018), Pyakurel et al. (2019) investigated the transition from ion-coupled to electron-only reconnection using particle-in-cell (PIC) simulations by varying the simulation domain size systematically. They found that the transition from fully ion-coupled to electron-only reconnection is gradual. This transition is characterized by a gradual increase in the degree to which the ions are frozen-in to the magnetic field as the simulation box size increases, with the ions being fully coupled (${\mathbf{V}}_{i\perp} \simeq {\mathbf{E}}\times {\mathbf{B}}/B^{2}$) when the box size reaches $40 d_{i}$ (Fig. 19(e)); note that the boundary conditions are periodic in the outflow direction. On the other hand, ion outflows are weakly coupled to the magnetic field when the domain size is below $20 d_{i}$ and clearly not coupled below $5 d_{i}$. Another study suggested that it is the ion gyro-radius, instead of the inertial scale, that sets the transition to electron-only reconnection (Guan et al. 2023).

Figures 19(a), (b) show an example of the ion and electron velocity along the outflow direction for the smallest simulated domain of $2.56 d_{i} \times 2.56 d_{i}$. The electron outflows are super-ion-Alfvénic outflows (Fig. 19(b)), while there are essentially no ion outflows (Fig. 19(a)). On the other hand, the simulation with a large domain size of $40.96 d_{i} \times 40.96 d_{i}$ exhibits standard ion-coupled reconnection with both ion and electron outflows (Fig. 19(c)-(d)).

Since ions are much more massive than electrons, they remain more or less immobile ($V_{i}\simeq 0$) compared to electrons within a small spatial and temporal scale, as shown in Fig. 19(a), and the rate of work done on ions ($en{\mathbf{E}}\cdot {\mathbf{V}}_{i}$) thus becomes negligible. The majority of magnetic energy is converted to electron energy. The dynamics are then described by the steady-state electron momentum equation,

26 $$ \frac{({\mathbf{B}}\cdot \nabla ){\mathbf{B}}}{4\pi} - e n{\mathbf{E}}\simeq nm_{e}({ \mathbf{V}}_{e}\cdot \nabla ){\mathbf{V}}_{e}, $$

where the magnetic tension works to drive the electron outflow. If we just consider tension and electron inertia, this equation results in electron Alfvénic jets with speed

27 $$ V_{\mathrm{out}}\simeq V_{Ae}\simeq \frac{B_{x0}}{\sqrt{4\pi n m_{e}}}. $$

The Hall electric field arising from the decoupling between the two species gives a positive $enE_{x}$ in Eq. (26), which slows down electrons while speeding up ions (i.e., tries to couple electrons and ions again). This partially explains why the peak electron outflow speed $V_{ex}$ is super-ion-Alfvénic but sub-electron-Alfvénic as shown in Fig. 19(b). The back-pressure (not included in Eq. (27)) arising from the periodic boundaries in the outflow direction may also limit the outflow speed, especially within such a small system. For ions, Pyakurel et al. (2019) show that the reduction of the ion outflow speeds as a function of the system’s size compares well with the prediction from the “standing wave” approximation, where the Hall effect dominates (Mandt et al. 1994; Rogers et al. 2001; Drake et al. 2008).

Since magnetic flux remains frozen-in to electrons, the magnetic flux transport speed that determines the reconnection rate is now not limited by the ion Alfvén speed but by the faster electron Alfvén speed. The higher flux transport speed (Eq. (27)) will make the normalized rate R (that is normalized to the proton Alfvén speed) higher than $\mathcal{O}(0.1)$, consistent with the simulated rate in the small system size limit, as shown in Fig. 19(f). If the EDR aspect ratio remains on the order of ${\sim} 0.1$, a rough estimate of the normalized rate is $R\le 0.1\sqrt{m_{i}/m_{e}}=\sqrt{1836}\times 0.1= 4.3$, which can only be regarded as the upper bound value because the simulated value appears to be smaller than unity (Fig. 19(f)). An analytical model better than this simple estimation needs to be derived.

Given that electron-only reconnection tends to occur in plasma environments where the magnetic structure correlation length is small (several ion inertial lengths or less) (Stawarz et al. 2022), such structures tend to be highly 3D in nature. Pyakurel et al. (2021) found that the reconnection rate in 3D electron-only reconnection (with a finite X-line) is higher than in 2D. This is because, in addition to reconnection outflows in the standard exhaust direction, there is a differential mass flux out of the diffusion region along the X-line direction, enabling a faster inflow velocity and, thus, a larger reconnection rate. The theoretical findings of higher reconnection rates in 3D electron-only reconnection further suggest that it could play an important role in the dissipation of turbulence energy.

Observationally, Burch et al. (2022) reported a normalized reconnection rate for the Phan et al. (2018) electron-only reconnection event using measurement of the inflow velocity and obtained a value of $0.25\pm 20\%$. Pritchard et al. (2023) used measurements of the reconnection electric field for this event to determine a very similar normalized reconnection rate of $0.23\pm 43\%$. These values are at the high end of theoretical prediction, but more measurements are needed to determine whether the reconnection rates are significantly higher than for ion-coupled reconnection.

### Reconnection with Heavy and Cold Ions

Space plasmas often have multiple ion populations, including, for instance, ions heavier than protons, or proton beams that are colder than the background protons. In this subsection, we discuss the effects of multiple ion populations on the magnetic reconnection rate and the extension of the generalized Ohm’s law to such plasmas.

#### Theory

It is of interest to understand how reconnection physics is affected when multiple ion species co-exist in a plasma. Similar to the treatment of a typical two-species (electron-proton) plasma, we can combine the momentum equations of multiple species into a single force balance equation,

28 $$ \frac{({\mathbf{B}}\cdot \nabla ){\mathbf{B}}}{4\pi}\simeq \sum _{s}^{1,2,\ldots} n_{is} m_{is}({\mathbf{V}}_{is}\cdot \nabla ){\mathbf{V}}_{is}, $$

where $m_{is}$ and $n_{is}$ represent the ion mass and density of species “s”, respectively. The ion charge $q_{is}$ and density satisfy $\sum _{s} q_{is} n_{is}\simeq n_{e}\equiv n$ for quasi-neutrality. Each ion species will have its own diffusion region (Shay and Swisdak 2004). Given a sufficiently large system, all ions will become frozen-in to the reconnection outflow outside the outermost diffusion region. With heavier ions, this will occur at larger spatial scales and longer timescales than that in the typical electron-proton plasma. The increased mass load at outflows can significantly limit the outflow speed; i.e., note that a proton is an ion of the lowest mass. The outflow speed scales as the Alfvén speed based on the effective mass density $\rho _{\mathrm{heavy}}=\sum _{s} n_{is}m_{is}$

29 $$ V_{\mathrm{out}}\simeq V_{A,\mathrm{heavy}}= \frac{B_{R}}{\sqrt{4\pi \rho _{\mathrm{heavy}}}}. $$

Thus, based on this full mass load and the $\delta /L\sim \mathcal{O}(0.1)$ assumption, the reconnection rate normalized to $V_{A,heavy}$ is expected to be $\mathcal{O}(0.1)$. If one normalized $E_{R}$ to the proton Alfvén speed, a lower value is expected. However, if reconnection occurs within a small spatial domain and short time scale, the heavy ions can become decoupled, and the outflowing flux transport speed is not constrained by the Alfvén speed in Eq. (29). The reconnection electric field can thus be higher than the expected $0.1 B_{R} V_{A,\mathrm{heavy}}$. This situation resembles “electron-only” reconnection, as discussed in Sect. 3.5, where protons are not fully coupled.

#### Results from Simulations and Observational Evidence

##### Heavy Ions

**Fig. 20:**
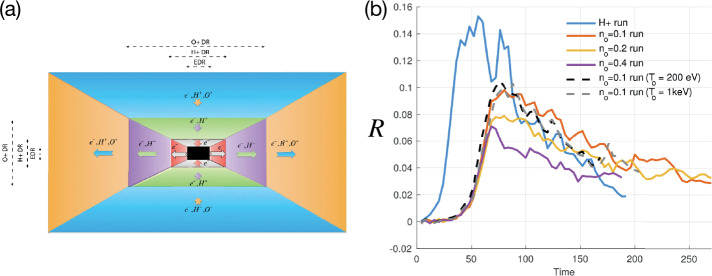
*Reconnection rate for various amounts of oxygen (i.e., heavy ion) in PIC simulations*. (a) multi-layered diffusion regions. Adapted from Liu et al. (2015c). (b) Evolution of reconnection electric field in full PIC simulations of symmetric magnetic reconnection with different amounts of $\mathrm{O}^{+}$ (i.e., $n_{O}$). Reprinted from Tenfjord et al. (2019), with the permission of Wiley

Several PIC simulations that include three species (electrons, $\mathrm{H}^{+}$, $\mathrm{O}^{+}$) have shown the differential behavior of lighter and heavier ions near the x-line of magnetic reconnection, resulting in a multi-layered diffusion region with sizes related to the characteristic length-scales of each species, e.g., Shay and Swisdak (2004), Markidis et al. (2011), Liu et al. (2015c), as illustrated in Fig. 20(a). Observational evidence of the multi-layered nature of the DR in the presence of oxygen has also been shown using Cluster (Escoubet et al. 2001) observations in Earth’s magnetotail (Liu et al. 2015c).

Full PIC simulations of magnetic reconnection in Earth’s magnetotail that include $\mathrm{O}^{+}$ have shown that the reduction in reconnection electric field does not really scale with the prediction based on the full mass-load (Shay and Swisdak 2004; Markidis et al. 2011; Tenfjord et al. 2019). Figure 20(b) shows the results of various full PIC simulations of magnetic reconnection with varying amounts of $\mathrm{O}^{+}$, where the time evolution of the reconnection electric field is plotted. The maximum electric field drops with increasing amounts of oxygen. However, the reduction is consistent with

30 $$ R \simeq \frac{0.1}{1+n_{O}/n_{p}}, $$

rather than the reduction expected by the full mass load. i.e., $R\simeq 0.1/[1+m_{O} n_{O}/ (m_{p}n_{p})]^{1/2}$. This discrepancy can be explained by the fact that $\mathrm{O}^{+}$ remains unmagnetized during the typical timescales of the simulations (which are related to the reconnection timescales in the Earth’s magnetotail) (Tenfjord et al. 2019; Kolstø et al. 2020). Therefore, $\mathrm{O}^{+}$ is ballistically accelerated by the non-ideal electric field within its diffusion region, and $\mathrm{O}^{+}$ acts as an energy sink (reducing the reconnection rate), but the reduction is less severe compared with the prediction based on the full mass load. Another interesting conclusion that can be drawn from Fig. 20(b) is that changing the temperature of the $\mathrm{O}^{+}$ population does not have an effect on the reconnection electric field. Finally, it is also noted that for larger domains and longer time scales, when all ions are magnetized outside of the diffusion region, the full mass-load scaling of the reconnection rate is expected.

##### Cold Protons

Another common situation in magnetic reconnection in Earth’s magnetosphere is having two distinct proton populations: a hot (keV-scale) component coming from the plasma sheet plus a cold (eV-scale) component arising from the Earth’s ionosphere. In the following discussion, we refer to “protons” as simply “ions”.

Ions demagnetize at length scales below the ion inertial length or the ion gyroradius. The ratio between the two is given by $\rho _{i}/d_{i} = \sqrt{\beta _{i}}$. For high-$\beta $ plasmas, the gyroradius is larger, while for low-$\beta $ plasmas, the inertial length is larger. At the Earth’s magnetopause, the plasma beta is often of the order of 1, and therefore the two length scales are comparable. When hot and cold protons are present, cold protons have the ability to remain magnetized inside narrow structures such as the separatrix or the (hot) ion diffusion region (Toledo-Redondo et al. 2015, 2018; André et al. 2010). Under this situation, a multi-layered diffusion region is also generated due to the different gyroradius of the two proton populations. The multi-layered nature of the DR has been observed both using PIC simulations (Divin et al. 2016; Dargent et al. 2017, 2020) and MMS observations (Toledo-Redondo et al. 2016).

**Fig. 21:**
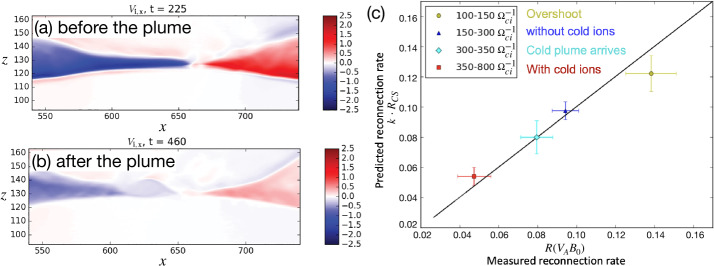
*Reconnection electric field for various amounts of cold ions in a PIC simulation*. Panel (a) and (b) show the ion outflow speed $V_{ix}$ before and after the arrival of the cold plasma plume during asymmetric reconnection. (c) Predicted versus measured reconnection electric fields at various stages model the impact of a cold, dense plume on Earth’s magnetopause reconnection. Except during the overshoot time, the scaling by Cassak and Shay (2007) explains the observed reductions of the reconnection electric field. Reprinted from Dargent et al. (2020), with the permission of Wiley

The inclusion of multiple proton populations leads to a mass-loading reduction of the reconnection electric field. However, the normalized reconnection rate ($E_{R}/V_{A,\mathrm{proton}}B_{R}$) remains unaffected (Divin et al. 2016; Dargent et al. 2017, 2020; Spinnangr et al. 2021). Figure 21(a)-(b) serves to illustrate the mass-loading effect by adding cold protons to magnetic reconnection at a later time. Figure 21(c) compares the measured reconnection rate (horizontal axis) with the predicted reconnection rate using the full mass-load in the Cassak and Shay (2007) scaling, as discussed in Sect. 3.2. This setup of the PIC simulation mimics the impact of a cold, dense plume on the reconnecting Earth’s magnetopause (Dargent et al. 2020). At $t < 150\: \Omega_{ci}^{-1}$, the maximum reconnection rate is reached in the simulation; this time is often referred to as the overshoot time. For $150 < t < 300 \:\Omega_{ci}^{-1}$, reconnection proceeds, but the cold, dense plume has not yet reached the reconnection region (dark blue dot). At $t \simeq 300 \:\Omega_{ci}^{-1}$, the cold, dense plume reaches the reconnection region, starts mass-loading the reconnecting flux tubes, and reduces the reconnection electric field (cyan dot). For $t > 350 \: \Omega_{ci}^{-1}$, more cold ions have entrained reconnection, and the reconnection electric field is even smaller (red dot). Except during the overshoot time (yellow), all measured reconnection electric fields scale well with the predicted asymmetric reconnection electric field (Eq. (22) with the $\delta /L\sim 0.1$ assumption), indicating that the observed reduction corresponds to the effect of mass-load.

When both cold (eV) and hot (keV) proton populations are present in reconnection, Ohm’s law can be expressed as (Toledo-Redondo et al. 2015)

31 $$ {\mathbf{E}}+\frac{n_{ic}}{n}\frac{{\mathbf{V}}_{ic} \times{\mathbf{B}}}{c}+ \frac{n_{ih}}{n}\frac{{\mathbf{V}}_{ih} \times{\mathbf{B}}}{c}= \frac{{\mathbf{J}}\times{\mathbf{B}}}{nec}-\frac{\nabla \cdot {\mathbf{P}}_{e}}{ne}+ \frac{m_{e}}{e^{2}}\frac{d}{dt}\left (\frac{{\mathbf{J}}}{n}\right ), $$

where “c” and “h” indicate cold and hot populations, respectively. The electron density $n_{e} = n \simeq n_{ic} + n_{ih}$, and ${\mathbf{J}} = en_{ic}{\mathbf{V}}_{ic} + en_{ih}{\mathbf{V}}_{ih} -en{\mathbf{V}}_{e}$.

**Fig. 22:**
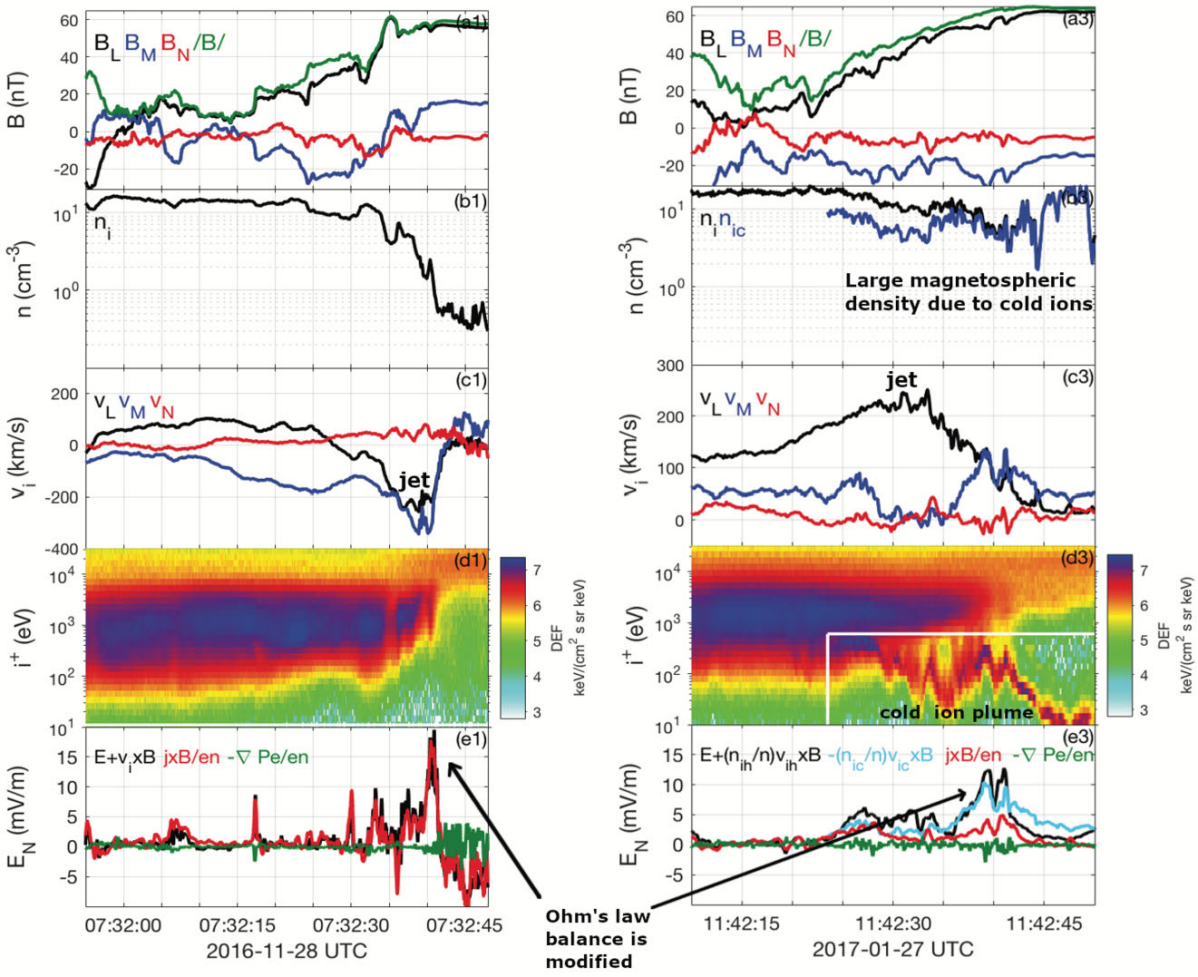
*Generalized Ohm’s law analysis including cold ions at the separatrix of dayside magnetic reconnection.* Comparison of two crossings of the magnetic reconnection separatrices. (a) Magnetic field in LMN coordinates. (b) total ion density and cold ion density. (c) Ion velocity in LMN coordinates. (d) Ion spectrogram. (e) Terms in the generalized Ohm’s law (Eq. (31)). Adapted from Toledo-Redondo et al. (2018), with the permission of Wiley

Figure 22 shows two independent MMS crossings of the reconnecting dayside magnetopause. The magnetic field rotation can be observed in panels a1 and a2. The magnetic field amplitude at the two crossings, both at the magnetosheath and the magnetosphere, is of the same level. The magnetosheath densities are between 10-20 cm^−3^ on the two crossings, but the density on the magnetosphere for crossing 1 (${\sim} 0.5\text{ cm}^{-3}$) is much smaller than for crossing 2 (${\sim} 11\text{ cm}^{-3}$), due to the presence of a cold ion plume in the latter (see panel d2). The ion velocities (panels c1 and c2) show ion jets consistent with the prediction in Eq. (21) (Cassak and Shay 2007). The normal component (N) of the Ohm’s law terms is plotted in panels e1 and e2. For crossing 1, there is no cold ion population ($n_{i}=n_{ih}$, ${\mathbf{V}}_{i}={\mathbf{V}}_{ih}$) and the term ${\mathbf{E}}+{\mathbf{V}}_{i} \times {\mathbf{B}}/c$ is balanced by ${\mathbf{J}}\times {\mathbf{B}}/enc$, while for crossing 2 the term ${\mathbf{E}}+(n_{ih}/n_{i}){\mathbf{V}}_{ih}\times {\mathbf{B}}/c$ is mostly balanced by $-(n_{ic}/n_{i}){\mathbf{V}}_{ic}\times {\mathbf{B}}/c$, and ${\mathbf{J}} \times {\mathbf{B}}/enc$ contributes only a small fraction. The reason is that for crossing 2, the abundant cold ions remain magnetized inside the separatrix layer and reduce the perpendicular currents (Toledo-Redondo et al. 2018).

### High-$\beta $ Reconnection

While more magnetic energy is available in the low plasma $\beta \equiv P/(B^{2}/8\pi ) \ll 1$ regime, reconnection also occurs in systems with high $\beta \gg 1$. Such plasmas can be found in the outer heliosphere ($\beta $ up to 10) (Drake et al. 2010; Schoeffler et al. 2011), at the Galactic center ($\beta \sim 10^{1}-10^{2}$) (Marrone et al. 2007), or in the hot intracluster medium (ICM) of galaxy clusters ($\beta \sim 10^{2}-10^{4}$) (Carilli and Taylor 2002; Schekochihin and Cowley 2006).

#### Theory

In this limit, self-generated pressure anisotropy and/or pressure gradients both upstream (Egedal et al. 2013) and downstream (Bessho and Bhattacharjee 2010; Liu et al. 2011b, 2012; Le et al. 2014; Haggerty et al. 2018) of the diffusion region can affect the force balance that determines the maximum plausible reconnection rate. Thus, unlike in Sect. 3.1.2, we need to include plasma pressure effects in the mesoscale force balance,

32 $$ \nabla \cdot \left (\varepsilon \frac{{\mathbf{B}}{\mathbf{B}}}{4\pi}\right ) \simeq \frac{\nabla B^{2}}{8\pi}+\nabla P_{\perp}+nm_{i}({\mathbf{V}} \cdot \nabla ){\mathbf{V}} $$

where the pressure anisotropy (i.e., firehose) factor $\varepsilon =1-4\pi (P_{\|}-P_{\perp})/B^{2}$ and $P_{\|}$ ($P_{ \perp}$) refers to the pressure component parallel (perpendicular) to the local magnetic field. Using this force balance, one can follow the framework in Sect. 3.1.2 to derive the general $R-S_{\mathrm{lope}}$ relation (Li and Liu 2021).

**Fig. 23:**
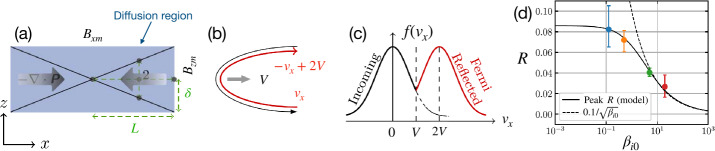
*Including thermal pressure effect in the reconnection rate model.* (a) Illustrates the back-pressure (grey arrows) within the diffusion region, that opposes the outflow. (b) and (c) show how Fermi reflection is revealed in the particle distribution. (d) Shows that the predicted maximum plausible rate (solid curves) explains well the simulated rates (dots) in cases with different plasma-$\beta $. Adapted from Li and Liu (2021), reproduced by permission of the AAS

Specifically, the back-pressure $\nabla P_{\perp}$ can oppose the outflow, and a pressure anisotropy of $\varepsilon < 1$ can weaken the magnetic tension. It was shown that ion Fermi reflections of ions in the outflow region (illustrated in Fig. 23(b)) play the dominant role in increasing the back-pressure (illustrated in Fig. 23(c)) and reducing the reconnection outflow speed in the high-$\beta $ limit. The predicted maximum plausible reconnection rate scales as $R_{max}\simeq 0.1/\sqrt{\beta _{i}}$ in the high upstream ion beta ($\beta _{i}$) limit, comparing well with PIC simulations in Fig. 23(d).

#### Observational Evidence

The same theory (Li and Liu 2021) also makes an important correction to the outflow speed that can be tested using observation. In the low-$\beta $ limit,

33 $$ V_{\mathrm{out}}\simeq V_{{\mathrm{out}},m} \simeq 0.43 V_{A}. $$

**Fig. 24:**
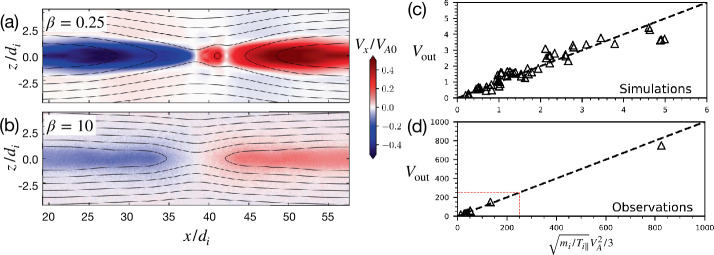
*The scaling of outflow speed as a function of plasma* $\beta $. Ion outflow speeds in (a) low-$\beta $ and (b) high-$\beta $ plasmas Adapted from Li and Liu (2021). In panel (c), the asymptotic $E\times B$ outflow velocity (presuming the ion outflow speed for magnetized ions) versus the outflow speed prediction for PIC simulations and for observations (d) in nearly anti-parallel events. Adapted from Haggerty et al. (2018)

Here subscript “$m$” denotes the outflow edges of the “microscopic” ion diffusion region. The first equality holds in the small-aspect-ratio limit. This prediction explains why the outflow speed is usually around half of the Alfvénic speed, as is often observed in space. In the high-$\beta $ limit, the outflow speed is further reduced

34 $$ V_{\mathrm{out}}\simeq V_{{\mathrm{out}},m}\simeq \frac{\sqrt{\pi}}{4} \frac{\varepsilon _{m}V_{A}}{\sqrt{\beta _{i}}}, $$

where $\varepsilon _{m}$ is the anisotropic parameter at the inflow edge of the IDR. Equation (34) is almost identical to the expression obtained in Haggerty et al. (2018), $V_{\mathrm{out}}\simeq (1/3)\varepsilon _{m} V_{A}^{2}/(T_{i\|}/m_{i})^{1/2}$, which adapted an empirical factor of $1/3$ in their model. Regardless of the difference in the approach, most importantly, both expressions agree well with 81 kinetic simulations and 14 in situ observations that span a wide range of parameter regimes, as shown in Fig. 24(c)-(d).

### Reconnection Suppression by Diamagnetic Drifts and Sheared Flows

In the usual scenario, the outflow directions from a reconnection X-line ($\pm L$ in $LMN$ coordinates) are equivalent, and hence one expects the outflow jets to be symmetric. However, in certain situations the X-line itself can move in one direction or the other. Not only does this motion break the outflow symmetry, but if the motion is sufficiently fast, it can disrupt an outflow jet. When this happens, reconnection itself can be suppressed. This effect is particularly pronounced in two regimes: asymmetric reconnection in which a pressure gradient extends across the current sheet, such as planetary magnetopauses (see e.g. Gershman et al. 2024, this collection, Phan et al. 2013a, and references therein), and reconnection in the presence of shear flows, such as events at the flanks of Earth’s magnetopause (see e.g. Hwang et al. 2023, this collection, and references therein).

#### Diamagnetic Suppression

In the presence of a magnetic field, any non-parallel pressure gradient produces a diamagnetic drift,

35 $$ {\mathbf{V}}^{*}_{s} = -c \frac{{\nabla} P_{s} {\times}{\mathbf{B}}}{q_{s}nB^{2}}, $$

where $P_{s}=nk_{B}T_{s}$ is the thermal pressure and $q_{s}$ is the charge of species $s$. Somewhat famously, ${\mathbf{V}}^{*}_{s}$ is a fluid drift that does not correspond to actual particle motion; nevertheless, it can advect the magnetic field (Coppi 1965; Scott and Hassam 1987). To see this, note that in a system with an invariant direction in $\hat{\mathbf{y}}$ (i.e., $\partial _{y} = 0$), one can write ${\mathbf{B}} = \hat{\mathbf{y}} \times \nabla \psi (x,z) + B_{y}(x,z) \hat{\mathbf{y}}$ where $\psi $ is the magnetic flux function. Taking the cross product of Faraday’s law with $\hat{\mathbf{y}}$ yields $\partial _{t} \nabla \psi -c \nabla E_{y}=0$, or $E_{y} = \partial _{t} \psi /c$.

Next, dotting the electron momentum equation (Eq. (1)) with $\hat{\mathbf{y}}$ gives

36 $$ E_{y} = -\frac{1}{c} \hat{\mathbf{y}} \cdot ({\mathbf{V}}_{e} \times {\mathbf{B}})+ E_{y, \text{non-ideal}}. $$

The last term represents the non-ideal electric field that breaks the electron frozen-in condition. For simplicity, we ignore it and substitute for ${\mathbf{B}}$ to get $E_{y} = -{\mathbf{V}}_{e} \cdot \nabla \psi /c$ that leads to an advection equation for the flux (Coppi 1965),

37 $$ \partial _{t} \psi + {\mathbf{V}}_{e} \cdot \nabla \psi = 0. $$

Hence, the electron fluid velocity, which includes a diamagnetic component given by Eq. (35), advects magnetic structures. [Note that if one retains the $E_{y,\text{non-ideal}}$ term that can cause slippage between electrons and magnetic flux within the EDR, the result is the Magnetic Flux Transport (MFT) velocity $U_{\psi}$ (Liu and Hesse 2016; Liu et al. 2018b; Li et al. 2021a), that is basically the $E\times B$ drift speed based solely on the in-plane magnetic component; also discussed in Hasegawa et al. (2024, this collection).]

**Fig. 25:**
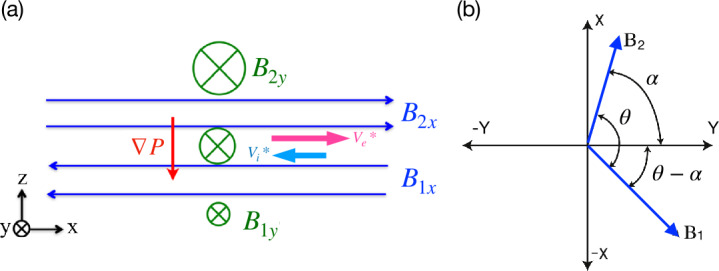
*Definition of the coordinate system*. (a) The guide field and pressure gradient give rise to diamagnetic drifts during reconnection on the $x$-$z$ plane. Adapted from Liu and Hesse (2016). (b) The view along the inflow ($z$) direction. Given the total magnetic shear angle $\theta $, the angle $\alpha $ that tells the orientation of the x-line is, in general, unknown. Adapted from Swisdak and Drake (2007), with the permission of Wiley

Consider then, on a qualitative level, the effect of such a drift on a reconnecting X-line and specifically on the (ion) Alfvénic outflows (in the $\pm x$ direction in the current coordinate system), as shown in Fig. 25(a). A diamagnetically drifting X-line will move in the same direction as one of the two outflows and, for certain parameters, the drift speed can exceed the outflow velocity. This case is roughly analogous to shock propagation in that the X-line’s motion is rapid enough that the X-line itself arrives downstream before any newly reconnected field lines. As numerical simulations have shown (Swisdak et al. 2003), the net effect is to choke off and suppress reconnection. The stability criterion is basically,

38 $$ \lvert V_{e}^{*}-V^{*}_{i} \rvert > V_{A}, $$

where $V^{*}_{e}$ and $V^{*}_{i}$ are the electron and ion diamagnetic velocities, respectively. A simple relationship quantifying when such suppression should occur– particularly one that depends only on upstream parameters – would be useful for spacecraft observations. To derive such a condition, begin with a system characterized by magnetic field vectors ${\mathbf{B}}_{1}$ and ${\mathbf{B}}_{2}$, number densities $n_{1}$ and $n_{2}$ and pressures $P_{1}$ and $P_{2}$ on either side of a current layer. The relation $\cos \theta = {\mathbf{B}}_{1} \cdot {\mathbf{B}}_{2}/B_{1}B_{2}$ defines the angle $\theta $ between the asymptotic field, with $\theta = 180^{\circ}$ corresponding to anti-parallel reconnection. The coordinate system, with the unknown angle $\alpha $, is shown in Fig. 25(b): $B_{1x}$ and $B_{2x}$ are the reconnecting components of the field, and the respective guide fields are $B_{1y}$ and $B_{2y}$. The X-line points parallel to $\hat{\mathbf{y}}$ while reconnection occurs in the $x-z$ plane.

The stability criterion is obtained in two steps. The first requires determining the direction of the X-line (or, equivalently, the plane in which reconnection occurs) since this choice affects the field components that enter the calculation of $V^{*}_{e}$. Determining this orientation has been the subject of multiple papers (Swisdak and Drake 2007; Schreier et al. 2010; Hesse et al. 2013; Liu et al. 2015b, 2018c) with no clear resolution, but while the exact results differ, there is general agreement that, to a reasonable approximation, the reconnection X-line bisects the angle $\theta $ between the two magnetic fields (Hesse et al. 2013; Liu et al. 2018c) (in other words, $\alpha =\theta /2$ in Fig. 25(b)). The resulting outflow velocity from the X-line is given by the hybrid Alfvén speed,

39 $$ V_{A, {\mathrm{asym}}}= \sqrt{ \frac{B_{1}+B_{2}}{4\pi m_{i}\left (\frac{n_{1}}{B_{1}}+\frac{n_{2}}{B_{2}}\right )}} \sin{\frac{\theta}{2}}. $$

This equation agrees with Eq. (21).

Second, we calculate the component of the diamagnetic velocity along the outflow ($x$-) direction,

40 $$ \left < V^{*}_{x}\right >=-\left < \frac{cB_{y}\partial _{z} P}{enB^{2}}\right >, $$

where $\partial _{z} P$ is the derivative of the total pressure (electron plus ion) in the direction normal to the current layer. The angle bracket $\left < \right >$ indicates the spatial average across the current sheet of thickness $\delta $.

The stability condition (i.e., $\left < V^{*}_{x}\right > > V_{A}$) leads to

41 $$ \lvert \beta _{1}-\beta _{2}\rvert > \frac{2\delta}{d_{i}} \tan{ \frac{\theta}{2}} $$

which was first derived in Swisdak et al. (2010). Here $\beta _{1}$ and $\beta _{2}$ are the plasma betas on two sides.

Simulations suggest that the scale factor $\delta $ is $\approx d_{i}$ when the guide field is small, but $\approx \rho _{s}$, the sound Larmor radius, is in the opposite limit. However, the approximations made in deriving Eq. (41) mean that the pre-factor on the right-hand side is likely most accurately described as “of order unity” and so exactness is not expected. Immediate consequences of Eq. (41) include: (1) Anti-parallel reconnection ($\theta = \pi $) is never subject to diamagnetic stabilization and (2) Stabilization is likely when the upstream fields nearly align (i.e., for $\theta $ small).

##### Observational Evidence

**Fig. 26:**
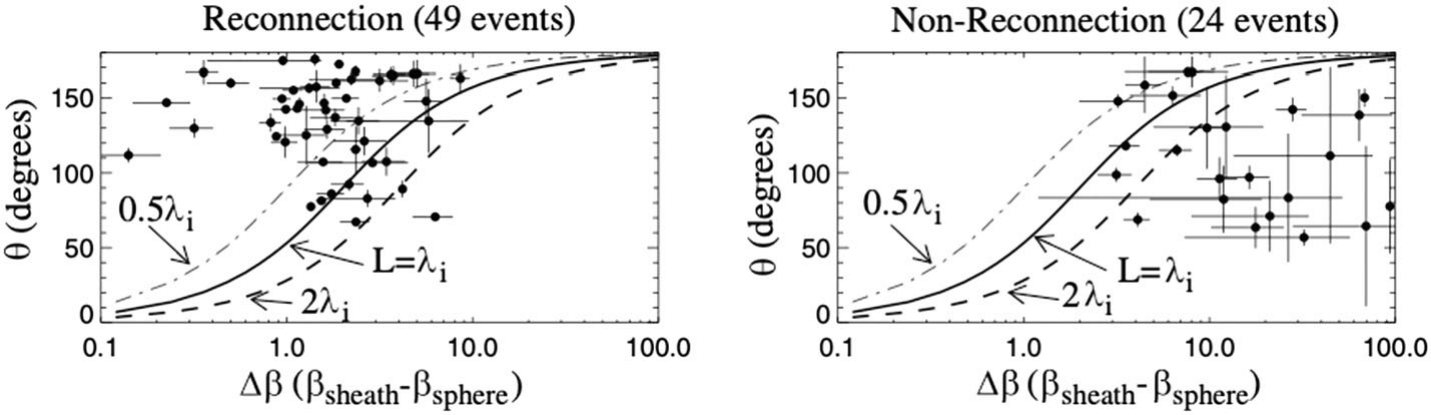
Results of statistical survey of reconnection (left) and non-reconnection (right) events. Scatter plot of magnetic shear versus $\Delta \beta $ at the magnetopause. Reprinted from Phan et al. (2013a), with the permission of Wiley

The condition expressed in Eq. (41) has been tested in a variety of locations, e.g., Earth’s magnetopause (Phan et al. 2013a), the solar wind (Phan et al. 2010), and the magnetospheres of Mercury (DiBraccio et al. 2013), Jupiter (Desroche et al. 2012), and Saturn (Masters et al. 2012), with reasonable success. Figure 26 shows a representative example from Phan et al. (2013a) examining reconnection at the magnetopause. Both panels show the $\Delta \beta -\theta $ plane. The various curves divide the plane according to Eq. (41) with the upper/leftmost region where diamagnetic suppression should not occur and the lower/rightmost region where it should. The left panel shows current sheet crossings for which reconnection was detected, while the right panel plots crossings for which no reconnection signals were observed. To a large degree, the events are properly segregated by the diamagnetic suppression condition. However, it is important to recognize that non-reconnecting current sheets are, in general, observed even when diamagnetic suppression is not expected to operate, as other factors (e.g., current sheet thickness) may prevent reconnection onset.

#### Sheared Flow Suppression

**Fig. 27:**
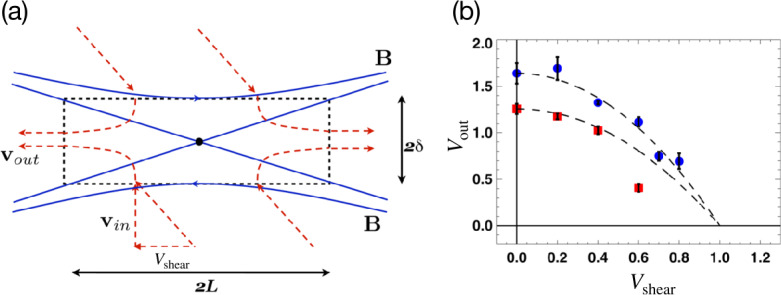
(a) In-plane shear flow across the reconnection current sheet (reprinted from Cassak and Otto 2011, with the permission of AIP Publishing). (b) The outflow speed as a function of shear flow magnitude $V_{\mathrm{shear}}$ during symmetric reconnection. Reprinted from Cassak (2011), with the permission of AIP Publishing

Reconnection can also be suppressed by a background in-plane flow shear across the reconnection current sheet, a scenario in which there are different bulk flow speeds on either side of the reconnection site, as illustrated in Fig. 27(a)). The suppression criterion is qualitatively similar to that of the diamagnetic case (Mitchell Jr. and Kan 1978; Chen and Morrison 1990; La Belle-Hamer et al. 1995; Cassak and Otto 2011). The presence of a flow shear $V_{{\mathrm{shear}}}$ opposes the development of reconnection outflows. If the shear flow velocity $V_{{\mathrm{shear}}} = (V_{x1} - V_{x2})/2$, where $V_{x1}$ and $V_{x2}$ are the bulk flow speed on either side of the reconnection site, is larger than the Alfvén speed,

42 $$ V_{\mathrm{shear}} > V_{A}, $$

the reconnection outflow can not develop. Such outflow reduction was demonstrated using two-fluid simulations in Fig. 27(b). A similar conclusion is extended to relativistic magnetic reconnection (Peery et al. 2024), but the critical velocity is set by the relativistic Alfvén speed, like that discussed in Sect. 3.11.

Since magnetopause reconnection, where the upstream plasma can have a bulk flow because the magnetosheath plasma moves due to the solar wind, is asymmetric, we will discuss how flow shear impacts asymmetric reconnection. If asymmetric reconnection occurs in a region where there is a bulk flow $V_{x}$ in the $x$-direction (along or against the reconnecting magnetic field) that is different on either side of the diffusion region, reconnection can slow down. If the flow shear $V_{{\mathrm{shear}}} = (V_{x1} - V_{x2})/2$ is large enough, it can fully suppress reconnection (Doss et al. 2015), just as in the symmetric reconnection case. The reconnection electric field $E_{R,{\mathrm{asym},\mathrm{ shear}}}$ was shown to scale as

43 $$ E_{R,{\mathrm{asym},\mathrm{ shear}}}\sim E_{R,{\mathrm{asym}}} \left [1- \frac{V_{{\mathrm{shear}}}^{2}}{V_{A,{\mathrm{asym}}}^{2}} \frac{4 n_{1} B_{x2} n_{2} B_{x1}}{(n_{1} B_{x2} + n_{2} B_{x1})^{2}} \right ], $$

where $V_{A,{\mathrm{asym}}}$ and $E_{R,{\mathrm{asym}}}$ are defined in Eq. (21)-(22). The critical flow shear at which reconnection is suppressed, when $E_{R,{\mathrm{sym},\mathrm{ shear}}}$ goes to zero, is

44 $$ V_{{\mathrm{shear},\mathrm{crit}}} \sim V_{A, {\mathrm{asym}}} \frac{n_{1} B_{x2} + n_{2} B_{x1}}{2 \sqrt{n_{1} B_{x2} n_{2} B_{x1}}}. $$

In the symmetric limit, the critical flow shear is simply $V_{A}$, as has been well known (Mitchell Jr. and Kan 1978; Chen and Morrison 1990; La Belle-Hamer et al. 1995; Cassak and Otto 2011). Interestingly, for asymmetric reconnection, the critical flow shear is faster. This implies that it is more difficult to suppress asymmetric reconnection by flow shear than symmetric reconnection (Doss et al. 2015). It follows that magnetopause reconnection is not expected to be suppressed by flow shear (Doss et al. 2015).

##### Observational Evidence

During radial IMF conditions, the magnetosheath flow and the direction of reconnection jets become roughly aligned at the magnetopause flanks. Toledo-Redondo et al. (2021) took advantage of MMS-Cluster conjunction to investigate the mesoscale of magnetic reconnection along the magnetopause. The MMS fleet was located near the subsolar region, while the Cluster fleet was located in the dusk flank ($\mathrm{X}_{GSE}\sim 0$). Based on seven simultaneous crossings (magnetopause current sheet observation within 5 minutes at the two locations), the expected reconnection electric field was $E_{R,\mathrm{asym},\mathrm{shear}}/E_{R,\mathrm{asym}}= 0.71\text{-}0.98$ in the flank, based on Eq. (43), and thus the effect was negligible for the seven crossings near the subsolar region. While these observations indicate that the shear flow suppression mechanism may have some impact in regulating the global coupling of the magnetosphere to the solar wind during radial IMF conditions, more observations are needed to quantify this impact.

### Reconnection Driven by Converging Flows

While flows that oppose the development of outflows can suppress reconnection, external flows that converge into the current sheet can, in contrast, drive the reconnection process.

**Fig. 28:**
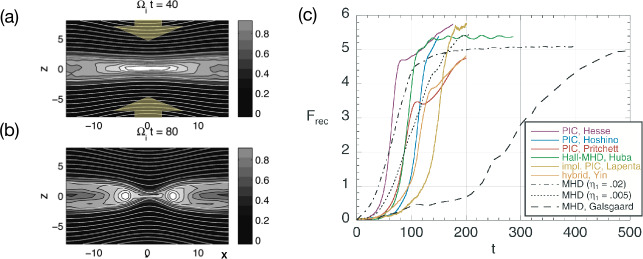
*Newton Challenge*. (a)-(b) Reconnection driven from the top and bottom inflow boundaries within a PIC simulation. Adapted from Pritchett (2005). (c) The evolution of the reconnected magnetic flux. PIC, Hall, and hybrid simulations show similar reconnection rates (given by the slope of each curve), while resistive MHD simulations show lower reconnection rates. Reprinted from Birn et al. (2005), with the permission of Wiley

To compare the results of driven reconnection in various different types of simulations, a series of studies called the Newton Challenge (Birn et al. 2005; Pritchett 2005; Huba 2006; see Fig. 28) was conducted. Similar to the GEM Reconnection Challenge (Birn et al. 2001), these authors used two-dimensional resistive MHD (with a localized resistivity), Hall MHD, full PIC, and hybrid simulations. Boundary inflows were imposed on both the top and the bottom boundaries with the functional form $V_{\mathrm{in}}=2a\omega \text{tanh}(\omega t)/\text{cosh}^{2}(\omega t)$ with $a=2d_{i}$ and $\omega =0.05\Omega _{ci}$, giving the maximum inflow speed $0.08V_{A}$, where $V_{A}=B_{x0}/\sqrt{4\pi n_{0} m_{i}}$ with $B_{x0}$ being the asymptotic reconnecting field and $n_{0}$ being the initial peak density in the current sheet. These inflows drive the boundary electric field $E_{y}=B_{x}V_{\mathrm{in}}/c$ out of the reconnection plane, where the maximum electric field reaches $0.1B_{x0}V_{A}/c$. Note that the reconnecting component $B_{x}$ at the boundary increases due to $V_{\mathrm{in}}$, with the maximum value around 1.1 to 1.2 times larger than the initial asymptotic value $B_{x0}$. Such a “pileup” of the upstream magnetic flux may compensate for any local reduction in the reconnection rate due to a weak current sheet dissipation (Dorelli 2019), resolving the debate of whether magnetopause reconnection rates are controlled by the solar wind driving or local reconnection physics (Borovsky et al. 2008; Lopez 2016).

The PIC simulation results of the Newton Challenge by Pritchett (2005) show that the reconnection electric field $E_{R}$ at the X-line increases with time, reaching a maximum of ${\sim} 0.12 B_{x0}V_{A}/c$, slightly larger than the maximum of the boundary $E_{y}$, after which $E_{R}$ decreases to values less than $0.05 B_{x0}V_{A}/c$. In the later stage, reconnection reaches a quasi-steady state, during which the magnetic field geometry shows an almost equilibrium state with two large magnetic islands. In all of the physical models, the final state reaches a similar equilibrium, even though the reconnection rate (the reconnection electric field) in the resistive MHD simulations in the earlier fast stage is slightly smaller than that in simulations with Hall physics.

Although the final magnetic configurations are the same in all the simulation methods, the “two-stage” evolution of reconnection (the fast phase followed by the slow phase) is observed only in PIC and hybrid simulations, where particle kinetics is included. Pritchett (2005) explains that the following slow phase in the Newton Challenge appears after the outflows reach the periodic boundaries, and at that time, the system does not reach equilibrium with a large magnetic island. In contrast with kinetic simulations, Hall MHD (Huba 2006) and resistive MHD (Birn et al. 2005) simulations do not show the two-step evolution, and the fast reconnection phase continues until the system reaches the equilibrium with a large magnetic island. These results suggest that the kinetics of ions and electrons play a role in the late-stage evolution of driven reconnection.

While the Newton Challenge was primarily a study of 2D-driven reconnection, 3D-driven reconnection was also considered. Sullivan and Rogers (2008) used Hall MHD simulations with external inflows similar to those in the Newton Challenge to compare the results in 2D and 3D simulations. The external inflows in 3D cases are not uniform in the $y$ (electric current) direction but are localized around $y=0$ in the simulation box. In 2D simulations, they observed the reconnection electric field scaled as predicted, $E_{R}\sim (\delta /L) V_{Am} B_{m}/c$, where $V_{Am}$ and $B_{m}$ are the Alfvén speed and the magnetic field at the edge of the diffusion region, and the aspect ratio of the diffusion region $\delta /L$ is 0.1-0.2. In contrast, in 3D runs, the reconnection rate is a factor of 2 larger than the prediction of $(\delta /L) V_{Am} B_{m}/c$, which is attributed to the fact that the diffusion region is localized in the $y$ direction and not uniformly distributed as in Pritchett (2005).

#### Reconnection within Vortices, Turbulence and Shocks

MMS has observed evidence of driven reconnection in the flanks of the Earth’s magnetopause. During northward IMF reconnection occurs in the high-latitude regions, which transfer the accumulated magnetic flux to the low-latitude magnetopause, forming the so-called “low-latitude boundary layer” (LLBL), in which most of the plasma originates from the magnetosheath. In the flank side of the LLBL, strong velocity shear is unstable to the Kelvin-Helmholtz (KH) instability. The super-Alfvénic shear flows associated with this instability can drive reconnection within these vortices (see e.g. Hwang et al. 2023, this collection, and references therein). MMS detected such KH-driven reconnection (Eriksson et al. 2016a; Li et al. 2016; Nakamura et al. 2017b, 2018; Hwang et al. 2020; Kieokaew et al. 2020; Hwang et al. 2021).

**Fig. 29:**
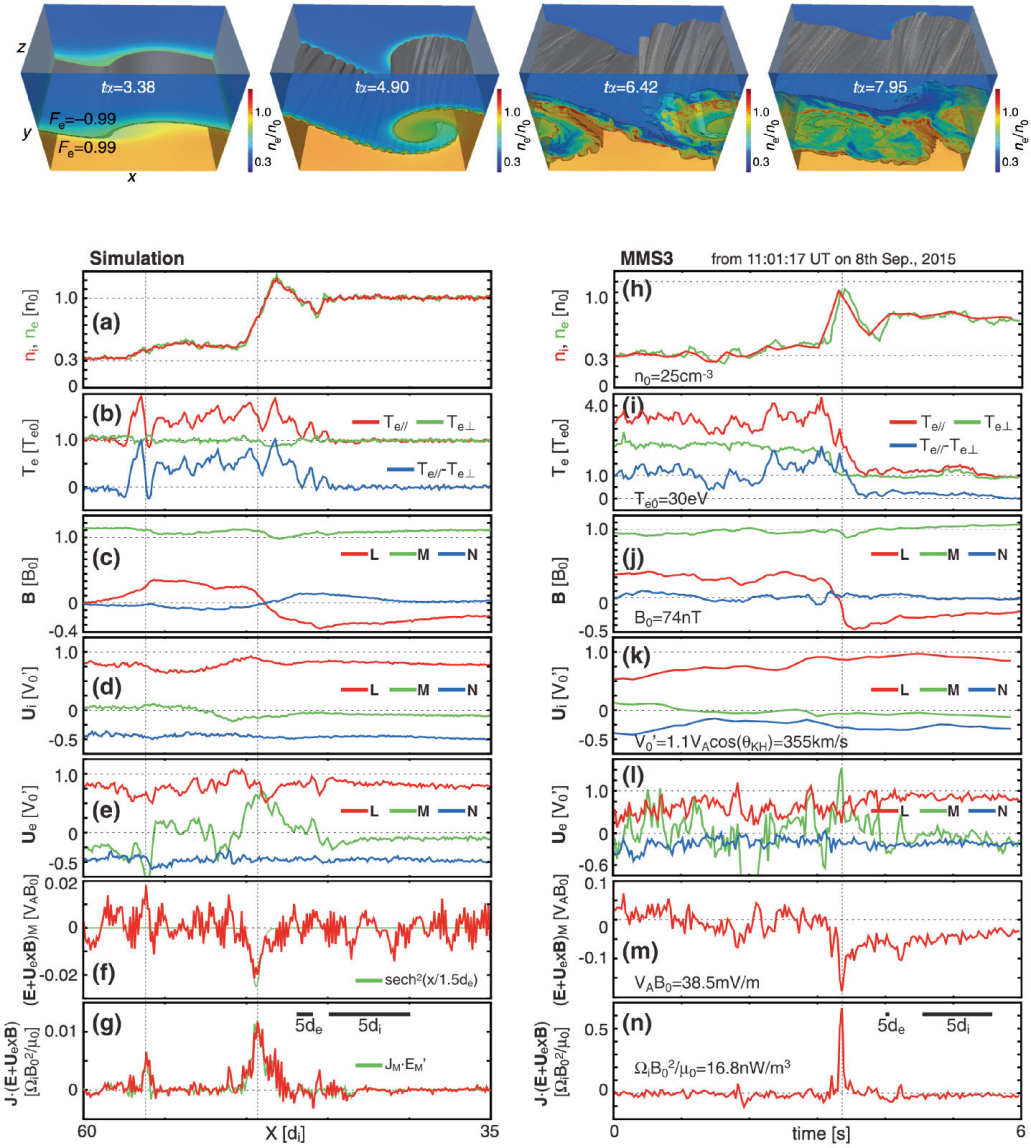
Top panels show the time evolution of Kelvin-Helmholtz vortex-induced reconnection (VIR) in a 3D PIC simulation (Nakamura et al. 2017a). The rest of the panels show its comparison with MM3 data obtained on September 8, 2015. Especially, panels (f) and (m) compare the non-ideal electric field in the M direction, which shows $0.5V_{AL}B_{L}$ and $1V_{AL}B_{L}$, respectively, due to reconnection driven by the vortex flow. Reprinted from Nakamura et al. (2017b), with the permission of Wiley

Nakamura et al. (2017b) presented 3D PIC simulations of a KH-driven reconnection event observed by MMS on 8 September 2015, as illustrated in Fig. 29. The right panels indicate that MMS3 crossed a current sheet in the reconnection region from the magnetospheric side to the magnetosheath side, during which it detected a large non-ideal electric field $E'_{M}\equiv ({\mathbf{E}}+{\mathbf{V}}_{e}\times {\mathbf{B}}/c)_{M}=-7\text{ mV/m}$; although this value corresponds to ${\sim} 0.2 V_{A}B_{x0}$, it is ${\sim} 1V_{AL}B_{L}$ if one normalizes it to the $L$ component of the local magnetic field 2$B_{L}\sim 35$ nT × the Alfvén speed $V_{AL}$ based on $B_{L}$ and the local density $17 \text{ cm}^{-3}$. This $\vert E'_{M} \vert \sim V_{AL}B_{L}$ is 10 times larger than a typical standard laminar reconnection value of $0.1V_{AL}B_{L}$, perhaps due to the fact that reconnection is driven by the strong flows generated by the KH instability. The observational data are consistent with the 3D PIC simulation in the left panels, where the peak of $\vert E'_{M} \vert $ (panel (f)) is $0.5 V_{AL}B_{L}$, which is also 5 times larger than the standard reconnection rate.

Driven reconnection can also occur in turbulent environments. The strong flows therein can force reconnection to occur at a variety of rates. Haggerty et al. (2017) performed 2D PIC simulations of turbulent reconnection and observed normalized reconnection rates distributed between 0 to 0.5, suggesting that reconnection rates are not limited to the order of 0.1. Bessho et al. (2020, 2022) demonstrated using 2D PIC simulations that reconnection in turbulence associated with Earth’s bow shock is strongly driven by super Alfvénic flows, and normalized reconnection rates in both electron-only reconnection and ion-coupled reconnection can be of the order of unity.

### Turbulent 3D Reconnection

While kinetic-scale reconnection (either “electron-only” or ion-coupled) within turbulent plasmas can be affected by turbulence driving, it has been proposed that the dissipation mechanism of reconnection itself may be affected by turbulence or instabilities within the diffusion region. Turbulent reconnection operates in large, 3D systems, where the additional degree of freedom introduces various types of instabilities (e.g. Daughton et al. 2011), leading to turbulence. Unlike laminar reconnection, it was proposed that turbulence may produce “anomalous resistivity” (e.g., Higashimori et al. 2013) or “anomalous viscosity” (e.g., Che et al. 2011a; Price et al. 2016), modifying the diffusion region physics that breaks the MHD frozen-in condition. Other competing ideas also exist, including coupling to the Goldreich-Sridhar-like turbulence spectrum (Lazarian and Vishniac 1999), field-line super-diffusion (Eyink et al. 2011), and fast field-line separation (Boozer 2012).

**Fig. 30:**
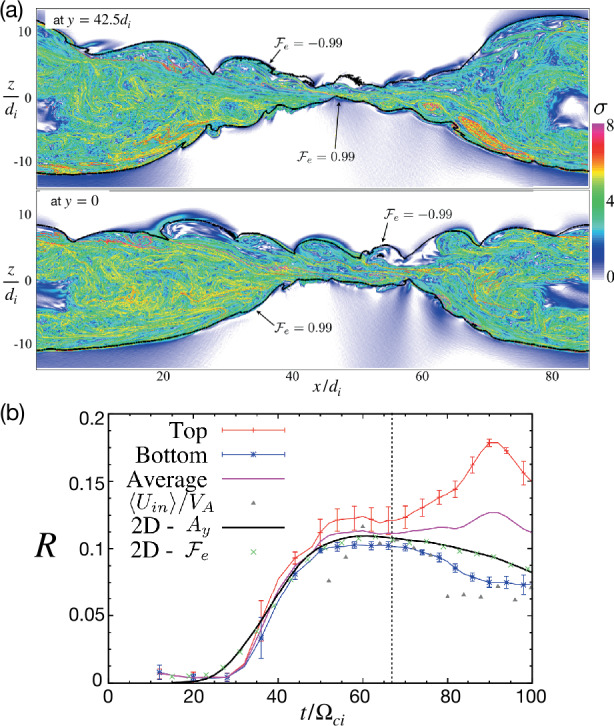
Panel (a) shows the field line exponentiation factor $\sigma $ at two $y$-locations within a 3D PIC simulation. The solid black lines mark the boundaries of the electron mixing fraction $\vert \mathcal{F}_{e}\vert =0.99$. Panel (b) shows the reconnection rate computed based on the top (red), bottom (blue), and average (purple) magnetic fluxes and using $\vert \mathcal{F}_{e}\vert =0.99$. Grey triangles are the inflow rates applied to the bottom region. The black curve is the corresponding 2D reconnection rate measured from the flux function $A_{y}$, while the green crosses are the 2D rate obtained from the same electron mixing approach. Reprinted from Daughton et al. (2014), with the permission of AIP Publishing

Figure 30 shows an example of 3D magnetic reconnection in a large PIC simulation. The entire reconnection layer becomes turbulent because of self-driven instabilities within the current sheet. Unlike in 2D models, an in-plane flux function does not exist that allows a straightforward calculation of the reconnection rate. Instead, Daughton et al. (2014) devised an approach based on the electron mixing across the separatrix in full 3D systems. The measured reconnection rate is shown in Fig. 30(b), and interestingly, the 3D reconnection rate appears to be similar to its 2D laminar counterpart (also in Le et al. 2018). This 3D rate may still be bounded by the same geometrical constraints discussed in Sect. 3.1.2 if the force balance is taken to work in an average sense. In addition, a broad turbulent reconnection layer is often dominated by a few active diffusion regions at the kinetic scale where the dissipation mechanism may be similar to that in Sect. 2. A thorough investigation is required to validate these assertions. To date, it remains challenging to model turbulent reconnection rate from first principles; more discussion on this topic can be found in Stawarz et al. (2024, this collection), Graham et al. (2025, this collection) and Guo et al. (2024).

#### Averaged 3D Ohm’s Law

Other than those kinetic terms discussed in Sect. 2.1, an alternative view holds that fluctuating electric fields, generated by kinetic instabilities such as Buneman modes or lower-hybrid drift effects, can effectively scatter electrons in the electron diffusion region and consequently lead to effective resistance to the reconnection electric field. The effects of such fluctuations are captured by time- or spatial averaging of the microscopic electron momentum equation (Eq. (1)):

45 $$ \begin{aligned} e\left < n_{e}\right >\left < {\mathbf{E}}\right > =- \frac{e}{c}\left < n_{e}\right >\left < {\mathbf{V}}_{e}\right >\times \left < {\mathbf{B}}\right > -\nabla \cdot \left < {\mathbf{P}}_{e}\right > -m_{e} \nabla \cdot (\left < n_{e} {\mathbf{V}}_{e}\right >\left < {\mathbf{V}}_{e} \right >) -m_{e} \frac{\partial \left < n_{e} {\mathbf{V}}_{e}\right >}{\partial t} \\ -e\left < \delta n_{e} \delta {\mathbf{E}}\right > -\frac{e}{c}\left < \delta (n_{e} {\mathbf{V}}_{e})\times \delta {\mathbf{B}}\right > +m_{e} \nabla \cdot \left < \delta (n_{e}{\mathbf{V}}_{e})\delta {\mathbf{V}}_{e} \right >. \end{aligned} $$

Here, $\left < \right >$ denotes spatial averages and the fluctuation of a quantity ${\mathbf{Q}}$ is $\delta \mathbf{Q} \equiv {\mathbf{Q}}-\left < {\mathbf{Q}}\right >$; a similar equation can be obtained for time averaging, with a different form of the time derivative of the electron momentum density (Le et al. 2018). The last three terms on the RHS of Eq. (45) are often referred to as anomalous drag, anomalous momentum transport, and anomalous viscosity, respectively (e.g., Büchner et al. 1998; Che et al. 2011a,b; Price et al. 2016; Le et al. 2018). It should be noted that Eq. (45) does not contain any new or additional information: all information is already included in the microscopic description (Eq. (1)), while Equation (45) is obtained by spatial averaging of this microscopic equation and hence contains less information.

Translationally invariant models demonstrate, without exception, that non-gyrotropic pressure tensor effects dominate at the X-line for symmetric configurations (or, more generally, at the flow stagnation point) (e.g., Pritchett 2001; Schmitz and Grauer 2006). Three-dimensional models of collisionless reconnection can show, however, when averaged, significant contributions of the anomalous terms and the presence of substantial fluctuations at the X point (e.g., Büchner et al. 1998; Che et al. 2011a,b; Fujimoto and Sydora 2012; Price et al. 2016; Muñoz and Büchner 2016). However, some local analyses continued to show the dominance of non-gyrotropic pressure terms (Hesse et al. 2005; Liu et al. 2024), and the magnitude of these anomalous terms are sensitive to how one averages the Ohms’ law (Le et al. 2018). A recent, very large simulation demonstrated the near-absence of significant fluctuations at the X-line if effects of periodic boundaries can be excluded (Liu et al. 2018c).

Prior to the Magnetospheric Multiscale mission, these two theories (i.e., anomalous dissipation versus non-gyrotropic electron pressure) were competing, and MMS had, as a key goal, to determine which of these theories was matched by reality. Beginning with the first key observation of an electron diffusion region at the magnetopause (Burch et al. 2016b), observations have shown remarkably quiescent electron diffusion regions, whether they are asymmetric with (Burch and Phan 2016) or without a guide field (Burch et al. 2016b), or whether they are in the tail’s plasma sheet (Torbert et al. 2018). While it has been difficult to measure electron pressure tensor effects directly, there has been some indication that these are indeed important (Genestreti et al. 2018c), and a recent observation even shows that the analytic prediction of Hesse et al. (1999, 2011) provides a reasonable match to the observed reconnection electric field (Nakamura et al. 2019). Furthermore, a tailored, translationally invariant, numerical simulation (Nakamura et al. 2018) provides an exceptionally good match between observations and model results. While observations around the outflow region show significant fluctuations and turbulent effects (Ergun et al. 2016, 2018; Burch et al. 2018), there is rapidly increasing evidence that the central electron diffusion region is indeed relatively quiescent and properly described by the quasi-viscous, electron nongyrotropy-based model (Hesse et al. 1999). Therefore, it appears that MMS has accomplished its primary objective: to determine the physics behind the electron diffusion region (Torbert et al. 2018).

### Relativistic Reconnection

In plasmas near compact astrophysical objects, such as neutron stars and black holes, the magnetic field strength is extremely strong (e.g., Uzdensky 2011; Ripperda et al. 2020 and references therein), and the plasma flow speed can become relativistic. Under this condition, assuming an anti-parallel magnetic geometry, the relevant force balance equation becomes

46 $$ \frac{({\mathbf{B}}\cdot \nabla ){\mathbf{B}}}{4\pi}\simeq n'm_{i}({\mathbf{U}} \cdot \nabla ){\mathbf{U}}, $$

where ${\mathbf{U}}=\Gamma {\mathbf{V}}$, $\Gamma \equiv [1-(V/c)^{2}]^{-1/2}$ is the Lorentz factor, and $n'$ is the plasma proper density. The resulting outflow speed (in the $x$-direction) is the relativistic Alfvén speed (Liu et al. 2017),

47 $$ V_{\mathrm{out}}\simeq V_{Ax}=c\sqrt{\frac{\sigma _{R}}{1+\sigma _{R}}}, $$

which can approach the speed of light $c$ when the magnetization parameter $\sigma _{R}= B_{R}^{2}/4\pi n' mc^{2} \gg 1$. With an external guide field $B_{g}$, the Alfvénic outflow speed becomes

48 $$ V_{\mathrm{out}}\simeq V_{Ax}=c\sqrt{ \frac{\sigma _{R}}{1+\sigma _{R}+\sigma _{g}}}, $$

where $\sigma _{g}= B_{g}^{2}/4\pi n' mc^{2}$. This expression can be formally derived after considering the additional momentum carried by the outflowing Poynting vector $S_{x}=-E_{z}B_{y}/4\pi $ (that is not included in Eq. (46), but considered in Peery et al. (2024)), where the motional electric field $E_{z}=-V_{\mathrm{out}} B_{g}/c$ is associated with the convection of the guide field. It is interesting to note that the guide field can significantly slow down the outflow speed, unlike in the non-relativistic case. To comprehend this fact in another way, we see that with a guide field the total Alfvén speed (i.e., Eq. (47) with $\sigma _{R}$ replaced by $\sigma _{R}+\sigma _{g}$) is still limited by the speed of light $c$ due to the special relativity, and Eq. (48) is the projection of this total Alfvén velocity along the magnetic field to the outflow direction (Melzani et al. 2014; Liu et al. 2015a), thus its magnitude is expected to be lower than $c$.

Most theoretical studies of relativistic reconnection rates (Blackman and Field 1994; Lyutikov and Uzdensky 2003; Lyubarsky 2005; Liu et al. 2017; Mbarek et al. 2022; Goodbred and Liu 2022) have been performed in the comoving frame of the X-line, where the X-line stays stationary in the 2D reconnection plane. However, observers at different inertial reference frames will disagree on the magnitude of the reconnection electric field and even the magnetic topology within the diffusion region (Hornig and Schindler 1996), because electric fields and magnetic fields can convert into each other in the Lorentz (frame) transformation.. In the absence of a “special” reference frame, especially in a system that lacks symmetry, a covariant (frame-independent) definition of magnetic reconnection becomes desirable. Scientists have started to address this nontrivial issue (Hornig and Schindler 1996; Asenjo and Comisso 2015; Pegoraro 2016).

Relativistic magnetic reconnection has been proposed to explain the superflares observed in the Crab Nebula and argued to cause fast radio bursts (FRBs) from neutron stars and magnetars (Philippov et al. 2019; Mahlmann et al. 2022). Interested readers are referred to the discussion in Guo et al. (2024, this collection).

## Energy Conversion within the Diffusion Region

Aside from changing the large-scale magnetic connectivity/topology, perhaps the most important consequence of magnetic reconnection is converting magnetic energy into plasma kinetic energy and thermal energy. In this section, we collect approaches being used to quantify the energetics and energy conversion processes around the diffusion region, with a particular focus on progress enabled by MMS observations as well as recent advances in simulation capabilities. For the discussion of non-thermal particle accelerations during reconnections, a complimentary review can be found in Oka et al. (2023, this collection).

### Energy Conservation and Energy Fluxes

The second moment of the Vlasov equation gives the energy equation, which in the conservative form (Birn and Hesse 2010) reads

49 $$ \frac{\partial u_{\mathrm{total}}}{\partial t}+\nabla \cdot \left ({\mathbf{S}}+{ \mathbf{H}}+{\mathbf{K}}+{\mathbf{q}}\right )=0. $$

Here, $u_{\mathrm{total}}\equiv \sum _{s}^{i,e} \left (\text{Tr}({\mathbf{P}}_{s})/2+nm_{s} V_{s}^{2}/2\right )+(B^{2}+E^{2})/8\pi $ is the total energy density with $\text{Tr}({\mathbf{P}}_{s})\equiv \sum _{j}^{x,y,z}{\mathbf{P}}_{s,jj}$ being the trace of the pressure tensor, ${\mathbf{S}}\equiv c{\mathbf{E}}\times {\mathbf{B}}/4\pi $ is the Poynting vector, ${\mathbf{H}}\equiv \sum _{s}^{i,e} \left [(1/2)\text{Tr}({\mathbf{P}}_{s}) { \mathbf{V}}_{s}+{\mathbf{P}}_{s}\cdot {\mathbf{V}}_{s} \right ]$ is the enthalpy flux, and ${\mathbf{K}}\equiv \sum _{s}^{i,e}(1/2)nm_{s} V_{s}^{2} {\mathbf{V}}_{s}$ is the bulk-flow kinetic energy flux. ${\mathbf{q}}\equiv \sum _{s}^{i,e} (m_{s}/2)\int\vert{\mathbf{v}}_{s}-{\mathbf{V}}_{s} \vert ^{2}({\mathbf{v}}_{s}- {\mathbf{V}}_{s})f_{s} d^{3}v_{s}$ is the heat flux, where ${\mathbf{v}}_{s}$ is the particle velocity, and $f_{s}$ is the particle distribution function of species “s”.

**Fig. 31:**
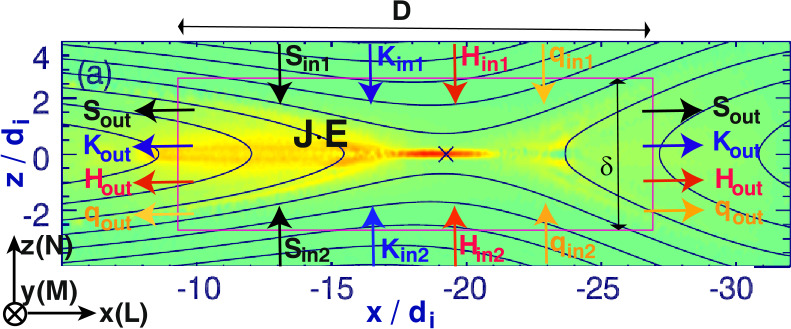
*Schematic of the energy conversion in the reconnection region*, modified from Eastwood et al. (2013). The color represents ${\mathbf{J}}\cdot {\mathbf{E}}$ from a PIC simulation. Reprinted from Lu et al. (2018), with the permission of AIP Publishing

Figure 31 shows schematically the energy fluxes into and out of a reconnection site that is treated as invariant in the y-direction (out-of-plane). Here, for simplicity, the reconnection process is implicitly taken as being in a steady state ($\partial /\partial t\simeq 0$). We now discuss the nature of the energy fluxes in reconnection before turning to look more closely at the problem of energy conversion.

Based on magnetotail observations in the IDR by the Cluster spacecraft, in anti-parallel, symmetric reconnection the outflowing energy flux is dominated by $H_{ix}$, taking up ${\sim} 50 \%$ of the total, followed by $H_{ex}$ (${\sim} 20 \%$) and $K_{ix}$ (${\sim} 10\%$); the outflowing $S_{x}$ (${\sim} 10\%\text{-}20\%$) is comparable to $H_{ex}$ and $K_{ix}$, and even dominates in certain regions in the IDR, where the Hall term dominates (Eastwood et al. 2013). The relative rankings between different forms of energy flux are qualitatively consistent with other subsequent Cluster observations [even when considering the energetics of $\mathrm{O}^{+}$ (Typer et al. 2016)], kinetic simulations (e.g., Birn and Hesse 2010; Lapenta et al. 2020) and laboratory experiments (e.g., Yamada et al. 2016; see also Yamada et al. 2018 and Table 2 in Ji et al. 2023, this collection, for a brief summary of progress in this area).

**Fig. 32:**
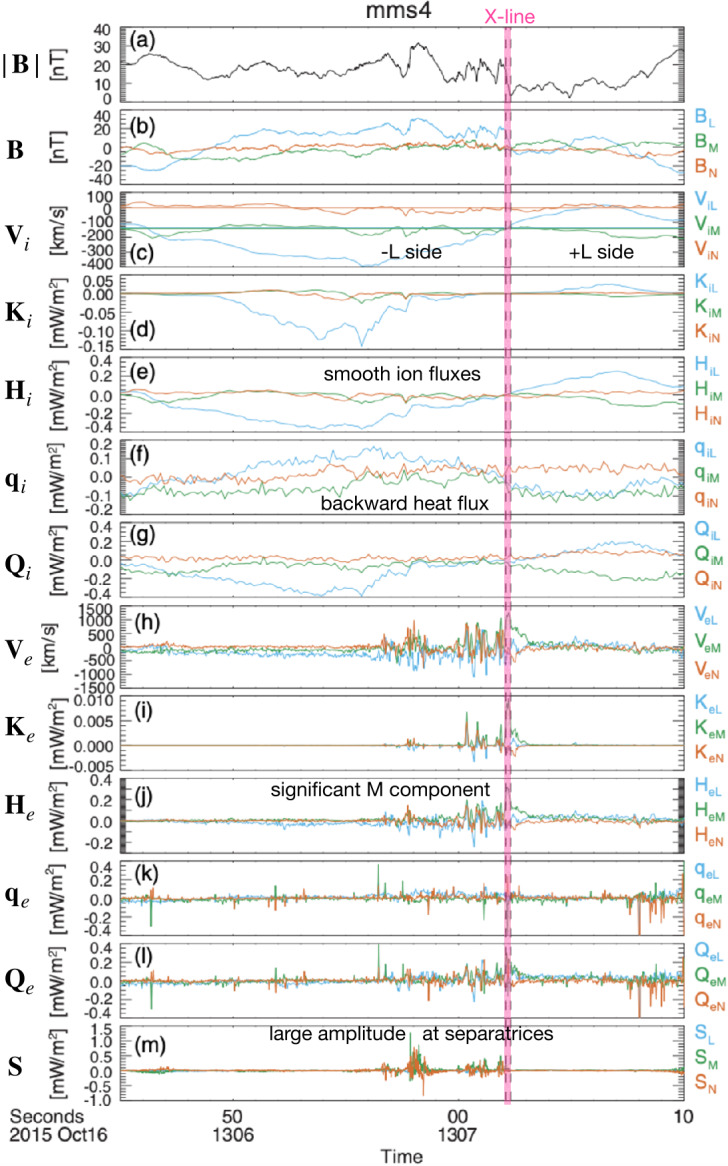
*MMS observations of energy fluxes around an EDR of dayside magnetopause reconnection*, first reported by Burch et al. (2016b). Panels (d, e, f, g) show the kinetic, enthalpy, heat, and total ion flux. Panels (i, j, k, l) show equivalent electron fluxes. MMS reveals that the ion fluxes are smoothly varying, whereas the electron fluxes are structured and variable. The MMS data shows the existence of a significant out-of-plane electron energy flux at the X-line (marked by vertical pink lines), discussed in more detail in the text. Modified from Eastwood et al. (2020)

Prior to MMS, it was not possible to access the dynamics of the EDR with sufficient resolution to determine the detailed properties of the energy fluxes. However, recent efforts have now enabled such analysis around the EDR of asymmetric reconnection at the dayside magnetopause (Eastwood et al. 2020), as shown in Fig. 32. The study also confirms previous ion-scale observations, such as smoothly varying ion energy fluxes dominated by the ion enthalpy flux in the exhausts, and demonstrates the influence of the large-scale asymmetries introduced by the magnetopause, finding, for example, the peak of the total ion energy flux to be displaced towards the magnetospheric side.

In the case of the ions, the heat flux was observed to be directed back towards the X-line, a feature also seen in symmetric reconnection simulations (Lu et al. 2018) and can be explained with non-Maxwellian distributions (Hesse et al. 2018). It should, therefore, be emphasized that the “standard” decomposition of energy flux (which is the relevant parameter for energy transport considerations) in the presence of non-Maxwellian distributions or specifically collections of beams/multiple populations should be interpreted with care (see, e.g. Goldman et al. 2020).

In the case of the electrons, the results from MMS are more surprising. At the EDR, it would be expected to observe an enhanced out-of-plane kinetic energy flux because of the enhanced current density at the X-line. However, the small mass of the electrons renders $K_{e}$ negligible. MMS showed that the combination of electron heating at the EDR together with fast electron motion leads to an out-of-plane electron enthalpy flux density, which is comparable to the ion flux densities in the exhaust (Eastwood et al. 2020). This may have an important impact on the plasma dynamics, particularly in driving electron-scale instabilities out of the plane. The MMS observations raise further questions about the ultimate source and sink of this out-of-plane energy flux at the EDR, and how it varies along the X-line across the magnetopause or in the magnetotail. Answering this requires a more detailed experimental study of both the energy equation (Eq. (49)) as well as the transfer of energy from fields to particles, the latter being controlled by ${\mathbf{J}}\cdot {\mathbf{E}}$ as we now discuss.

### Poynting’s Theorem and ${\mathbf{J}}\cdot {\mathbf{E}}$

To understand the transfer of energy between electromagnetic fields and particles during magnetic reconnection, we can use Poynting’s theorem,

50 $$ \frac{\partial u_{EM}}{\partial t}+\nabla \cdot{\mathbf{S}}=-{\mathbf{J}}\cdot{ \mathbf{E}}, $$

where $u_{EM}=(B^{2}+E^{2})/8\pi $ is the energy density of electromagnetic fields and ${\mathbf{S}} = c({\mathbf{E}}\times{\mathbf{B}})/4\pi $ is the Poynting vector. Since the left-hand side of this equation describes the continuity of the electromagnetic energy, the source term on the right-hand side, ${\mathbf{J}}\cdot {\mathbf{E}}$, will measure the energy conversion from electromagnetic energy to plasma energy. A similar equation can be written for the particles, and the sum of these two equations reduces to Eq. (49), i.e., conservation of total energy. The signature of ${\mathbf{J}}\cdot {\mathbf{E}}$ in anti-parallel, symmetric reconnection is shown in Fig. 31 based on PIC simulation results (Lu et al. 2018). It is most enhanced within the $d_{e}$-scale EDR, but positive values (i.e., energy transfers to the plasma) extend further downstream within the outflow exhaust. ${\mathbf{J}}\cdot {\mathbf{E}}$ can also be decomposed according to the electric field components to assist in understanding the energization mechanisms. The reconnection electric field ($E_{y}$) is along the reconnection X-line. The electric field in the $x-z$ plane, ${\mathbf{E}}_{xz}$, is dominated by the Hall electric fields (${\mathbf{E}}_{ \mathrm{Hall}}={\mathbf{J}}\times {\mathbf{B}}/enc$), which is set up due to the charge separation between the faster-moving electrons and slower ions, with the $z$ component pointing towards the mid-plane and the $x$ component away from the X-line; its effect is to slow down electrons while speeding up ions. It is, therefore, useful to further decompose ${\mathbf{J}}\cdot {\mathbf{E}}$ by considering the current density of each species.

**Fig. 33:**
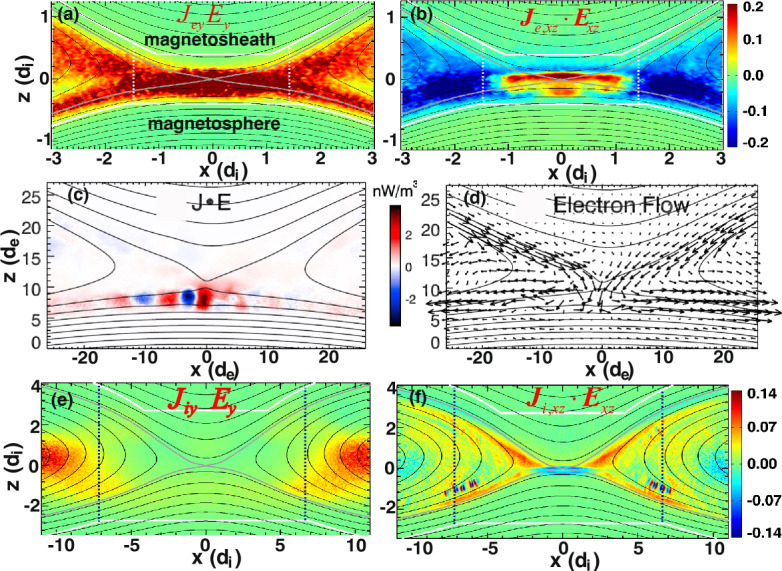
${\mathbf{J}}_{s}\cdot {\mathbf{E}}$ *decomposition by the electric field components in PIC simulations* of asymmetric reconnection with zero guide field. Positive $J_{ey}E_{y}$ dominates the energy conversion to electrons in the EDR (a), and the ${\mathbf{E}}_{xz}$ does negative work outside of EDR (b). (c) In certain parameter regimes, ${\mathbf{J}}_{e}\cdot {\mathbf{E}}$ can exhibit significant oscillations due to oscillating $V_{ez}$ (d). (e)-(f) energy conversion for ions, where the Hall fields dominate while $E_{y}$ has a positive contribution in a broad region. Panels (a), (b), (e), (f) are modified from Wang et al. (2018); (c)-(d) are adapted from Swisdak et al. (2017), with the permission of Wiley

Figure 33 shows such decomposition for asymmetric reconnection in PIC simulations, and we will discuss the electron energization first, then ion energization in the next paragraph. Around the EDR, $J_{ey} E_{y}$ is dominantly positive (Fig. 33(a)), such that electrons gain energy from $E_{y}$ during the meandering motion. ${\mathbf{J}}_{e,xz}\cdot {\mathbf{E}}_{xz}$ is mainly negative, especially further than ${\sim} 1 d_{i}$ downstream of the X-line (Fig. 33(b)). Such features also exist for symmetric reconnection, and ${\mathbf{J}}_{e,xz}\cdot {\mathbf{E}}_{xz}$ within the EDR has a much smaller amplitude than $J_{ey}E_{y}$, as, e.g., shown in Payne et al. (2021). Their study further shows that for a well-developed reconnection layer, a region may develop around the end of the EDR with negative $J_{ey} E_{y}$ (not shown here) and positive $J_{ex} E_{x}$, as the electron flow turns from the $y$ to the $x$ direction and the electrons become re-magnetized. For asymmetric reconnection, because the stagnation point is on the magnetospheric side of the X-line (seen from the electron flow lines in Fig. 33(d)) and the magnetosheath-pointing $E_{z}$ extends to the magnetosheath side of the X-line (e.g., Shay et al. 2016; Chen et al. 2016), a region with positive ${\mathbf{J}}_{e,xz}\cdot {\mathbf{E}}_{xz}$ exists near the X-line that contributes additional electron energy gain (Fig. 33(b)). The ${\mathbf{J}}_{e,xz}\cdot {\mathbf{E}}_{xz}$ profile exhibits fluctuations, and in certain parameter regimes, the fluctuations can be more significant and dominate the total ${\mathbf{J}}\cdot {\mathbf{E}}$ profile (Fig. 5(c), Swisdak et al. 2017). As electrons bounce within the current sheet, they gain a velocity along $x$ by turning around $B_{z}$ and $B_{y}$, so most electrons cannot bounce many times at the same $x$ location to maintain similar densities for populations at positive and negative $v_{z}$, leading to non-zero and fluctuating bulk $V_{ez}$ (Fig. 33(d)) and hence oscillating ${\mathbf{J}}\cdot {\mathbf{E}}$. Adding a guide field, the amplitude of ${\mathbf{J}}_{e,xz}\cdot {\mathbf{E}}_{xz}$ in the central EDR becomes smaller compared to $J_{ey} E_{y}$ (Cassak et al. 2017a; Wang et al. 2018), as electrons have less freedom to bounce across the current sheet.

Within the IDR (but outside the EDR), the electric field ${\mathbf{E}}=-{\mathbf{V}}_{e}\times {\mathbf{B}}/c$, thus the rate of the electron energy gain ${\mathbf{E}}\cdot{\mathbf{J}}_{e}$, vanishes. The rate of the ion energy gain is ${\mathbf{E}}\cdot {\mathbf{J}}_{i} =(-{\mathbf{V}}_{i}\times{\mathbf{B}}/c+{\mathbf{J}}\times{ \mathbf{B}}/nec)\cdot {\mathbf{J}}_{i}={\mathbf{E}}_{\mathrm{Hall}}\cdot {\mathbf{J}}_{i}$. Thus, the Hall electric field dominates the energization of ions overall (Fig. 33(e)-(f)). Since the Hall field is set up due to the ion-electron decoupling, it plays opposite roles in the energization of two species. We may understand the Hall field as a pathway to transfer energies between the two species without energy exchange between fields and particles, as quantified by ${\mathbf{E}}_{\mathrm{Hall}}\cdot {\mathbf{J}}=0$. The Hall electromagnetic fields lead to the diverging Poynting flux streamline patterns around the X-line, which is critical in facilitating fast reconnection (Sect. 3.1.3, Liu et al. 2022). For asymmetric reconnection, ${\mathbf{J}}_{i,xz}\cdot {\mathbf{E}}_{xz}$ is negative in a localized region near the X-line (Fig. 33(f)), and coincides with the positive ${\mathbf{J}}_{e,xz}\cdot {\mathbf{E}}_{xz}$ in the similar region (Fig. 33(b)). $J_{iy}E_{y}$ has a smaller net contribution than ${\mathbf{J}}_{i,xz}\cdot {\mathbf{E}}_{xz}$ when integrating over the entire diffusion region (Wang et al. 2018). However, $J_{iy}E_{y}$ dominates close to the X-line and has positive values in a broad region over $z$ due to $J_{iy}$ from the finite Larmor radius effect of meandering ions near the boundary of the ion current layer (Fig. 33(f)).

Turning to observations more specifically, Genestreti et al. (2018a) demonstrated that ${\mathbf{J}}_{e}\cdot {\mathbf{E}}\sim J_{ey} E_{y}$ in a symmetric reconnection EDR, while ${\mathbf{J}}_{e}\cdot {\mathbf{E}}$ at dayside asymmetric reconnection exhibits significant fluctuations that may be associated with fluctuating upstream conditions (Genestreti et al. 2022) beyond the scope of the simulation discussions here. Genestreti et al. (2018a) and Payne et al. (2020) also used MMS to further evaluate the balance between $\partial u_{EM}/\partial t$ and $-{\mathbf{J}}\cdot {\mathbf{E}}- \nabla \cdot {\mathbf{S}}$ in Poynting’s theorem for magnetopause and magnetotail EDRs, respectively. The time-derivative term $\partial u_{EM}/\partial t$ in the X-line frame was calculated based on $du_{EM}/dt=\partial u_{EM}/\partial t+{\mathbf{V}}_{\mathrm{X}}\cdot \nabla u_{EM}$, where $du_{EM}/dt$ is the temporal evolution in the spacecraft frame, and ${\mathbf{V}}_{\mathrm{X}}$ is the X-line velocity. The results indicate that $\partial u_{EM}/\partial t$ is close to zero near the X-line, but it has more variations away from the X-line. The 2D PIC simulation exhibits an overall consistent pattern (e.g., Payne et al. 2020), while detailed comparisons suggest that events observed by MMS may be at a locally more unsteady state than what is seen in 2D simulations (Genestreti et al. 2018a). A relevant quantity is ${\mathbf{J}}\cdot ({\mathbf{E}}+{\mathbf{V}}_{e}\times {\mathbf{B}}/c)$, that is the energy conversion rate measured in the local bulk electron frame (Zenitani et al. 2011a); this useful quantity is often used to identify EDRs.

### Further Decomposition and $({\mathbf{P}}\cdot \nabla )\cdot {\mathbf{V}}$

We now discuss the evolution of plasma energy, and we treat this by considering two equations that describe the bulk and thermal forms separately. By dotting the momentum equation with ${\mathbf{V}}_{s}$, one can write the governing equation of bulk flow kinetic energy $u_{{\mathrm{bulk}},s}\equiv (1/2)nm_{s} V_{s}^{2}$ in conservative form,

51 $$ \frac{\partial u_{{\mathrm{bulk}},s}}{\partial t}+\nabla \cdot {\mathbf{K}}_{s}={ \mathbf{J}}_{s}\cdot {\mathbf{E}}-{\mathbf{V}}_{s}\cdot (\nabla \cdot {\mathbf{P}}_{s}). $$

Subtracting Eq. (51) from Eq. (49), the equation for the thermal energy $u_{th}\equiv (1/2)\text{Tr}({\mathbf{P}}_{s})$ is obtained,

52 $$ \frac{\partial u_{{\mathrm{th}},s}}{\partial t}+\nabla \cdot {\mathbf{H}}_{s}+ \nabla \cdot {\mathbf{q}}_{s}={\mathbf{V}}_{s}\cdot (\nabla \cdot {\mathbf{P}}_{s}). $$

Note that the sum of Eq. (51) and (52) gives the overall particle energy equation that is the direct counterpart of Poynting’s theorem. Interestingly, from the source terms on the right-hand side of these two equations, we can tell that the ${\mathbf{V}}_{s}\cdot (\nabla \cdot {\mathbf{P}}_{s})$ term re-distributes the energy stored in the bulk and thermal forms.

**Fig. 34:**
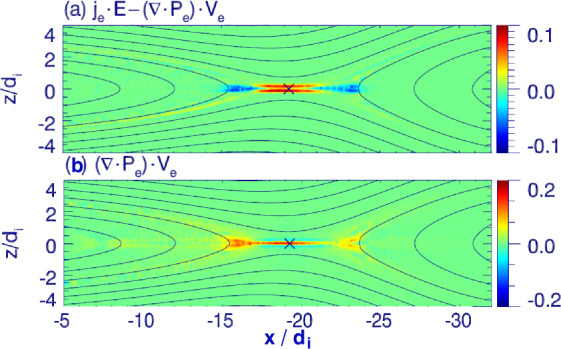
Profiles of the source terms of the electron bulk energy equation ${\mathbf{J}}_{e}\cdot {\mathbf{E}}-{\mathbf{V}}_{e}\cdot (\nabla \cdot {\mathbf{P}}_{e})$ (a) and thermal energy equation ${\mathbf{V}}_{e}\cdot (\nabla \cdot {\mathbf{P}}_{e})$ in PIC. Reprinted from Lu et al. (2018), with the permission of AIP Publishing

Figure 34 shows the source terms on the right-hand side of Eqs. (51) and (52). Electrons gain both significant bulk (Fig. 34a) and thermal energies (Fig. 34b) within the EDR. Around the end of the EDR, the bulk energy gain is negative, and the thermal energy gain is positive, indicating the conversion from bulk to thermal energies (Lu et al. 2018), which from the kinetic perspective is associated with electron re-magnetization through gyro-turning around the reconnected magnetic field (Shuster et al. 2015; Payne et al. 2021). For ions, comparable bulk and thermal energy gains occur throughout the reconnection region (Lu et al. 2018). The source term for the thermal energy gain can be further decomposed in different forms (e.g., Hesse et al. 2018; Lapenta et al. 2020), and it has been demonstrated, using both simulations (Hesse et al. 2018) and MMS observations (Holmes et al. 2021), that the “quasi-viscous” term associated with the off-diagonal components of the pressure tensor has a dominant contribution in describing electron heating from inflow to outflow regions.

There is another form of the equation that is used to quantify the energy conversion between bulk flow kinetic energy and thermal energy of a species $s$. A brief calculation shows that $\nabla \cdot {\mathbf{H}}_{s} = \nabla \cdot (u_{{\mathrm{th}},s} {\mathbf{V}}_{s}) + {\mathbf{V}}_{s} \cdot (\nabla \cdot {\mathbf{P}}_{s}) + ({\mathbf{P}}_{s} \cdot \nabla ) \cdot {\mathbf{V}}_{s}$. Substituting this expression into Eq. (52) gives

53 $$ \frac{\partial u_{{\mathrm{th}},s}}{\partial t}+\nabla \cdot \left (u_{{ \mathrm{th}},s}{\mathbf{V}}_{s} +{\mathbf{q}}_{s} \right )= - ({\mathbf{P}}_{s} \cdot \nabla ) \cdot {\mathbf{V}}_{s}. $$

Similarly, rearranging Eq. (51) gives

54 $$ \frac{\partial u_{{\mathrm{bulk}},s}}{\partial t}+\nabla \cdot ({\mathbf{K}}_{s}+ {\mathbf{V}}_{s}\cdot {\mathbf{P}}_{s})={\mathbf{J}}_{s}\cdot {\mathbf{E}}+ ({\mathbf{P}}_{s} \cdot \nabla ) \cdot {\mathbf{V}}_{s}. $$

In this form, it is readily apparent that $-({\mathbf{P}}_{s} \cdot \nabla ) \cdot {\mathbf{V}}_{s}$ is a source of internal energy. Since the same term appears in the kinetic energy equation with the opposite sign, this term also describes the conversion between bulk kinetic energy and internal energy. This term, with the minus sign, is called “the pressure-strain interaction.” A relevant discussion of this term to reconnection electric field can be found in Sect. 2.3, where this term is explicitly related to the non-gyrotropic plasma pressure within the EDR (Hesse et al. 2018)

The pressure-strain interaction has undergone significant study in the MMS era because MMS is uniquely capable of making reliable *in situ* measurements of it. One special property of the pressure-strain interaction is that if one has a closed (infinite or isolated) system, the volume integral over the whole domain ${\mathrm{V}}$ of Eq. (53) reveals (Yang et al. 2017b, 2022),

55 $$ \frac{dU_{{\mathrm{th}},s}}{dt} = \int _{\mathrm{V}} d^{3}r [- ({\mathbf{P}}_{s} \cdot \nabla ) \cdot {\mathbf{V}}_{s}], $$

where $U_{{\mathrm{th}},s} = \int _{\mathrm{V}}u_{{\mathrm{th}},s} d^{3}r$ is the total thermal energy in the system. Thus, in a collisionless closed system, the volume-integrated pressure-strain interaction is the *only* source of thermal energy. It is important to emphasize, however, that it is not the only source of thermal energy locally at any given position (Song et al. 2020; Du et al. 2020; Barbhuiya et al. 2024); Eq. (53) shows that other terms (the thermal energy flux and the heat flux) can also change the local internal energy. Also, for systems that are not closed (such as any system in space or astrophysical settings), the other fluxes can lead to a non-zero source or sink for internal energy.

The pressure-strain interaction has been further decomposed to isolate the key physics causing the change in internal energy. One decomposition is to write (Yang et al. 2017b,a)

56 $$ -({\mathbf{P}}_{s} \cdot \nabla ) \cdot {\mathbf{V}}_{s} = -P_{s} (\nabla \cdot {\mathbf{V}}_{s}) - \boldsymbol{\Pi}_{s} : {\mathbf{D}}_{s}, $$

where $P_{s} \equiv (1/3) {\mathrm{Tr}}({\mathbf{P}}_{s})$ is the effective (scalar) pressure, $\boldsymbol{\Pi}_{s} = {\mathbf{P}}_{s} - P_{s} {\mathbf{I}}$ is the deviatoric pressure tensor which describes the departure of the pressure tensor from being isotropic, and $D_{s,jk} = (1/2) (\partial V_{s,j}/\partial r_{k} + \partial V_{s,k}/ \partial r_{j}) - (1/3) \delta _{jk} (\nabla \cdot {\mathbf{V}}_{s})$ is the “traceless strain rate tensor” which describes the incompressible portion of the flow. Thus, the first term on the right of Eq. (56) describes heating or cooling via compression or expansion, and the second term on the right describes incompressible deformation of fluid elements (Del Sarto et al. 2016; Yang et al. 2017b; Del Sarto and Pegoraro 2018). The second term can be further decomposed into incompressible deformation due to normal flow and incompressible deformation due to flow shear (Cassak and Barbhuiya 2022). The latter decomposition can be useful for reconnection studies because it isolates the effect of converging flow and flow shear. The pressure-strain interaction has also been written in magnetic field-aligned coordinates (Cassak et al. 2022), which allows one to determine if the compression, deformation, or shear is parallel or perpendicular to the magnetic field. The pressure-strain interaction and its decompositions have been studied in numerical simulations of magnetic reconnection (Sitnov et al. 2018; Du et al. 2018; Song et al. 2020; Fadanelli et al. 2021; Barbhuiya and Cassak 2022) and turbulence (Parashar et al. 2018; Pezzi et al. 2019; Yang et al. 2019; Hellinger et al. 2022) and in MMS observations (Chasapis et al. 2018; Zhong et al. 2019; Bandyopadhyay et al. 2020, 2021; Zhou et al. 2021; Wang et al. 2021).

### Describing Changes to Internal Moments Beyond Internal Energy

Equation (49) and its subsequent decompositions discussed in Sects. 4.1-4.3 follow from the second moment of the Vlasov equation and contain a complete description of the information about energy conversion associated with the number density (the zeroth moment of the distribution function), bulk flow (the first moment), and the thermal energy (the trace of the second moments). However, the distribution function has an infinite number of moments, and the evolution of the other moments is not described by Eq. (49). For systems close to local thermodynamic equilibrium (LTE), *i.e.,* the distribution function is close to being Maxwellian, the other moments are small and their evolution is typically ignored. Any systems of interest for space and astrophysical environments, however, are far from LTE because they are weakly collisional or essentially collisionless. For such systems, it has been unclear how to quantify changes to the higher-order internal moments beyond density, bulk flow, and temperature. The wealth of particle distribution data from MMS, in particular, is now bringing these questions to the fore.

Recently, an approach to quantify changes associated with higher-order internal moments was suggested (Cassak et al. 2023; Barbhuiya et al. 2024). The key quantity is the so-called relative entropy density $s_{s,{\mathrm{rel}}}$, given by

57 $$ s_{s,{\mathrm{rel}}} = -k_{B} \int f_{s} \ln \left ( \frac{f_{s}}{f_{sM}} \right ) d^{3}v_{s}, $$

where the integral is over all of the velocity space. Here, $f_{sM}$ is the “Maxwellianized” distribution associated with the distribution function $f_{s}$, given by a Maxwellian distribution with the number density $n_{s}$, the bulk flow ${\mathbf{V}}_{s}$ and temperature $T_{s} = (1/3) {\mathrm{Tr}}({\mathbf{P}}_{s})/n_{s} k_{B}$ (Grad 1965). This quantity is a measure of how non-Maxwellian a distribution function is, with $s_{s,{\mathrm{rel}}} = 0$ if $f_{s}$ is a Maxwellian distribution and it being negative-definite if $f_{s}$ is anything non-Maxwellian.

Because $s_{s,{\mathrm{rel}}}$ is a measure of how non-Maxwellian a distribution is, its time derivative describes how rapidly the shape of the distribution is changing to become more or less Maxwellian (Cassak et al. 2023). In particular, if $(d/dt) (s_{s,{\mathrm{rel}}}/n_{s}) > 0$, then $f_{s}$ is becoming more Maxwellian in the comoving (Lagrangian) reference frame, while $(d/dt) (s_{s,{\mathrm{rel}}}/n_{s}) < 0$ implies $f_{s}$ is becoming less Maxwellian. Dividing by $n_{s}$ to give the relative entropy per particle is done to not include compression, which is described in the energy equation. It was argued (Cassak et al. 2023) that scaling $(d/dt) (s_{s,{\mathrm{rel}}}/n_{s})$ by the temperature gives an effective energy per particle associated with changes to any (and all) of the higher order moments, called the change of relative energy per particle $d{\mathcal{E}}_{s,{\mathrm{rel}}}$ and given by

58 $$ \frac{d{\mathcal{E}}_{s,{\mathrm{rel}}}}{dt} = T_{s} \frac{d(s_{s,{\mathrm{rel}}}/n_{s})}{dt}. $$

It is important to note that ${\mathcal{E}}_{s,{\mathrm{rel}}}$ is not a form of energy and, therefore, does not appear in the second moment of the Vlasov equation (Eq. (49)), but it does have the same dimensions and therefore is a quantitative measure of the changes to the higher order internal moments of the distribution that can be directly compared to the standard forms of energy.

Understanding the interplay of changes of all of the higher-order internal moments and the lower-order moments is in its infancy. In a single simulation of reconnection using a particle-in-cell code with 25,600 particles per grid cell, it was shown (Cassak et al. 2023) that the relative energy change can locally be important or even dominate the changes of internal. How relative energy and entropy depend on ambient plasma parameters and the time evolution of reconnection remains unknown. Entropy-related quantities have been measured with MMS (Argall et al. 2022), but relative entropy has yet to be measured with MMS.

### Energy Partition between Ions and Electrons

Understanding the energy partition between species is also desirable, particularly for understanding reconnection in settings where data may be incomplete (for example, in remote observations or planetary missions where the experimental payload is not optimized for plasma physics). Most incoming electromagnetic energy is eventually converted to the enthalpy flux at locations away from the X-line, as discussed in Sect. 4.1. In the more general asymmetric reconnection case, the thermal energy gain of each species was modeled as (Wang et al. 2018; Shay et al. 2014)

59 $$ \frac{\Delta U_{{\mathrm{th}},s}}{U_{\mathrm{in}}} =\frac{\gamma}{\gamma -1} \frac{T_{{\mathrm{out}},s}-T_{{\mathrm{in}},s}}{m_{i} V_{A,{\mathrm{asym}}}^{2}}, $$

where $\Delta U_{{\mathrm{th}},s}\equiv \int {\mathbf{J}}_{s}\cdot {\mathbf{E}} d^{3}r$ and $U_{\mathrm{in}}$ is the input field energy available for conversion. $T_{{\mathrm{in}},s}=(n_{1} T_{1,s} B_{2}+n_{2} T_{2,s} B_{1})/(n_{1} B_{2}+n_{2} B_{1})$ represents the inflow temperature, $V_{A,{\mathrm{asym}}}=(B_{1} B_{2}/(4\pi m_{i} )(B_{1}+B_{2})/(n_{1} B_{2}+n_{2} B_{1} ))^{1/2}$ is the hybrid Alfvén speed for asymmetric reconnection (Eq. (21)), and $\gamma =5/3$ is the ratio of specific heats. $T_{{\mathrm{out}},s}\equiv \left < nV_{x,s} T_{s}\right >/\left < nV_{x,s} \right >$ can be regarded as the outflow temperature averaged over the outflow exhaust with a weighting factor of $nV_{x,s}$. The outflow temperature $T_{{\mathrm{out}},s}$ was further approximated to be the temperature averaged using $n$ as the weighting factor. The observations suggest that the heating rate of $(T_{{\mathrm{out}},s}-T_{{\mathrm{in}},s})/(m_{i} V_{A,{\mathrm{asym}}}^{2} )$ is $1.7\%$ for electrons (Phan et al. 2013b) and $13\%$ for ions (Phan et al. 2014), evaluated using the $n$-weighted $T_{{\mathrm{out}},s}$ across exhausts at the far downstream region. PIC simulations show similar results (Shay et al. 2014). A test using PIC indicates that the heating rate based on the $n$-weighted $T_{\mathrm{out}}$ is nearly constant at varying distances from the X-line (Wang et al. 2018). A caveat is that while $T_{\mathrm{out}}$ is insensitive to the approximated forms at distances well away from the EDR, the original $nV_{x}$-weighted $T_{\mathrm{out}}$ should be used around the EDR when using Eq. (59). Close to the EDR, the heating rate is only a few percent while the electron enthalpy flux gain is tens of percent of the incoming Poynting flux as the dominant form of energy conversion. The application of Eq. (59) to a magnetopause reconnection event observed by MMS in between the EDR and IDR boundaries suggests comparable energy partitions between ions and electrons, consistent with the trend predicted by PIC (Wang et al. 2018). We note that the calculation of $nV_{x,s}$-weighted $T_{{\mathrm{out}},s}$ has significant uncertainties, so quantitative values need to be treated with caution.

Particle energization mechanisms provide insight into understanding the scaling laws of heating. For magnetized ions or electrons within outflow exhausts, the particles can be roughly described as moving along field lines at ${\sim} V_{A}$ in the Alfvénic outflow frame. Thus, the superposition of particles from two inflow regions leads to counter-streaming beams in the distribution, so that the effective temperature scales with $V_{A}^{2}$ (e.g., Liu et al. 2011a; Shay et al. 2014). A parallel potential exists in the exhaust, which modulates the beam speeds, and hence modifies the temperature profile and affects the overall $\Delta T_{i}/\Delta T_{e}$ (Haggerty et al. 2015). Such modulations of the ion beam speeds have been observed by MMS (Wang et al. 2019).

**Fig. 35:**
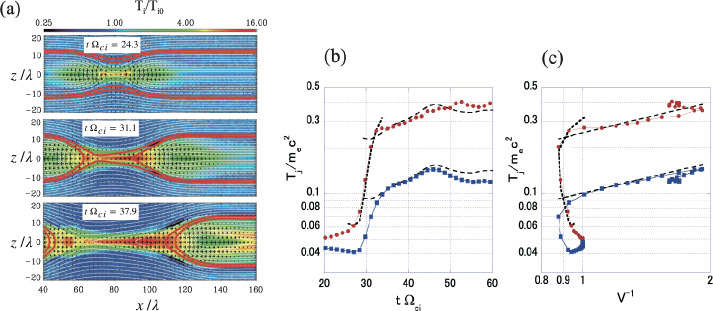
(a) Time evolution of ion temperature in two-dimensional PIC simulation. (b) Time history of ion (red) and electron (blue) temperatures confined in the magnetic flux tube. The dashed lines are the adiabatic relation with $T_{s} {\mathrm{V}}^{2/3}$ being a constant, and the dotted line is obtained using the effective Ohmic heating model of $T_{i}/T_{e} = (m_{i}/m_{e})^{1/4}$ (Eq. (60)). (c) Relationship between the reciprocal of the flux tube volume (${\mathrm{V}}^{-1}$) and the temperatures ($T_{s}$) for ions (red) and electrons (blue). The dashed and dotted lines are the same as those in panel (b)). Adapted from Hoshino (2018), reproduced by permission of the AAS

Inside the diffusion region, the acceleration by $E_{y}$ (reconnection electric field in Fig. 35) during the meandering motion was considered to be the primary energization mechanism. Hoshino (2018) estimated the ratio of the ion-to-electron temperature enhancement $\Delta T_{i}/\Delta T_{e}$ using the effective Ohmic heating rates $E_{y} J_{ys}{\mathrm{V}}_{s}$ of the two species, where $J_{ys}$ and ${\mathrm{V}}_{s}$ are the ion/electron electric current density and the volume of the diffusion region (e.g., Coppi et al. 1966; Coroniti 1985), respectively. This leads to

60 $$ \frac{\Delta \left < T_{i}\right >_{\mathrm{flux}}}{\Delta \left < T_{e}\right >_{\mathrm{flux}}} \simeq \left (\frac{m_{i}}{m_{e}}\right )^{1/4}\left ( \frac{T_{i0}}{T_{e0}}\right )^{1/4}, $$

where $\left < T_{s}\right >_{\mathrm{flux}}$ is the species temperature averaged over the flux tubes and $T_{s0}$ is the far upstream temperature. This scaling is supported by PIC simulations, as shown in Fig. 35. It is also interesting to note that the averaged temperature follows the adiabatic heating law (i.e., $P/n^{5/3}=\mathrm{const}$) during the contraction of reconnected flux tubes (Fig. 35(c)).

## Concluding Remarks and Future Prospects

In this tutorial review article, we have presented the basics of collisionless magnetic reconnection and highlighted some recent progress in understanding the generalized Ohm’s law, the reconnection rate, and the energy conversion around the diffusion region. We also showed supporting evidence from local kinetic simulations and in-situ spacecraft observations, particularly from NASA’s ongoing Magnetospheric Multiscale (MMS) mission, which is capable of performing multi-point measurements on electron-kinetic scale physics within Earth’s magnetosphere.

The discussion of theories in this article focuses mostly on 2D models, originating from the classical Sweet-Parker (Parker 1957; Sweet 1958) and Petschek (Petschek 1964) solutions, where the spatial variation scale along the reconnection X-line is assumed to be much longer than the in-plane spatial scale (i.e., near translational invariance along the out-of-plane direction). The difference of our discussion from these classical models is that we treat the collisionless limit as it is relevant to most applications of reconnection in space plasmas. It is interesting to note that an initially three-dimensional (3D), short reconnection X-line within a uniform current sheet is inclined to spread linearly out of the reconnection plane, making the local geometry two-dimensional (Huba and Rudakov 2002; Shay et al. 2003; Karimabadi et al. 2004; Lapenta et al. 2006; Nakamura et al. 2012; Shepherd and Cassak 2012; Li et al. 2020). One should always be aware that Nature works in three-dimensional space, often accompanied by a high degree of complexity, even if insights from reduced dimensions enable one to extract essential physics. Understanding these 2D limits remains indispensable when seeking to single out the inherently 3D effects that only exist in a 3D system.

In the following, we discuss potential topics critical to the further understanding of magnetic reconnection. From the *local perspective*, a complete theory of the rate of collisionless reconnection, similar to that discussed for standard symmetric reconnection in Sect. 3.1.3, is still missing for most regimes discussed in Sect. 3 and deserves further development. The descriptions proposed thus far are primarily based on the moments of Vlasov equations, only minimally considering kinetic effects. Kinetic features not included here could play important roles and are highlighted in Norgren et al. (2025, this collection).

The study of the three-dimensional nature of reconnection X-lines is also important. For instance, how does the reconnection X-line orient itself within an asymmetric current sheet (Sonnerup 1974; Swisdak and Drake 2007; Hesse et al. 2013; Aunai et al. 2016; Liu et al. 2013, 2018c)? How does an X-line spread (Huba and Rudakov 2003; Shay et al. 2003; Lapenta et al. 2006; Nakamura et al. 2012; Shepherd and Cassak 2012; Jain and Büchner 2017; Liu et al. 2019; Li et al. 2020; Arencibia et al. 2023), and what is its minimal length (Shay et al. 2003; Liu et al. 2019; Huang et al. 2020; Pyakurel et al. 2021)? Over a larger spatial scale, the implication of these “local” 3D X-line properties to global solutions (Trattner et al. 2007) and 3D MHD reconnection theories (Priest et al. 2003; Pontin and Priest 2022; Li et al. 2021b), such as fan-spine reconnection, remains unclear. In terms of future observations, the ESA’s SMILE mission (Raab et al. 2016), NASA’s LEXI telescope (Walsh et al. 2024), and TRACERS (Kletzing 2019) will provide the intriguing possibility of imaging magnetopause dynamics for the first time, providing truly novel experimental data for addressing these questions.

A full 3D system also introduces additional players that are suppressed in two dimensions, including instabilities (Che et al. 2011a; Daughton et al. 2011; Che et al. 2011b; Roytershteyn et al. 2012; Liu et al. 2013), waves (Khotyaintsev et al. 2019; Yoo et al. 2020; Graham et al. 2022; Ng et al. 2023), and turbulence (Ergun et al. 2018; Stawarz et al. 2019), either at MHD or kinetic scales. The impact of these fundamental plasma processes on the reconnection rate and particle energization will continue to be an active direction for research. In particular, while the idea of “anomalous resistivity and transport” is appealing to MHD modeling of magnetic reconnection (Kulsrud 2001; Lin et al. 2021; Jiménez et al. 2022), concrete evidence that links this idea to fast reconnection in collisionless plasmas (Davidson and Gladd 1975; Yoo et al. 2020; Graham et al. 2022; Yoo et al. 2024) remains elusive. Another relevant open question is the existence of turbulent reconnection that has a thick diffusion region (TDR) on the MHD scales (as theorized in, e.g., Lazarian and Vishniac 1999) with well-defined global inflows and outflows. In a separate endeavor, Higashimori et al. (2013) (and more recently Stanish and MacTaggart 2024) demonstrated a nice transition from the Sweet-Parker solution to the Petschek-like solution in their 2D mean-field model, where the turbulence effect can be cranked up to enhance the anomalous resistivity and other non-ideal electric fields in Ohm’s law. The enhanced anomalous resistivity also makes the diffusion region thicker, presumably due to the reduced current density (Lin et al. 2021).

It will be interesting to examine whether such a thick diffusion region is sustainable, not collapsing into kinetic scales, in collisionless plasmas. To address this problem, it would be ideal to have open boundaries in kinetic simulations, avoiding the exaggerated turbulence levels and current sheet broadening caused by the recycling of particles and magnetic structures from small periodic boundary conditions (Liu et al. 2018c). More discussion on the simulation approach of turbulent reconnection can be found in Ji et al. (2022). More discussion on the MMS observations of waves and turbulence associated with reconnection can be found in Stawarz et al. (2024, this collection) and Graham et al. (2025, this collection).

While we only discussed collisionless reconnection in this review, reconnection also occurs in collisional (Daughton et al. 2009; Stanier et al. 2019) and partially ionized plasmas (Zweibel 1989; Zweibel et al. 2011; Murphy and Lukin 2015; Ni et al. 2018; Jara-Almonte et al. 2019; Ni et al. 2020). The study of such reconnection could be important to understand the heating in the lower atmosphere of the Sun (e.g., jetlets in Shibata et al. 2007; Raouafi et al. 2023b) or the production of precipitating energetic electrons in ionospheres (e.g., aurora spirals in Huang et al. 2022). The transition from the collisional to collisionless limits could also be critical in understanding the onset problem of reconnection on the Sun, where the initial current sheet can be collisional and as thick as ${\sim} 10^{6} d_{i}$. The plasmoid instability (Biskamp 1982; Shibata and Tanuma 2001; Bhattacharjee et al. 2009; Loureiro et al. 2007; Pucci and Velli 2014; Comisso et al. 2016) in collisional plasmas may enable a transition into the collisionless regime (Shibata and Tanuma 2001; Daughton et al. 2009; Huang et al. 2017; Stanier et al. 2019; Jara-Almonte and Ji 2021). In contrast, the plasmas in Earth’s magnetotail are nearly collisionless, and the onset study of tail reconnection relevant to substorms is concerned more with the stability of a 2D magnetotail geometry (Schindler 1974; Lembege and Pellat 1982; Hesse and Schindler 2001; Pritchett 2005; Sitnov et al. 2009; Liu et al. 2014a; Bessho and Bhattacharjee 2014), where the collisionless tearing instability can be suppressed by the magnetic field normal to the current sheet (because electrons remain magnetized). The question in this context is under what conditions reconnection onset can be triggered in collisionless plasmas, enabling the energy release of geomagnetic substorms. More discussion on the onset problem can be found in Nakamura et al. (2025, this collection).

From the *global perspective*, it is critical to integrate our understanding of the local reconnection physics into the macroscale phenomena of a given system. The multiscale nature of reconnection makes this process interesting but also challenging for both first-principles numerical simulations and analytical theory. While the theoretical framework in Sect. 3.1 had coupled the mesoscale MHD region upstream of the ion diffusion region (IDR) to the electron diffusion region (EDR) in the steady state, it is assumed that the flux-breaking mechanism within the EDR can “passively” match (presumably by thinning) the reconnection electric field dictated by the outer region. A detailed coupling between the EDR particle kinetics (as discussed in Norgren et al. 2025, this collection) and the IDR solution has not yet been established. On the other hand, it remains unclear how one can couple this locally steady-state solution to the global macroscale in general settings, not to mention the difficulty in modeling the full macro-micro coupling in a time-dependent, dynamical system. Important progress may be made through existing state-of-art simulations (e.g., embedded PIC simulations Daldorff et al. 2014; Tóth et al. 2016) and the development of other novel numerical techniques (Shay et al. 2025, this collection). Different macro-micro couplings are summarized in reconnection phase diagrams (Ji and Daughton 2011; Ji et al. 2022) based on the previously mentioned plasmoid instability of long current sheets. During macro-micro coupling, key questions to ask are where and how a current sheet forms in a given global context, when reconnection can be triggered, and how efficiently it works. Such macro-micro coupling, for instance, includes reconnection within Kelvin-Helmholtz vortices (e.g., Nakamura et al. 2022; Blasl et al. 2023), other MHD-scale instabilities (e.g., Kliem and Török 2006; Zuccarello et al. 2014), solar wind-magnetosphere coupling (e.g., Dorelli 2019), solar flares (e.g., Wyper et al. 2017; Dahlin et al. 2022). More discussion can be found in Hwang et al. (2023).

The growing effort in space exploration [e.g., BepiColombo (Heyner et al. 2021) at Mercury, Juno (Bolton et al. 2017) at Jupiter, etc.] provides exciting opportunities to perform comparative studies of planetary magnetospheric reconnection; more discussion can be found in Gershman et al. (2024, this collection) and Fuselier et al. (2024, this collection). Both ground and spaceborne remote sensing/imaginary will further enable our understanding of solar flares, the coronal heating problem, and solar wind drivers; more discussion can be found in Drake et al. (2025, this collection). Meanwhile, terrestrial laboratory experiments [e.g., MRX (Yamada et al. 1997), TS-3/4 (Ono et al. 1993), TREX (Olson et al. 2016), PHASMA (Shi et al. 2022), FLARE (Ji et al. 2018), etc.] provide invaluable studies performed in a controlled, repeatable manner; more discussion can be found in Ji et al. (2023, this collection). Our hope is that what we have learned from magnetic reconnection within our solar system can also be used to understand other astrophysical objects in the Universe, such as the magnetospheres of stars, exoplanets, and the extreme plasmas near compact objects, including black holes and neutron stars; more discussion can be found in Guo et al. (2024, this collection). Going forward, continuous communication across disciplines will be the key to making breakthroughs in understanding this fundamental, exciting plasma process.

## Footnotes

1 More precisely, any flow with a velocity $\mathbf{V}$ that satisfies $\nabla \times ({\mathbf{E}}+{\mathbf{V}}\times {\mathbf{B}}/c)=0$ can be regarded as the magnetic flux preserving flow (Vasyliunas 1972).

2 Note that this location, where $B_{L}=-35\text{ nT}\sim 0.5\ B_{0}$, is considered to be close to the edge of the diffusion region.

## References

[CR1] Abdo AA, Ackermann M, Ajello M, et al. (2011) Gamma-ray flares from the Crab Nebula. Science 331:739 21212321 10.1126/science.1199705

[CR2] Alfvén H (1942) Existence of electromagnetic-hydrodynamic waves. Nature 150:405

[CR3] André M, Vaivads A, Khotyaintsev YV, et al. (2010) Magnetic reconnection and cold plasma at the magnetopause. Geophys Res Lett 37(22):L22108

[CR4] Angelopoulos V (2008) The THEMIS Mission. Space Sci Rev 141(1–4):5–34. 10.1007/s11214-008-9336-1

[CR5] Angelopoulos V, McFadden JP, Larson D, et al. (2008) Tail reconnection triggering substorm onset. Science 321:931–935 18653845 10.1126/science.1160495

[CR6] Arencibia M, Cassak PA, Shay MA, et al. (2023) Three-dimensional magnetic reconnection spreading in current sheets of non-uniform thickness. J Geophys Res Space Phys 128:e2022JA030999

[CR7] Argall MR, Barbhuiya MH, Cassak PA, et al. (2022) Theory, observations, and simulations of kinetic entropy in a magnetotail electron diffusion region. Phys Plasmas 29(2):022902. 10.1063/5.0073248. https://aip.scitation.org/doi/10.1063/5.0073248

[CR8] Asenjo FA, Comisso L (2015) Generalized magnetofluid connections in relativistic magnetohydrodynamics. Phys Rev Lett 114(11):115003. 10.1103/PhysRevLett.114.115003. arXiv:1502.07461 [physics.plasm-ph] 25839284 10.1103/PhysRevLett.114.115003

[CR9] Aunai N, Belmont G, Smets R (2011) Proton acceleration in antiparallel collisionless magnetic reconnection: kinetic mechanisms behind the fluid dynamics. J Geophys Res 116:A09232

[CR10] Aunai N, Hesse M, Kuznetsova M (2013) Electron nongyrotropy in the context of collisionless magnetic reconnection. Phys Plasmas 20:092903

[CR11] Aunai N, Hesse M, Lavraud B, et al. (2016) Orientation of the x-line in asymmetric magnetic reconnection. J Plasmas Phys 82:535820401

[CR12] Aydemir AY (1991) Linear studies of modes in high-temperature plasmas with a four-field model. Phys Fluids B 3(11):3025

[CR13] Aydemir A (1992) Nonlinear studies of modes in high-temperature plasmas. Phys Fluids B 4:3469

[CR14] Bale SD, Drake JF, McManus MD, et al. (2023) Interchange reconnection as the source of the fast solar wind within coronal holes. Nature 618:252 37286648 10.1038/s41586-023-05955-3 PMC10247371

[CR15] Bandyopadhyay R, Matthaeus WH, Parashar TN, et al. (2020) Statistics of kinetic dissipation in the Earth’s magnetosheath: MMS observations. Phys Rev Lett 124:255101. 10.1103/PhysRevLett.124.255101 32639771 10.1103/PhysRevLett.124.255101

[CR16] Bandyopadhyay R, Chasapis A, Matthaeus WH, et al. (2021) Energy dissipation in turbulent reconnection. Phys Plasmas 28(11):112305. 10.1063/5.0071015. https://aip.scitation.org/doi/10.1063/5.0071015

[CR17] Barbhuiya MH, Cassak PA (2022) Pressure-strain interaction: III. Particle-in-cell simulations of magnetic reconnection. Phys Plasmas 29:122308

[CR18] Barbhuiya MH, Cassak PA, Adhikari S, et al. (2024) Higher-order nonequilibrium term: effective power density quantifying evolution towards or away from local thermodynamic equilibrium. Phys Rev E 109:015205. 10.1103/PhysRevE.109.015205 38366463 10.1103/PhysRevE.109.015205

[CR19] Beidler MT, Cassak PA (2011) Model for incomplete reconnection in sawtooth crashes. Phys Rev Lett 107:255002 22243083 10.1103/PhysRevLett.107.255002

[CR20] Bessho N, Bhattacharjee A (2005) Collisionless reconnection in an electron-positron plasma. Phys Rev Lett 95(24):245001. 10.1103/PhysRevLett.95.245001 16384388 10.1103/PhysRevLett.95.245001

[CR21] Bessho N, Bhattacharjee A (2010) Fast magnetic reconnection in low-density electron-positron plasmas. Phys Plasmas 17(10):102104. 10.1063/1.3488963

[CR22] Bessho N, Bhattacharjee A (2014) Instability of the current sheet in the Earth’s magnetotail with normal magnetic field. Phys Plasmas 21(10):102905. 10.1063/1.4899043

[CR23] Bessho N, Chen LJ, Wang S, et al. (2019) Magnetic reconnection in a quasi-parallel shock: two-dimensional local particle-in-cell simulation. Geophys Res Lett 46(16):9352–9361. 10.1029/2019GL083397

[CR24] Bessho N, Chen LJ, Wang S, et al. (2020) Magnetic reconnection and kinetic waves generated in the Earth’s quasi-parallel bow shock. Phys Plasmas 27(9):092901. 10.1063/5.0012443

[CR25] Bessho N, Chen LJ, Stawarz JE, et al. (2022) Strong reconnection electric fields in shock-driven turbulence. Phys Plasmas 29(4):042304. 10.1063/5.0077529

[CR26] Bhattacharjee A (2004) Impulsive magnetic reconnection in the Earth’s magnetotail and the solar corona. Annu Rev Astron Astrophys 42:365

[CR27] Bhattacharjee A, Huang YM, Yang H, et al. (2009) Fast reconnection in high-Lundquist-number plasmas due to secondary tearing instabilities. Phys Plasmas 16:112102

[CR28] Birn J, Hesse M (2010) Energy release and transfer in guide field reconnection. Phys Plasmas 17:012109

[CR29] Birn J, Priest ER (2007) Reconnection of magnetic fields: magnetohydrodynamics and collisionless theory and observations

[CR30] Birn J, Drake JF, Shay MA, et al. (2001) Geospace environmental modeling (GEM) magnetic reconnection challenge. J Geophys Res 106(A3):3715–3719

[CR31] Birn J, Galsgaard K, Hesse M, et al. (2005) Forced magnetic reconnection. Geophys Res Lett 32:L06105. 10.1029/2004GL022058

[CR32] Birn J, Borovsky JE, Hesse M, et al. (2010) Scaling of asymmetric reconnection in compressible plasmas. Phys Plasmas 17:052108

[CR33] Biskamp D (1982) Effect of secondary tearing instability on the coalescence of magnetic islands. Phys Lett A 87(7):357–360. 10.1016/0375-9601(82)90844-1

[CR34] Biskamp D (1986) Magnetic reconnection via current sheets. Phys Fluids 29(5):1520–1531

[CR35] Biskamp D, Drake JF (1994) Dynamics of the sawtooth collapse in tokamak plasmas. Phys Rev Lett 73:971 10057587 10.1103/PhysRevLett.73.971

[CR36] Blackman EG, Field GB (1994) Kinematics of relativistic magnetic reconnection. Phys Rev Lett 72:494 10056447 10.1103/PhysRevLett.72.494

[CR37] Blasl KA, Nakamura TKM, Nakamura R, et al. (2023) Electron-scale reconnecting current sheet formed within the lower hybrid wave-active region of Kelvin-Helmholtz waves. Geophys Res Lett 50:e2023GL104309

[CR38] Bolton SJ, Lunine J, Stevenson D, et al. (2017) The Juno Mission. Space Sci Rev 213(1–4):5–37. 10.1007/s11214-017-0429-6

[CR39] Boozer AH (2012) Separation of magneitc field lines. Phys Plasmas 19:112901

[CR40] Borovsky JE, Hesse M, Birn J, et al. (2008) What determines the reconnection rate at the dayside magnetosphere? J Geophys Res 113:A07210

[CR41] Büchner J, Kuska JP, Nikutowski B, et al. (1998) Three-dimensional reconnection in the Earth’s magnetotail: simulations and observations. Geophys Monogr 104:313–326. 10.1029/GM104p0313

[CR42] Burch JL, Phan TD (2016) Magnetic reconnection at the dayside magnetopause: advances with MMS. Geophys Res Lett 43(16):8327–8338. 10.1002/2016GL069787

[CR43] Burch JL, Torbert RB (eds) (2016) Magnetospheric multiscale: a mission to investigate the physics of magnetic reconnection. Space Sci Rev 199(1–4)

[CR44] Burch JL, Moore TE, Torbert RB, et al. (2016a) Magnetospheric multiscale overview and science objectives. Space Sci Rev 199(1–4):5–21. 10.1007/s11214-015-0164-9

[CR45] Burch JL, Torbert RB, Phan T, et al. (2016b) Electron-scale measurement of magnetic reconnection in space. Science 352:6290 10.1126/science.aaf2939 27174677

[CR46] Burch JL, Ergun RE, Cassak PA, et al. (2018) Localized oscillatory energy conversion in magnetopause reconnection. Geophys Res Lett 45(3):1237–1245. 10.1002/2017GL076809. arXiv:1712.05697 [physics.space-ph]

[CR47] Burch JL, Webster JM, Hesse M, et al. (2020) Electron inflow velocities and reconnection rates at Earth’s magnetopause and magnetosheath. Geophys Res Lett 47(17):e89082. 10.1029/2020GL089082

[CR48] Burch JL, Hesse M, Webster JM, et al. (2022) The EDR inflow region of a reconnecting current sheet in the geomagnetic tail. Phys Plasmas 29(5):052903. 10.1063/5.0083169

[CR49] Cai HJ, Lee LC (1997) The generalized Ohm’s law in collisionless magnetic reconnection. Phys Plasmas 4(3):509–520

[CR50] Carilli CL, Taylor GB (2002) Cluster magnetic fields. Annu Rev Astron Astrophys 40:319–348. 10.1146/annurev.astro.40.060401.093852. arXiv:astro-ph/0110655 [astro-ph]

[CR51] Carmichael H (1964) A process for flares. In: Ness WN (ed) AAS/NASA symposium on the physics of solar flares. NASA, Washington, p 451

[CR52] Cassak PA (2011) Theory and simulations of the scaling of magnetic reconnection with symmetric shear flow. Phys Plasmas 18:072106

[CR53] Cassak PA, Barbhuiya MH (2022) Pressure-strain interaction: I. On compression, deformation, viscosity, and their relation to Pi-D. Phys Plasmas 29:122306

[CR54] Cassak PA, Otto A (2011) Scaling of the magnetic reconnection rate with symmetric shear flow. Phys Plasmas 18:074501. 10.1063/1.3609771

[CR55] Cassak PA, Shay MA (2007) Scaling of asymmetric magnetic reconnection: general theory and collisional simulations. Phys Plasmas 14:102114

[CR56] Cassak PA, Shay MA (2008) Scaling of asymmetric Hall magnetic reconnection. Geophys Res Lett 35:L19102 10.1002/2017GL076460 PMC6142184 30245534

[CR57] Cassak PA, Shay MA (2009) Structure of the dissipation region in fluid simulations of asymmetric magnetic reconnection. Phys Plasmas 16:055704

[CR58] Cassak PA, Shay MA, Drake JF (2005) Catastrophe model for fast magnetic reconnection onset. Phys Rev Lett 95:235002 16384311 10.1103/PhysRevLett.95.235002

[CR59] Cassak PA, Drake JF, Shay MA (2007) Catastrophe onset of fast magnetic reconnection with a guide field. Phys Plasmas 14:054502 10.1103/PhysRevLett.98.215001 17677781

[CR60] Cassak PA, Genestreti KJ, Burch JL, et al. (2017a) The effect of a guide field on local energy conversion during asymmetric magnetic reconnection: particle-in-cell simulations. J Geophys Res Space Phys 122(11):11523–11542. 10.1002/2017JA024555

[CR61] Cassak PA, Liu Y-H, Shay MA (2017b) A review of the 0.1 reconnection rate problem. J Plasma Phys 83:715830501

[CR62] Cassak PA, Barbhuiya MH, Weldon HA (2022) Pressure-strain interaction: II. Decomposition in magnetic field-aligned coordinates. Phys Plasmas 29:122307

[CR63] Cassak PA, Barbhuiya MH, Liang H, et al. (2023) Quantifying energy conversion in higher-order phase space density moments in plasmas. Phys Rev Lett 130(8):085201. 10.1103/PhysRevLett.130.085201 36898122 10.1103/PhysRevLett.130.085201

[CR64] Cerutti B, Werner GR, Uzdensky DA, et al. (2014) Gamma-ray flares in the Crab Nebula: a case of relativistic reconnection?. Phys Plasmas 21(5):056501. 10.1063/1.4872024. arXiv:1401.3016 [astro-ph.HE]

[CR65] Chanteur G (1998) Spatial interpolation for four spacecraft: theory. In: Analysis methods for multi-spacecraft data. ISSI scientific reports series, vol 1, pp 349–370

[CR66] Chasapis A, Yang Y, Matthaeus WH, et al. (2018) Energy conversion and collisionless plasma dissipation channels in the turbulent magnetosheath observed by the magnetospheric multiscale mission. Astrophys J 862(1):32. 10.3847/1538-4357/aac775

[CR67] Che H, Drake JF, Swisdak M (2011a) A current filamentation mechanism for breaking field magnetic field lines during reconnection. Nature 474:184–187. 10.1038/nature10091 21633355 10.1038/nature10091

[CR68] Che H, Goldman MV, Newman DL (2011) Buneman instability in a magnetized current-carrying plasma with velocity shear. Phys Plasmas 18(5):052109. 10.1063/1.3590879. arXiv:1104.5283 [physics.plasm-ph]

[CR69] Chen XL, Morrison PJ (1990) Resistive tearing instability with equilibrium shear flow. Phys Fluids B 2:495

[CR70] Chen LJ, Hesse M, Wang S, et al. (2016) Electron energization and structure of the diffusion region during asymmetric reconnection. Geophys Res Lett 43:2405

[CR71] Chen LJ, Hesse M, Wang S, et al. (2017) Electron diffusion region during magnetopause reconnection with an intermediate guide field: magnetospheric multiscale observations. J Geophys Res Space Phys 122(5):5235–5246. 10.1002/2017JA024004

[CR72] Chen LJ, Wang S, Hesse M, et al. (2019) Electron diffusion regions in magnetotail reconnection under varying guide fields. Geophys Res Lett 46(12):6230–6238. 10.1029/2019GL082393

[CR73] Chien A, Gao L, Zhang S, et al. (2023) Non-thermal electron acceleration from magnetically driven reconnection in a laboratory plasma. Nat Phys 19(2):254–262. 10.1038/s41567-022-01839-x. arXiv:2201.10052 [physics.plasm-ph]

[CR74] Comisso L, Lingam M, Huang YM, et al. (2016) General theory of the plasmoid instability. Phys Plasmas 23:100702

[CR75] Coppi B (1965) Current-driven instabilities in configurations with sheared magnetic fields. Phys Fluids 8:2273

[CR76] Coppi B, Laval G, Pellat R (1966) Dynamics of the geomagnetic tail. Phys Rev Lett 16(26):1207–1210. 10.1103/PhysRevLett.16.1207

[CR77] Coroniti FV (1985) Explosive tail reconnection: the growth and expansion phases of magnetospheric substorms. J Geophys Res 90(A8):7427–7448. 10.1029/JA090iA08p07427

[CR78] Dahlin JT, Antiochos SK, Qiu J, et al. (2022) Variability of the reconnection guide field in solar flares. Astrophys J 932(2):94. 10.3847/1538-4357/ac6e3d. arXiv:2110.04132 [astro-ph.SR]

[CR79] Daldorff LKS, Tóth G, Gombosi TI, et al. (2014) Two-way coupling of a global Hall magnetohydrodynamics model with a local implicit particle-in-cell model. J Comput Phys 268:236–254. 10.1016/j.jcp.2014.03.009

[CR80] Dargent J, Aunai N, Lavraud B, et al. (2017) Kinetic simulation of asymmetric magnetic reconnection with cold ions. J Geophys Res Space Phys 122(5):5290–5306

[CR81] Dargent J, Aunai N, Lavraud B, et al. (2020) Simulation of plasmaspheric plume impact on dayside magnetic reconnection. Geophys Res Lett 47(4):e2019GL086546

[CR82] Daughton W, Roytershteyn V, Albright BJ, et al. (2009) Transition from collisional to kinetic regimes in large-scale reconnection layers. Phys Rev Lett 103:065004 19792577 10.1103/PhysRevLett.103.065004

[CR83] Daughton W, Roytershteyn V, Karimabadi H, et al. (2011) Role of electron physics in the development of turbulent magnetic reconnection in collisionless plasmas. Nat Phys 7:539–542. 10.1038/nphys1965

[CR84] Daughton W, Nakamura TKM, Karimabadi H, et al. (2014) Computing the reconnection rate in turbulent kinetic layers by using electron mixing to identify topology. Phys Plasmas 21:052307

[CR85] Davidson RC, Gladd NT (1975) Anomalous transport properties associated with the lower-hybrid-drift instability. Phys Fluids 18(10):1327–1335. 10.1063/1.861021

[CR86] Del Sarto D, Pegoraro F (2018) Shear-induced pressure anisotropization and correlation with fluid vorticity in a low collisionality plasma. Mon Not R Astron Soc 475:181. 10.1093/mnras/stx3083

[CR87] Del Sarto D, Pegoraro F, Califano F (2016) Pressure anisotropy and small spatial scales induced by velocity shear. Phys Rev E 93(5):053203. 10.1103/PhysRevE.93.053203 27300991 10.1103/PhysRevE.93.053203

[CR88] Denton RE, Drake JF, Kleva RG (1987) The convection cell and sawteeth in tokamaks. Phys Fluids 30:1448

[CR89] Desroche M, Bagenal F, Delamere PA, et al. (2012) Conditions at the expanded Jovian magnetopause and implications for the solar wind interaction. J Geophys Res 117:A07202. 10.1029/2012JA017621

[CR90] DiBraccio GA, Slavin JA, Boardsen SA, et al. (2013) MESSENGER observations of magnetopause structure and dynamics at Mercury. J Geophys Res 118:997–1008. 10.1002/jgra.50123

[CR91] Divin A, Khotyaintsev YV, Vaivads A, et al. (2016) Three-scale structure of diffusion region in the presence of cold ions. J Geophys Res Space Phys 121(12):12001–12013

[CR92] Dorelli JC (2019) Does the solar wind electric field control the reconnection rate at Earth’s subsolar magnetopause? J Geophys Res Space Phys 124(4):2668–2681. 10.1029/2018JA025868

[CR93] Dorelli JC, Hesse M, Kuznetsova MM, et al. (2004) A new look at driven magnetic reconnection at the terrestrial subsolar magnetopause. J Geophys Res 109:A12216

[CR94] Doss C, Komar C, Cassak P, et al. (2015) Asymmetric magnetic reconnection with a flow shear and applications to the magnetopause. J Geophys Res Space Phys 120(9):7748–7763

[CR95] Drake JF, Swisdak M, Hesse M (2004) The structure of the parallel electric field during magnetic reconnection. In: EOS trans. AGU, pp Abstract SM42A–05

[CR96] Drake JF, Shay MA, Swisdak M (2008) The Hall fields and fast magnetic reconnection. Phys Plasmas 15(4):042306. 10.1063/1.2901194

[CR97] Drake JF, Opher M, Swisdak M, et al. (2010) A magnetic reconnection mechanism for the generation of anomalous cosmic rays. Astrophys J 709:963–974. 10.1088/0004-637X/709/2/963

[CR98] Drake JF, Antiochos SK, Bale SD, et al (2025) Magnetic reconnection in solar flares and the near-Sun solar wind. Space Sci Rev 221

[CR99] Du S, Guo F, Zank GP, et al. (2018) Plasma energization in colliding magnetic flux ropes. Astrophys J 867:16. 10.3847/1538-4357/aae30e

[CR100] Du S, Zank GP, Li X, et al. (2020) Energy dissipation and entropy in collisionless plasma. Phys Rev E 101:033208. 10.1103/PhysRevE.101.033208 32289904 10.1103/PhysRevE.101.033208

[CR101] Dungey JW (1953) Conditions for the occurrence of electrical discharges in astrophysical systems. Philos Mag 44:725

[CR102] Dungey JW (1961) Interplanetary magnetic field and the auroral zones. Phys Rev Lett 6(2):47–48. 10.1103/PhysRevLett.6.47

[CR103] Dungey JW (1988) Noise-free neutral sheet. In: Proceedings of an international workshop in space plasma, ESA SP-285, p 15

[CR104] Eastwood JP, Shay MA, Phan TD, et al. (2010) Asymmetry of the ion diffusion region Hall electric and magnetic fields during guide field reconnection: observations and comparison with simulations. Phys Rev Lett 104(20):205001. 10.1103/PhysRevLett.104.205001 20867032 10.1103/PhysRevLett.104.205001

[CR105] Eastwood JP, Phan TD, Drake JF, et al. (2013) Energy partition in magnetic reconnection in Earth’s magnetotail. Phys Rev Lett 110:225001 23767730 10.1103/PhysRevLett.110.225001

[CR106] Eastwood JP, Goldman MV, Phan TD, et al. (2020) Energy flux densities near the electron dissipation region in asymmetric magnetopause reconnection. Phys Rev Lett 125:265102 33449730 10.1103/PhysRevLett.125.265102

[CR107] Egedal J, Le A, Daughton W (2013) A review of pressure anisotropy caused by electron trapping in collisionless plasma, and its implications for magnetic reconnection. Phys Plasmas 20:061201

[CR108] Egedal J, Ng J, Le A, et al. (2019) Pressure tensor elements breaking the frozen-in law during reconnection in Earth’s magnetotail. Phys Rev Lett 123(22):225101. 10.1103/PhysRevLett.123.225101 31868399 10.1103/PhysRevLett.123.225101

[CR109] Ergun RE, Goodrich KA, Wilder FD, et al. (2016) Magnetospheric multiscale satellites observations of parallel electric fields associated with magnetic reconnection. Phys Rev Lett 116:235102 27341241 10.1103/PhysRevLett.116.235102

[CR110] Ergun RE, Goodrich KA, Wilder FD, et al. (2018) Magnetic reconnection, turbulence, and particle acceleration: observations in the Earth’s magnetotail. Geophys Res Lett 45:3338

[CR111] Ergun RE, Pathak N, Usanova ME, et al. (2022) Observation of magnetic reconnection in a region of strong turbulence. Astrophys J Lett 935(1):L8. 10.3847/2041-8213/ac81d4

[CR112] Eriksson S, Lavraud B, Wilder FD, et al. (2016a) Magnetospheric multiscale observations of magnetic reconnection associated with Kelvin-Helmholtz waves. Geophys Res Lett 43(11):5606–5615. 10.1002/2016GL068783

[CR113] Eriksson S, Wilder FD, Ergun RE, et al. (2016b) Magnetospheric multiscale observations of the electron diffusion region of large guide field magnetic reconnection. Phys Rev Lett 117(1):015001. 10.1103/PhysRevLett.117.015001 27419573 10.1103/PhysRevLett.117.015001

[CR114] Escoubet CP, Fehringer M, Goldstein M (2001) Introduction: the Cluster mission. Ann Geophys 19:1197–1200. 10.5194/angeo-19-1197-2001

[CR115] Eyink GL, Lazarian A, Vishniac ET (2011) Fast magnetic reconnection and spontaneous stochasticity. Astrophys J 743(1):51. 10.1088/0004-637X/743/1/51. arXiv:1103.1882 [astro-ph.GA]

[CR116] Fadanelli S, Lavraud B, Califano F, et al. (2021) Energy conversions associated with magnetic reconnection. J Geophys Res Space Phys 126(1):A028333. 10.1029/2020JA028333

[CR117] Fujimoto K, Sydora RD (2012) Plasmoid-induced turbulence in collisionless magnetic reconnection. Phys Rev Lett 109(26):265004. 10.1103/PhysRevLett.109.265004 23368574 10.1103/PhysRevLett.109.265004

[CR118] Fuselier SA, Lewis WS, Schiff C, et al. (2016) Magnetospheric multiscale science mission profile and operations. Space Sci Rev 199(1–4):77–103. 10.1007/s11214-014-0087-x

[CR119] Fuselier SA, Petrinec SM, Reiff PH, et al. (2024) Global-scale processes and effects of magnetic reconnection on the geospace environment. Space Sci Rev 220(4):34. 10.1007/s11214-024-01067-0

[CR120] Genestreti KJ, Burch JL, Cassak PA, et al. (2017) The effect of a guide field on local energy conversion during asymmetric magnetic reconnection: MMS observations. J Geophys Res Space Phys 122(11):11342–11353. 10.1002/2017JA024247. arXiv:1706.08404 [physics.space-ph]

[CR121] Genestreti KJ, Cassak PA, Varsani A, et al. (2018a) Assessing the time dependence of reconnection with Poynting’s theorem: MMS observations. Geophys Res Lett 45:2886

[CR122] Genestreti KJ, Nakamura TKM, Nakamura R, et al. (2018b) How accurately can we measure the reconnection rate for the MMS diffusion region event of 11 July 2017? J Geophys Res Space Phys 123(11):9130–9149. 10.1029/2018JA025711. arXiv:1808.03603 [physics.space-ph] 30775197 10.1029/2018JA025711 PMC6360497

[CR123] Genestreti KJ, Varsani A, Burch JL, et al. (2018c) MMS observation of asymmetric reconnection supported by 3-D electron pressure divergence. J Geophys Res Space Phys 123(3):1806–1821. 10.1002/2017JA025019. arXiv:1711.08262 [physics.space-ph]

[CR124] Genestreti KJ, Li X, Liu YH, et al. (2022) On the origin of “patchy” energy conversion in electron diffusion regions. Phys Plasmas 29(8):082107. 10.1063/5.0090275. arXiv:2203.13879 [physics.space-ph]

[CR125] Genestreti KJ, Nakamura R, Burch JL, et al (2025) Structure of the electron diffusion region during magnetic reconnection. Space Sci Rev 221 10.1007/s11214-025-01145-x PMC11821752 39958871

[CR126] Gershman D, Fuselier S, Cohen I, et al. (2024) Magnetic reconnection at planetary bodies and astrospheres. Space Sci Rev 220:7. 10.1007/s11214-023-01017-2

[CR127] Gingell I, Schwartz SJ, Eastwood JP, et al. (2019) Observations of magnetic reconnection in the transition region of quasi-parallel shocks. Geophys Res Lett 46(3):1177–1184. 10.1029/2018GL081804. arXiv:1901.01076 [physics.space-ph]

[CR128] Gingell I, Schwartz SJ, Eastwood JP, et al. (2020) Statistics of reconnecting current sheets in the transition region of Earth’s bow shock. J Geophys Res Space Phys 125(1):e27119. 10.1029/2019JA027119

[CR129] Goldman MV, Newman DL, Eastwood JP, et al. (2020) Multibeam energy moments of multibeam particle velocity distributions. J Geophys Res Space Phys 125(12):e28340. 10.1029/2020JA028340. arXiv:2005.09113 [physics.plasm-ph]

[CR130] Gonzalez WD, Parker EN (eds) (2016) Magnetic reconnection: concepts and applications. Astrophysics and Space Science Library, vol 427. Springer, Cham. 10.1007/978-3-319-26432-5

[CR131] Goodbred M, Liu YH (2022) First-principle theory of the relativistic magnetic reconnection rate in astrophyical pair plasmas. Phys Rev Lett 129:265101. 10.1103/PhysRevLett.129.265101 36608210 10.1103/PhysRevLett.129.265101

[CR132] Grad H (1965) On Boltzmann’s H-theorem. J Soc Ind Appl Math 13(1):259–277. http://www.jstor.org/stable/2946404

[CR133] Graham DB, Khotyaintsev YV, André M, et al. (2022) Direct observations of anomalous resistivity and diffusion in collisionless plasma. Nat Commun 13:2954. 10.1038/s41467-022-30561-8 35618713 10.1038/s41467-022-30561-8 PMC9135766

[CR134] Graham DB, Cozzani G, Khotyaintsev YV, et al (2025) The role of kinetic instabilities and waves in collisionless magnetic reconnection. Space Sci Rev 221. 10.1007/s11214-024-01133-7

[CR135] Greess S, Egedal J, Stanier A, et al. (2022) Kinetic simulations verifying reconnection rates measured in the laboratory, spanning the ion-coupled to near electron-only regimes. Phys Plasmas 29(10):102103. 10.1063/5.0101006. arXiv:2210.04960 [physics.plasm-ph]

[CR136] Guan Y, Lu Q, Lu S, et al. (2023) Reconnection rate and transition from ion-coupled to electron-only reconnection. Astrophys J 958(2):172. 10.3847/1538-4357/ad05b8

[CR137] Guo F, Liu Y, Zenitani S, et al. (2024) Magnetic reconnection and associated particle acceleration in high-energy astrophysics. Space Sci Rev 220:43. 10.1007/s11214-024-01073-2. arXiv:2309.13382

[CR138] Haggerty C, Shay M, Drake J, et al. (2015) The competition of electron and ion heating during magnetic reconnection. Geophys Res Lett 42(22):9657–9665

[CR139] Haggerty CC, Parashar TN, Matthaeus WH, et al. (2017) Exploring the statistics of magnetic reconnection X-points in kinetic particle-in-cell turbulence. Phys Plasmas 24(10):102308. 10.1063/1.5001722. arXiv:1706.04905 [physics.space-ph]

[CR140] Haggerty C, Shay M, Chasapis A, et al. (2018) The reduction of magnetic reconnection outflow jets to sub-Alfvénic speeds. Phys Plasmas 25:102120. 10.1063/1.5050530

[CR141] Hasegawa H, Denton RE, Nakamura R, et al. (2019) Reconstruction of the electron diffusion region of magnetotail reconnection seen by the MMS spacecraft on 11 July 2017. J Geophys Res Space Phys 124(1):122–138. 10.1029/2018JA026051

[CR142] Hasegawa H, Argall MR, Aunai N, et al. (2024) Advanced methods for analyzing in-situ observations of magnetic reconnection. Space Sci Rev 220:68. 10.1007/s11214-024-01095-w 39234211 10.1007/s11214-024-01095-w PMC11369046

[CR143] Hellinger P, Montagud-Camps V, Franci L, et al. (2022) Ion-scale transition of plasma turbulence: pressure–strain effect. Astrophys J 930:48

[CR144] Hesse M, Cassak P (2020) Magnetic reconnection in the space science: past, present, and future. J Geophys Res 125:e2018JA025935

[CR145] Hesse M, Schindler K (1988) A theoretical foundation of general magnetic reconnection. J Geophys Res 93(A6):5559–5567

[CR146] Hesse M, Schindler K (2001) The onset of magnetic reconnection in the magnetotail. Earth Planets Space 53:645–653

[CR147] Hesse M, Schindler K, Birn J, et al. (1999) The diffusion region in collisionless magnetic reconnection. Phys Plasmas 6:1781–1795

[CR148] Hesse M, Kuznetsova M, Schindler K, et al. (2005) Three-dimensional modeling of electron quasiviscous dissipation in guide-field magnetic reconnection. Phys Plasmas 12(10):100704. 10.1063/1.2114350

[CR149] Hesse M, Neukirch T, Schindler K, et al. (2011) The diffusion region in collisionless magnetic reconnection. Space Sci Rev 160:3–23. 10.1007/s11214-010-9740-1

[CR150] Hesse M, Aunai N, Zenitani S, et al. (2013) Aspects of collisionless magnetic reconnection in asymmetric systems. Phys Plasmas 20:061210

[CR151] Hesse M, Aunai N, Sibeck D, et al. (2014) On the electron diffusion region in planar, asymmetric systems. Geophys Res Lett 41:8673

[CR152] Hesse M, Liu YH, Chen LJ, et al. (2018) The physical foundation of the reconnection electric field. Phys Plasmas 25:032901

[CR153] Hesse M, Norgren C, Tenfjord P, et al. (2021) A new look at the electron diffusion region in asymmetric magnetic reconnection. J Geophys Res Space Phys 126(2):e28456. 10.1029/2020JA028456. arXiv:2007.03379 [physics.space-ph]

[CR154] Heuer SV, Genestreti KJ, Nakamura TKM, et al. (2022) Calculating the electron diffusion region aspect ratio with magnetic field gradients. Geophys Res Lett 49(20):e2022GL100652. 10.1029/2022GL100652

[CR155] Heyner D, Auster H, Fornacon K, et al. (2021) The BepiColombo planetary magnetometer MPO-MAG: what can we learn from the Hermean magnetic field? Space Sci Rev 217:52. 10.1007/s11214-021-00822-x

[CR156] Higashimori K, Yokoi N, Hoshino M (2013) Explosive turbulent magnetic reconnection. Phys Rev Lett 110(25):255001. 10.1103/PhysRevLett.110.255001. arXiv:1305.6695 [astro-ph.EP] 23829741 10.1103/PhysRevLett.110.255001

[CR157] Hirayama T (1974) Theoretical model of flares and prominences. I: evaporating flare model. Sol Phys 34:323

[CR158] Holmes JC, Nakamura R, Schmid D, et al. (2021) Wave activity in a dynamically evolving reconnection separatrix. J Geophys Res 126:e2020JA028520

[CR159] Hornig G, Schindler K (1996) Magnetic topology and the problem of its invariant definition. Phys Plasmas 3(3):781–791. 10.1063/1.871778

[CR160] Hoshino M (2018) Energy partition between ion and electron of collisionless magnetic reconnection. Astrophys J Lett 868:L18

[CR161] Hoshino M, Nishida A (1983) Numerical simulation of the dayside magnetopause. J Geophys Res 88:6926

[CR162] Hosner M, Nakamura R, Schmid D, et al. (2024) Reconnection inside a dipolarization front of a diverging earthward fast flow. J Geophys Res Space Phys 129(1):e2023JA031976. 10.1029/2023JA031976

[CR163] Huang YM, Comisso L, Bhattacharjee A (2017) Plasmoid instability in evolving current sheets and onset of fast reconnection. Astrophys J 849(2):75. 10.3847/1538-4357/aa906d. arXiv:1707.01863 [physics.plasm-ph]

[CR164] Huang K, Liu YH, Lu Q, et al. (2020) Scaling of magnetic reconnection with a limited x-line extent. J Geophys Lett 47:e2020GL088147

[CR165] Huang SY, Xiong QY, Song LF, et al. (2021) Electron-only reconnection in an ion-scale current sheet at the magnetopause. Astrophys J 922(1):54. 10.3847/1538-4357/ac2668. arXiv:2109.13051 [physics.plasm-ph]

[CR166] Huang K, Liu YH, Lu Q, et al. (2022) Auroral spiral structure formation through magnetic reconnection in the aurora acceleration region. J Geophys Lett 49:e2022GL100466

[CR167] Huba J (2005) Hall magnetic reconnection: guide field dependence. Phys Plasmas 12:012322

[CR168] Huba JD (2006) Forced Hall magnetic reconnection: parametric variation of the “Newton challenge”. Phys Plasmas 12:062311. 10.1063/1.2212397

[CR169] Huba JD, Rudakov LI (2002) Three-dimensional Hall magnetic reconnection. Phys Plasmas 9(11):4435–4438

[CR170] Huba JD, Rudakov LI (2003) Hall magnetohydrodynamics of neutral layers. Phys Plasmas 10(8):3139–3150

[CR171] Hwang KJ, Dokgo K, Choi E, et al. (2020) Magnetic reconnection inside a flux rope induced by Kelvin-Helmholtz vortices. J Geophys Res Space Phys 125(4):e27665. 10.1029/2019JA027665 10.1029/2019JA027665 PMC7375157 32714734

[CR172] Hwang KJ, Dokgo K, Choi E, et al. (2021) Bifurcated current sheet observed on the boundary of Kelvin-Helmholtz vortices. Front Astron Space Sci 8:201. 10.3389/fspas.2021.782924

[CR173] Hwang KJ, Nakamura R, Eastwood JP, et al. (2023) Cross-scale processes of magnetic reconnection. Space Sci Rev 219(8):71. 10.1007/s11214-023-01010-9

[CR174] Jain N, Büchner J (2017) Spreading of electron scale magnetic reconnection with a wave number dependent speed due to the propagation of dispersive waves. Phys Plasmas 24(8):082304. 10.1063/1.4994704

[CR175] Jain N, Sharma AS (2009) Electron scale structures in collisionless magnetic reconnection. Phys Plasmas 16(5):050704. 10.1063/1.3134045

[CR176] Jain N, Sharma AS (2015) Evolution of electron current sheets in collisionless magnetic reconnection. Phys Plasmas 22(10):102110. 10.1063/1.4933120

[CR177] Jara-Almonte J, Ji H (2021) Thermodynamic phase transition in magnetic reconnection. Phys Rev Lett 127(5):055102. 10.1103/PhysRevLett.127.055102 34397253 10.1103/PhysRevLett.127.055102

[CR178] Jara-Almonte J, Ji H, Yoo J, et al. (2019) Kinetic simulations of magnetic reconnection in partially ionized plasmas. Phys Rev Lett 122(1):015101. 10.1103/PhysRevLett.122.015101 31012658 10.1103/PhysRevLett.122.015101

[CR179] Ji H, Daughton W (2011) Phase diagram for magnetic reconnection in heliophysical, astrophysical, and laboratory plasmas. Phys Plasmas 18:111207

[CR180] Ji H, Cutler R, Gettelfinger G, et al. (2018) The FLARE device and its first plasma operation. In: APS division of plasma physics meeting abstracts, p CP11.020

[CR181] Ji H, Daughton W, Jara-Almonte J, et al. (2022) Magnetic reconnection in the era of exascale computing and multiscale experiments. Nat Rev Phys 4(4):263–282. 10.1038/s42254-021-00419-x. arXiv:2202.09004 [physics.plasm-ph]

[CR182] Ji H, Yoo J, Fox W, et al. (2023) Laboratory study of collisionless magnetic reconnection. Space Sci Rev 219(8):76. 10.1007/s11214-023-01024-3 38023292 10.1007/s11214-023-01024-3 PMC10651714

[CR183] Jiménez JPC, Tenfjord P, Hesse M, et al. (2022) The role of resistivity on the efficiency of magnetic reconnection in mhd. J Geophys Res 127:e2021JA030134

[CR184] Kadomtsev BB (1975) Disruptive instability in tokamaks. Sov J Plasma Phys 1:389–391

[CR185] Karimabadi H, Krauss-Varban D, Huba JD, et al. (2004) On magnetic reconnection regimes and associated three-dimensional asymmetries: hybrid, Hall-less hybrid, and Hall-MHD simulations. J Geophys Res Space Phys 109(A9):A09205. 10.1029/2004JA010478

[CR186] Khotyaintsev YV, Graham DB, Norgren C, et al. (2019) Collisionless magnetic reconnection and waves: progress review. Front Astron Space Sci 6:70. 10.3389/fspas.2019.00070

[CR187] Kieokaew R, Lavraud B, Foullon C, et al. (2020) Magnetic reconnection inside a flux transfer event-like structure in magnetopause Kelvin-Helmholtz waves. J Geophys Res Space Phys 125(6):e27527. 10.1029/2019JA027527

[CR188] Kletzing C (2019) The Tandem Reconnection and Cusp Electrodynamics Reconnaissance Satellites (TRACERS) Mission. In: AGU Fall Meeting abstracts, p A41U–2687

[CR189] Kleva RG, Drake JF, Waelbroeck FL (1995) Fast reconnection in high temperature plasmas. Phys Plasmas 2(1):23–34

[CR190] Kliem B, Török T (2006) Torus instability. Phys Rev Lett 96(25):255002. 10.1103/PhysRevLett.96.255002. arXiv:physics/0605217 [physics.plasm-ph] 16907312 10.1103/PhysRevLett.96.255002

[CR191] Kolstø HM, Hesse M, Norgren C, et al. (2020) Collisionless magnetic reconnection in an asymmetric oxygen density configuration. Geophys Res Lett 47(1):e2019GL085359

[CR192] Kopp R, Pneuman G (1976) Magnetic reconnection in the corona and the loop prominence phenomenon. Sol Phys 50(1):85–98

[CR193] Kulsrud RM (2001) Magnetic reconnection: Sweet-Parker versus Petschek. Earth Planets Space 53:417

[CR194] Kulsrud R, Ji H, Fox W, et al. (2005) An electromagnetic drift instability in the magnetic reconnection experiment and its importance for magnetic reconnection. Phys Plasmas 12:082301. 10.1063/1.1949225

[CR195] La Belle-Hamer AL, Otto A, Lee LC (1995) Magnetic reconnection in the presence of sheared flow and density asymmetry: applications to the Earth’s magnetopause. J Geophys Res 100:11875

[CR196] Lapenta G, Krauss-Varban D, Karimabadi H, et al. (2006) Kinetic simulations of x-line expansion in 3D reconnection. Geophys Res Lett 33:L10102. 10.1029/2005GL025124

[CR197] Lapenta G, El-Alaoui M, Berchem J, et al. (2020) Multiscale mhd-kinetic pic study of energy fluxes caused by reconnection. J Geophys Res 125:e2019JA027276

[CR198] Lazarian A, Vishniac E (1999) Reconnection in a weakly stochastic field. Astrophys J 517:700

[CR199] Le A, Egedal J, Ng J, et al. (2014) Current sheets and pressure anisotropy in the reconnection exhaust. Phys Plasmas 21(1):012103. 10.1063/1.4861871

[CR200] Le A, Daughton W, Ohia O, et al. (2018) Drift turbulence, particle transport, and anomalous dissipation at the reconnection magnetopause. Phys Plasmas 25:062103. 10.1063/1.5027086

[CR201] Lee LC, Lee KH (2020) Fluid and kinetic aspects of magnetic reconnection and some related magnetospheric phenomena. Rev Mod Plasma Phys 4(1):9. 10.1007/s41614-020-00045-7

[CR202] Lembege B, Pellat R (1982) Stability of a thick two-dimensional quasineutral sheet. Phys Fluids 25:1995

[CR203] Li X, Liu YH (2021) The effect of thermal pressure on collisionless magnetic reconnection rate. Astrophys J 912:152. 10.3847/1538-4357/abf48c

[CR204] Li W, André M, Khotyaintsev YV, et al. (2016) Kinetic evidence of magnetic reconnection due to Kelvin-Helmholtz waves. Geophys Res Lett 43(11):5635–5643. 10.1002/2016GL069192

[CR205] Li TC, Liu YH, Hesse M, et al. (2020) Three-dimensional x-line spreading in asymmetric magnetic reconnection. J Geophys Res 125:e2019JA027094

[CR206] Li TC, Liu YH, Qi Y (2021a) Identification of active magnetic reconnection using magnetic flux transport in plasma turbulence. Astrophys J Lett 909:L28

[CR207] Li T, Priest E, Guo R (2021b) Three-dimensional magnetic reconnection in astrophysical plasmas. Proc R Soc Lond Ser A 477(2249):20200949. 10.1098/rspa.2020.0949. arXiv:2104.05174 [astro-ph.SR]

[CR208] Lin SC, Liu YH, Li X (2021) Fast magnetic reconnection induced by resistivity gradients in 2D magnetohydrodynamics. Phys Plasmas 28:072109

[CR209] Liu YH, Hesse M (2016) Suppression of collisionless magnetic reconnection in asymmetric current sheets. Phys Plasmas 23:060704

[CR210] Liu YH, Drake JF, Swisdak M (2011a) The effects of strong temperature anisotropy on the kinetic structure of collisionless slow shocks and reconnection exhausts. I. Particle-in-cell simulations. Phys Plasmas 18:062110. 10.1063/1.3601760

[CR211] Liu YH, Drake JF, Swisdak M (2011b) The effects of strong temperature anisotropy on the kinetic structure of collisionless slow shocks and reconnection exhausts. II. Theory. Phys Plasmas 18:092102. 10.1063/1.3627147

[CR212] Liu YH, Drake JF, Swisdak M (2012) The structure of magnetic reconnection exhaust boundary. Phys Plasmas 19:022110. 10.1063/1.3685755

[CR213] Liu YH, Daughton W, Karimabadi H, et al. (2013) Bifurcated structure of the electron diffusion region in three-dimensional magnetic reconnection. Phys Rev Lett 110:265004 23848886 10.1103/PhysRevLett.110.265004

[CR214] Liu YH, Birn J, Daughton W, et al. (2014a) Onset of reconnection in the near magnetotail: PIC simulations. J Geophys Res 119:9773

[CR215] Liu YH, Daughton W, Karimabadi H, et al. (2014b) Do dispersive waves play a role in collisionless magnetic reconnection? Phys Plasmas 21:022113

[CR216] Liu YH, Guo F, Daughton W, et al. (2015a) Scaling of magnetic reconnection in relativistic collisionless pair plasmas. Phys Rev Lett 114:095002 25793820 10.1103/PhysRevLett.114.095002

[CR217] Liu YH, Hesse M, Kuznetsova M (2015b) Orientation of x lines in asymmetric magnetic reconnection– mass ratio dependency. J Geophys Res 120:7331

[CR218] Liu Y, Mouikis C, Kistler L, et al. (2015c) The heavy ion diffusion region in magnetic reconnection in the Earth’s magnetotail. J Geophys Res Space Phys 120(5):3535–3551

[CR219] Liu YH, Hesse M, Guo F, et al. (2017) Why does steady-state magnetic reconnection have a maximum local rate of order 0.1? Phys Rev Lett 118:085101 28282209 10.1103/PhysRevLett.118.085101

[CR220] Liu YH, Hesse M, Cassak PA, et al. (2018a) On the collisionless asymmetric magnetic reconnection rate. Geophys Res Lett 45(8):3311–3318. 10.1002/2017GL076460 30245534 10.1002/2017GL076460 PMC6142184

[CR221] Liu YH, Hesse M, Guo F, et al. (2018b) Strongly localized magnetic reconnection by the super-Alfvénic shear flow. Phys Plasmas 25:080701. 10.1063/1.5042539 30224858 10.1063/1.5042539 PMC6137741

[CR222] Liu YH, Hesse M, Li TC, et al. (2018c) Orientation and stability of asymmetric magnetic reconnection x-line. J Geophys Res 123:4908. 10.1029/2018JA025410 10.1029/2018JA025410 PMC6196328 30364510

[CR223] Liu YH, Li TC, Hesse M, et al. (2019) Three-dimensional magnetic reconnection with a spatially confined x-line extent: implications for dipolarizing flux bundles and the dawn-dusk asymmetry. J Geophys Res 124:2819. 10.1029/2019JA026539

[CR224] Liu YH, Lin SC, Hesse M, et al. (2020a) The critical role of collisionless plasma energization on the structure of relativistic magnetic reconnection. Astrophys J Lett 892:L13. 10.3847/2041-8213/ab7d3f

[CR225] Liu TZ, Lu S, Turner DL, et al. (2020b) Magnetospheric multiscale (MMS) observations of magnetic reconnection in foreshock transients. J Geophys Res Space Phys 125(4):e27822. 10.1029/2020JA027822

[CR226] Liu YH, Cassak P, Li X, et al. (2022) First-principle theory of the rate of magnetic reconnection in magnetospheric and solar plasmas. Commun Phys 5:97. 10.1038/s42005-022-00854-x

[CR227] Liu YN, Fujimoto K, Cao JB (2024) Intense magnetic reconnection process embedded in three-dimensional turbulent current sheet. Geophys Res Lett 51(1):e2023GL106466. 10.1029/2023GL106466

[CR228] Lopez RE (2016) The integrated dayside merging rate is controlled primarily by the solar wind. J Geophys Res Space Phys 121(5):4435–4445. 10.1002/2016JA022556

[CR229] Loureiro NF, Schekochihin AA, Cowley SC (2007) Instability of current sheets and formation of plasmoid chains. Phys Plasmas 14:100703. 10.1063/1.2783986

[CR230] Lu S, Pritchett PL, Angelopoulos V, et al. (2018) Magnetic reconnection in Earth’s magnetotail: energy conversion and its earthward-tailward asymmetry. Phys Plasmas 25:012905

[CR231] Lu S, Wang R, Lu Q, et al. (2020) Magnetotail reconnection onset caused by electron kinetics with a strong external driver. Nat Commun 11:5049 33028826 10.1038/s41467-020-18787-w PMC7542433

[CR232] Lyons LR, Pridmore-Brown DC (1990) Force balance near an X line in a collisionless plasma. J Geophys Res 95(A12):20903–20909. 10.1029/JA095iA12p20903

[CR233] Lyubarsky YE (2005) On the relativistic magnetic reconnection. Mon Not R Astron Soc 358:113–119

[CR234] Lyutikov M, Uzdensky D (2003) Dynamics of relativistic reconnection. Astrophys J 589:893–901

[CR235] Mahlmann JF, Philippov AA, Levinson A, et al. (2022) Electromagnetic fireworks: fast radio bursts from rapid reconnection in the compressed magnetar wind. Astrophys J Lett 932(2):L20. 10.3847/2041-8213/ac7156

[CR236] Man HY, Zhou M, Yi YY, et al. (2020) Observations of electron-only magnetic reconnection associated with macroscopic magnetic flux ropes. Geophys Res Lett 47(19):e89659. 10.1029/2020GL089659

[CR237] Mandt ME, Denton RE, Drake JF (1994) Transition to whistler mediated reconnection. Geophys Res Lett 21(1):73–76

[CR238] Markidis S, Lapenta G, Bettarini L, et al. (2011) Kinetic simulations of magnetic reconnection in presence of a background population. J Geophys Res Space Phys 116(A1):A00K16. 10.1029/2011JA016429

[CR239] Marrone DP, Moran JM, Zhao JH, et al. (2007) An unambiguous detection of Faraday rotation in Sagittarius . Astrophys J Lett 654(1):L57–L60. 10.1086/510850. arXiv:astro-ph/0611791 [astro-ph]

[CR240] Marshall AT, Burch JL, Reiff PH, et al. (2020) Asymmetric reconnection within a flux rope-type dipolarization front. J Geophys Res Space Phys 125(1):e27296. 10.1029/2019JA027296

[CR241] Masters A, Eastwood JP, Swisdak M, et al. (2012) The importance of plasma conditions for magnetic reconnection at Saturn’s magnetopause. Geophys Rev Lett 39(8):L08103. 10.1029/2012GL051372

[CR242] Matthaeus WH, Lamkin SL (1986) Turbulent magnetic reconnection. Phys Fluids 29:2513

[CR243] Mbarek R, Haggerty C, Sironi L, et al. (2022) Relativistic asymmetric magnetic reconnection. Phys Rev Lett 128(14):145101. 10.1103/PhysRevLett.128.145101. arXiv:2109.12125 [physics.plasm-ph] 35476472 10.1103/PhysRevLett.128.145101

[CR244] Melzani M, Walder R, Folini D, et al. (2014) Relativistic magnetic reconnection in collisionless ion-electron plasmas explored with particle-in-cell simulations. A & A 570:A111

[CR245] Mirnov VV, Hegna CC, Prager SC, et al. (2006) Two fluid dynamo and edge-resonant m = 0 tearing instability in reversed field pinch. In: IAEA FEC conf, China, p TH–P3–18

[CR246] Mitchell HG Jr, Kan JR (1978) Merging of magnetic fields with field-aligned plasma flow components. J Plasma Phys 20:31

[CR247] Mozer FS, Pritchett PL (2010) Magnetic field reconnection: a first-principles perspective. Phys Today 63(6):34. 10.1063/1.3455250

[CR248] Mozer FS, Bale SD, Phan TD (2002) Evidence of diffusion regions at a subsolar magnetopause crossing. Phys Rev Lett 89(1):015002. 10.1103/PhysRevLett.89.015002 12097047 10.1103/PhysRevLett.89.015002

[CR249] Müller D, St. Cyr OC, Zouganelis I, et al. (2020) The Solar Orbiter mission. Science overview. Astron Astrophys 642:A1. 10.1051/0004-6361/202038467. arXiv:2009.00861 [astro-ph.SR]

[CR250] Muñoz PA, Büchner J (2016) Non-Maxwellian electron distribution functions due to self-generated turbulence in collisionless guide-field reconnection. Phys Plasmas 23(10):102103. 10.1063/1.4963773. arXiv:1608.03110 [physics.plasm-ph]

[CR251] Murphy NA, Lukin VS (2015) Asymmetric magnetic reconnection in weakly ionized chromospheric plasmas. Astrophys J 805(2):134. 10.1088/0004-637X/805/2/134. arXiv:1504.01425 [astro-ph.SR]

[CR252] Murphy NA, Sovinec CR, Cassak PA (2010) Magnetic reconnection with asymmetry in the outflow direction. J Geophys Res 115:A09206

[CR253] Nagai T, Shinohara I, Fujimoto M, et al. (2001) Geotail observations of the Hall current system: evidence of magnetic reconnection in the magnetotail. J Geophys Res 106(A11):25929–25950. 10.1029/2001JA900038

[CR254] Nakamura M, Scholer M (2000) Structure of the magnetopause reconnection layer and of flux transfer events: ion kinetic effects. J Geophys Res 105:23179–23191

[CR255] Nakamura TKM, Nakamura R, Alexandrova A, et al. (2012) Hall magnetohydrodynamic effects for three-dimensional magnetic reconnection with finite width along the direction of the current. J Geophys Res 117:A03220

[CR256] Nakamura TKM, Eriksson S, Hasegawa H, et al. (2017a) Mass and energy transfer across the Earth’s magnetopause caused by vortex-induced reconnection. J Geophys Res Space Phys 122(11):11505–11522. 10.1002/2017JA024346

[CR257] Nakamura TKM, Haswgawa H, Daughton W, et al. (2017b) Turbulent mass transfer caused by vortex induced reconnection in collisionless magnetospheric plasmas. Nat Commun 8:1582 29150662 10.1038/s41467-017-01579-0 PMC5693928

[CR258] Nakamura TKM, Genestreti KJ, Liu YH, et al. (2018) Measurement of the magnetic reconnection rate in the Earth’s magnetotail. J Geophys Res Space Phys 123(11):9150–9168. 10.1029/2018JA025713

[CR259] Nakamura R, Genestreti KJ, Nakamura T, et al. (2019) Structure of the current sheet in the 11 July 2017 electron diffusion region event. J Geophys Res Space Phys 124(2):1173–1186. 10.1029/2018JA026028 31008008 10.1029/2018JA026028 PMC6472497

[CR260] Nakamura TKM, Blasl KA, Hasegawa H, et al. (2022) Multi-scale evolution of Kelvin-Helmholtz waves at the Earth’s magnetopause during southward IMF periods. Phys Plasmas 29(1):012901. 10.1063/5.0067391

[CR261] Nakamura R, et al (2025) Outlook. Space Sci Rev 221 10.1007/s11214-025-01145-x PMC11821752 39958871

[CR262] Newcomb WA (1958) Motion of magnetic lines of force. Ann Phys 3(4):347–385. 10.1016/0003-4916(58)90024-1

[CR263] Ng J, Yoo J, Chen LJ, et al. (2023) 3D simulation of lower-hybrid drift waves in strong guide field asymmetric reconnection in laboratory experiments. Phys Plasmas 30(4):042101. 10.1063/5.0138278

[CR264] Ni L, Lukin VS, Murphy NA, et al. (2018) Magnetic reconnection in strongly magnetized regions of the low solar chromosphere. Astrophys J 852(2):95. 10.3847/1538-4357/aa9edb. arXiv:1712.00582 [astro-ph.SR]

[CR265] Ni L, Ji H, Murphy NA, et al. (2020) Magnetic reconnection in partially ionized plasmas. Proc R Soc Lond Ser A 476(2236):20190867. 10.1098/rspa.2019.0867. arXiv:2003.13233 [physics.plasm-ph] 10.1098/rspa.2019.0867 PMC7209142 32398944

[CR266] Norgren C, Graham DB, Khotyaintsev YV, et al. (2018) Electron reconnection in the magnetopause current layer. J Geophys Res Space Phys 123(11):9222–9238. 10.1029/2018JA025676

[CR267] Norgren C, Chen LJ, Graham DB, et al (2025) Electron and ion dynamics in reconnection diffusion regions. Space Sci Rev 221

[CR268] Øieroset M, Phan TD, Fujimoto M, et al. (2001) In situ detection of collisionless reconnection in the Earth’s magnetotail. Nature 412(6845):414–417. 10.1038/35086520 11473310 10.1038/35086520

[CR269] Oka M, Fujimoto M, Nakamura TKM, et al. (2008) Magnetic reconnection by a self-retreating X line. Phys Rev Lett 101(20):205004 19113348 10.1103/PhysRevLett.101.205004

[CR270] Oka M, Birn J, Egedal J, et al. (2023) Particle acceleration by magnetic reconnection in geospace. Space Sci Rev 219(8):75. 10.1007/s11214-023-01011-8. arXiv:2307.01376 [physics.space-ph] 37969745 10.1007/s11214-023-01011-8 PMC10630319

[CR271] Olson J, Egedal J, Greess S, et al. (2016) Experimental demonstration of the collisionless plasmoid instability below the ion kinetic scale during magnetic reconnection. Phys Rev Lett 116(25):255001. 10.1103/PhysRevLett.116.255001 27391729 10.1103/PhysRevLett.116.255001

[CR272] Ono Y, Morita A, Katsurai M, et al. (1993) Experimental investigation of three-dimensional magnetic reconnection by use of two colliding spheromaks. Phys Fluids B 5(10):3691–3701. 10.1063/1.860840

[CR273] Parashar TN, Matthaeus WH, Shay MA (2018) Dependence of kinetic plasma turbulence on plasma . Astrophys J Lett 864:L21

[CR274] Parker EN (1957) Sweet’s mechanism for merging magnetic fields in conducting fluids. J Geophys Res 62(4):509–520

[CR275] Parker EN (1963) The solar-flare phenomenon and the theory of reconnection and annihilation of magnetic fields. Astrophys J 8:177

[CR276] Parker EN (1973) The reconnection rate of magnetic fields. Astrophys J 180:247

[CR277] Paschmann G, Daly PW (1998) Analysis methods for multi-spacecraft data. ISSI scientific reports series SR-001, ESA/ISSI

[CR278] Paschmann G, Sonnerup BUÖ, Papamastorakis I,et al. (1979) Plasma acceleration at the Earth’s magnetopause: evidence for reconnection. Nature 282(5736):243–246

[CR279] Paschmann G, Schwartz SJ, Escoubet CP, et al. (2005) Outer magnetospheric boundaries: cluster results. 10.1007/1-4020-4582-4

[CR280] Paschmann G, Øieroset M, Phan T (2013) In-situ observations of reconnection in space. Space Sci Rev 178(2–4):385–417. 10.1007/s11214-012-9957-2

[CR281] Payne DS, Genestreti KJ, Germaschewski K, et al. (2020) Energy balance and time dependence of a magnetotail electron diffusion region. J Geophys Res 125:e2020JA028290

[CR282] Payne DS, Farrugia CJ, Torbert RB, et al. (2021) Origin and structure of electromagnetic generator regions at the edge of the electron diffusion region. Phys Plasmas 28:112901

[CR283] Peery S, Liu YH, Li X (2024) Conditions for relativistic magnetic reconnection under the presence of shear flow and guide field. Astrophys J 964:144

[CR284] Pegoraro F (2016) Covariant magnetic connection hypersurfaces. J Plasma Phys 82(2):555820201. 10.1017/S0022377816000325. arXiv:1603.03909 [physics.plasm-ph]

[CR285] Petschek HE (1964) Magnetic field annihilation. In: Proc. AAS-NASA symp. phys. solar flares, pp 425–439

[CR286] Pezzi O, Yang Y, Valentini F, et al. (2019) Energy conversion in turbulent weakly collisional plasmas: Eulerian hybrid Vlasov-Maxwell simulations. Phys Plasmas 26(7):072301. 10.1063/1.5100125. arXiv:1904.07715

[CR287] Phan TD, Gosling JT, Paschmann G, et al. (2010) The dependence of magnetic reconnection on plasma and magnetic shear: evidence from solar wind observations. Astrophys J Lett 719:L199–L203. 10.1088/2041-8205/719/L199

[CR288] Phan TD, Paschmann G, Gosling JT, et al. (2013a) The dependence of magnetic reconnection on plasma and magnetic shear: evidence from magnetopause observations. Geophys Res Lett 40(1):11–16. 10.1029/2012GL054528

[CR289] Phan T, Shay M, Gosling J, et al. (2013b) Electron bulk heating in magnetic reconnection at Earth’s magnetopause: dependence on the inflow Alfvén speed and magnetic shear. Geophys Res Lett 40(17):4475–4480

[CR290] Phan T, Drake J, Shay M, et al. (2014) Ion bulk heating in magnetic reconnection exhausts at Earth’s magnetopause: dependence on the inflow Alfvén speed and magnetic shear angle. Geophys Res Lett 41(20):7002–7010

[CR291] Phan TD, Eastwood JP, Shay MA, et al. (2018) Electron magnetic reconnection without ion coupling in Earth’s turbulent magnetosheath. Nature 557(7704):202–206. 10.1038/s41586-018-0091-5 29743689 10.1038/s41586-018-0091-5

[CR292] Philippov A, Uzdensky DA, Spitkovsky A, et al. (2019) Pulsar radio emission mechanism: radio nanoshots as a low-frequency afterglow of relativistic magnetic reconnection. Astrophys J Lett 876(1):L6. 10.3847/2041-8213/ab1590

[CR293] Pollock C, Moore T, Jacques A, et al. (2016) Fast plasma investigation for magnetospheric multiscale. Space Sci Rev 199(1–4):331–406. 10.1007/s11214-016-0245-4

[CR294] Pontin DI, Priest ER (2022) Magnetic reconnection: MHD theory and modelling. Living Rev Sol Phys 19(1):1. 10.1007/s41116-022-00032-9

[CR295] Price L, Swisdak M, Drake JF, et al. (2016) The effects of turbulence on three-dimensional magnetic reconnection at the magnetopause. Geophys Res Lett 43:6020

[CR296] Priest E, Forbes T (2000) Magnetic reconnection. Cambridge University Press, Cambridge

[CR297] Priest ER, Titov VS, Grundy RE, et al. (2000) Exact solutions for reconnective magnetic annihilation. Proc R Soc Lond Ser A 456:1821

[CR298] Priest ER, Hornig G, Pontin DI (2003) On the nature of three-dimensional magnetic reconnection. J Geophys Res 108(A7):1285. 10.1029/2002JA009812

[CR299] Pritchard KR, Burch JL, Fuselier SA, et al. (2019) Energy conversion and electron acceleration in the magnetopause reconnection diffusion region. Geophys Res Lett 46(10274):10274–10282. 10.1029/2019GL084636

[CR300] Pritchard KR, Burch JL, Fuselier SA, et al. (2023) Reconnection rates at the Earth’s magnetopause and in the magnetosheath. J Geophys Res Space Phys 128(9):e2023JA031475. 10.1029/2023JA031475

[CR301] Pritchett PL (2001) Geospace environment modeling (GEM) magnetic reconnection challenge: simulations with a full particle electromagnetic code. J Geophys Res 106:3783

[CR302] Pritchett PL (2005) The “Newton challenge”: kinetic aspects of forced magnetic reconnection. J Geophys Res 110:A10213. 10.1029/2005JA011228

[CR303] Pucci F, Velli M (2014) Reconnection of quasi-singular current sheets: the “ideal” tearing mode. Astrophys J Lett 780:L19

[CR304] Pyakurel PS, Shay MA, Phan TD, et al. (2019) Transition from ion-coupled to electron-only reconnection: basic physics and implications for plasma turbulence. Phys Plasmas 26:082307

[CR305] Pyakurel PS, Shay MA, Drake JF, et al. (2021) Faster form of electron magnetic reconnection with a finite length X-line. Phys Rev Lett 127(15):155101. 10.1103/PhysRevLett.127.155101 34677989 10.1103/PhysRevLett.127.155101

[CR306] Raab W, Branduardi-Raymont G, Wang C, et al. (2016) SMILE: a joint ESA/CAS mission to investigate the interaction between the solar wind and Earth’s magnetosphere. In: den Herder JWA, Takahashi T, Bautz M (eds) Space telescopes and instrumentation 2016: ultraviolet to gamma ray, p 990502. 10.1117/12.2231984

[CR307] Rager AC, Dorelli JC, Gershman DJ, et al. (2018) Electron crescent distributions as a manifestation of diamagnetic drift in an electron-scale current sheet: magnetospheric multiscale observations using new 7.5 ms fast plasma investigation moments. Geophys Res Lett 45(2):578–584. 10.1002/2017GL076260. arXiv:1706.08435 [physics.space-ph] 29576666 10.1002/2017GL076260 PMC5856066

[CR308] Raouafi NE, Matteini L, Squire J, et al. (2023a) Parker Solar Probe: four years of discoveries at solar cycle minimum. Space Sci Rev 219(1):8. 10.1007/s11214-023-00952-4. arXiv:2301.02727 [astro-ph.SR]

[CR309] Raouafi NE, Stenborg G, Seaton DB, et al. (2023b) Magnetic reconnection as the driver of the solar wind. Astrophys J 945(1):28. 10.3847/1538-4357/acaf6c. arXiv:2301.00903 [astro-ph.SR]

[CR310] Ren Y, Yamada M, et al. (2005) Experiment verification of the Hall effect during magnetic reconnection in a laboratory plasma. Phys Rev Lett 95:055003 16090886 10.1103/PhysRevLett.95.055003

[CR311] Retinò A, Sundkvist D, Vaivads A, et al. (2007) In situ evidence of magnetic reconnection in turbulent plasma. Nat Phys 3(4):236–238

[CR312] Ripperda B, Bacchini F, Philippov AA (2020) Magnetic reconnection and hot spot formation in black hole accretion disks. Astrophys J 900(2):100. 10.3847/1538-4357/ababab. arXiv:2003.04330 [astro-ph.HE]

[CR313] Rogers BN, Denton RE, Drake JF, et al. (2001) Role of dispersive waves in collisionless magnetic reconnection. Phys Rev Lett 87(19):195004 11690418 10.1103/PhysRevLett.87.195004

[CR314] Roytershteyn V, Daughton W, Karimabadi H, et al. (2012) Influence of the lower-hybrid drift instability on magnetic reconnection in asymmetric configurations. Phys Rev Lett 108:185001 22681084 10.1103/PhysRevLett.108.185001

[CR315] Sato T, Hayashi T (1979) Externally driven magnetic reconnection and a powerful magnetic energy converter. Phys Fluids 22:1189–1202

[CR316] Schekochihin AA, Cowley SC (2006) Turbulence, magnetic fields, and plasma physics in clusters of galaxies. Phys Plasmas 13(5):056501. 10.1063/1.2179053. arXiv:astro-ph/0601246

[CR317] Schindler K (1974) A theory of the substorm mechanism. J Geophys Res 79:2803

[CR318] Schmitz H, Grauer R (2006) Kinetic Vlasov simulations of collisionless magnetic reconnection. Phys Plasmas 13(9):092309. 10.1063/1.2347101. arXiv:physics/0608175 [physics.plasm-ph]

[CR319] Schoeffler KM, Drake JF, Swisdak M (2011) The effects of plasma beta and anisotropy instabilities on the dynamics of reconnecting magnetic fields in the heliosheath. Astrophys J 743:70. 10.1088/0004-637X/743/1/70

[CR320] Scholer M (1989) Undriven magnetic reconnection in an isolated current sheet. J Geophys Res 94(A7):8805–8812

[CR321] Schreier R, Swisdak M, Drake JF, et al. (2010) Three-dimensional simulations of the orientation and structure of reconnection X-lines. Phys Plasmas 17:110704. 10.1063/1.3494218

[CR322] Schwartz SJ, Kucharek H, Farrugia CJ, et al. (2021) Energy conversion within current sheets in the Earth’s quasi parallel magnetosheath. Geophys Res Lett 48(4):e91859. 10.1029/2020GL091859

[CR323] Scott BD, Hassam AB (1987) Analytical theory of nonlinear drift-tearing mode stability. Phys Fluids 30(1):90–101

[CR324] Servidio S, Matthaeus WH, Shay MA, et al. (2009) Magnetic reconnection in two-dimensional magnetohydrodynamic turbulence. Phys Rev Lett 102:115003 19392208 10.1103/PhysRevLett.102.115003

[CR325] Shay MA, Swisdak M (2004) Three-species collisionless reconnection: effect of on magnetotail reconnection. Phys Rev Lett 93(17):175001 15525083 10.1103/PhysRevLett.93.175001

[CR326] Shay MA, Drake JF, Rogers BN, et al. (1999) The scaling of collisionless, magnetic reconnection for large systems. Geophys Res Lett 26(14):2163–2166

[CR327] Shay MA, Drake JF, Swisdak M, et al. (2003) Inherently three dimensional magnetic reconnection: a mechanism for bursty bulk flows? Geophys Res Lett 30(6):1345. 10.1029/2002GL016267

[CR328] Shay MA, Drake JF, Swisdak M (2007) Two-scale structure of the electron dissipation region during collisionless magnetic reconnection. Phys Rev Lett 99:155002. 10.1103/PhysRevLett.99.155002 17995175 10.1103/PhysRevLett.99.155002

[CR329] Shay MA, Haggerty CC, Phan TD, et al. (2014) Electron heating during magnetic reconnection: a simulation scaling study. Phys Plasmas 21:122902

[CR330] Shay MA, Phan TD, Haggerty CC, et al. (2016) Kinetic signatures of the region surrounding the X line in asymmetric (magnetopause) reconnection. Geophys Res Lett 43(9):4145–4154. 10.1002/2016GL069034

[CR331] Shay M, Adhikari S, Beesho N, et al (2025) Simulation models for exploring magnetic reconnection. Space Sci Rev 221 10.1007/s11214-025-01210-5 PMC12420772 40937341

[CR332] Shepherd LS, Cassak PA (2012) Guide field dependence of 3-D X-line spreading during collisionless magnetic reconnection. J Geophys Res 117:A10101

[CR333] Shi P, Srivastav P, Barbhuiya MH, et al. (2022) Laboratory observations of electron heating and non-Maxwellian distributions at the kinetic scale during electron-only magnetic reconnection. Phys Rev Lett 128(2):025002. 10.1103/PhysRevLett.128.025002 35089758 10.1103/PhysRevLett.128.025002

[CR334] Shi P, Scime EE, Barbhuiya MH, et al. (2023) Using direct laboratory measurements of electron temperature anisotropy to identify the heating mechanism in electron-only guide field magnetic reconnection. Phys Rev Lett 131:155101. 10.1103/PhysRevLett.131.155101 37897764 10.1103/PhysRevLett.131.155101

[CR335] Shibata K, Tanuma S (2001) Plasmoid-induced-reconnection and fractal reconnection. Earth Planets Space 53:473

[CR336] Shibata K, Nakamura T, Matsumoto T, et al. (2007) Chromospheric anemone jets as evidence of ubiquitous reconnection. Science 318(5856):1591. 10.1126/science.1146708. arXiv:0810.3974 [astro-ph] 18063790 10.1126/science.1146708

[CR337] Shuster JR, Chen LJ, Hesse M, et al. (2015) Spatiotemporal evolution of electron characteristics in the electron diffusion region of magnetic reconnection: implications for acceleration and heating. Geophys Res Lett 42:2586

[CR338] Sitnov MI, Swisdak M, Divin AV (2009) Dipolarization fronts as a signature of transient reconnection in the magnetotail. J Geophys Res 114:A04202

[CR339] Sitnov MI, Merkin VG, Roytershteyn V, et al. (2018) Kinetic dissipation around a dipolarization front. Geophys Res Lett 45(10):4639–4647. 10.1029/2018GL077874

[CR340] Song L, Zhou M, Yi Y, et al. (2020) Force and energy balance of the dipolarization front. J Geophys Res Space Phys 125(9):e2020JA028278. 10.1029/2020JA028278

[CR341] Sonnerup BUÖ (1974) Magnetopause reconnection rate. J Geophys Res 79(10):1546–1549

[CR342] Sonnerup BUÖ (1979) Magnetic field reconnection. In: Lanzerotti LJ, Kennel CF, Parker EN (eds) Solar System plasma physics, vol 3. North-Holland, Amsterdam, p 46

[CR343] Spinnangr SF, Hesse M, Tenfjord P, et al. (2021) The micro-macro coupling of mass-loading in symmetric magnetic reconnection with cold ions. Geophys Res Lett 48(13):e2020GL090690

[CR344] Stanier A, Simakov AN, Chacoń L, et al. (2015a) Fast magnetic reconnection with strong guide fields. Phys Plasmas 22:010701

[CR345] Stanier A, Simakov AN, Chacoń L, et al. (2015b) Fluid vs. kinetic magnetic reconnection with strong guide fields. Phys Plasmas 22:101203

[CR346] Stanier A, Daughton W, Le A, et al. (2019) Influence of 3D plasmoid dynamics on the transition from collisional to kinetic reconnection. Phys Plasmas 26:072121

[CR347] Stanish S, MacTaggart D (2024) On turbulent magnetic reconnection: fast and slow mean steady states. arXiv:2409.07346 [physics.plasm-ph]

[CR348] Stawarz JE, Eastwood JP, Phan TD, et al. (2019) Properties of the turbulence associated with electron-only magnetic reconnection in Earth’s magnetosheath. Astrophys J Lett 877(2):L37. 10.3847/2041-8213/ab21c8

[CR349] Stawarz JE, Eastwood JP, Phan TD, et al. (2022) Turbulence-driven magnetic reconnection and the magnetic correlation length: observations from magnetospheric multiscale in Earth’s magnetosheath. Phys Plasmas 29(1):012302. 10.1063/5.0071106

[CR350] Stawarz JE, Muñoz PA, Bessho N, et al. (2024) The interplay between collisionless magnetic reconnection and turbulence. Space Sci Rev 220:90. 10.1007/s11214-024-01124-8 39605945 10.1007/s11214-024-01124-8 PMC11589065

[CR351] Stern DP (1966) The motion of magnetic field lines. Space Sci Rev 6:147–173. 10.1007/BF00222592

[CR352] Sturrock PA (1966) Model of the high-energy phase of solar flares. Nature 211:695

[CR353] Sullivan BP, Rogers BN (2008) The scaling of forced collisionless reconnection. Phys Plasmas 15(10):102106. 10.1063/1.2992136. arXiv:0906.0334 [physics.plasm-ph]

[CR354] Sundkvist D, Retinò A, Vaivads A, et al. (2007) Dissipation in turbulent plasma due to reconnection in thin current sheets. Phys Rev Lett 99(2):025004. 10.1103/PhysRevLett.99.025004 17678230 10.1103/PhysRevLett.99.025004

[CR355] Sweet PA (1958) The neutral point theory of solar flares. In: Lehnet B (ed) IAU symp. in electromagnetic phenomena in cosmical physics. Cambridge University Press, New York, p 123

[CR356] Sweetser TH, Broschart SB, Angelopoulos V, et al. (2011) ARTEMIS Mission design. Space Sci Rev 165(1–4):27–57. 10.1007/s11214-012-9869-1

[CR357] Swisdak M, Drake JF (2007) Orientaion of the reconnection x-line. Geophys Res Lett 34:L11106

[CR358] Swisdak M, Rogers BN, Drake JF, et al. (2003) Diamagnetic suppression of component magnetic reconnection at the magnetopause. J Geophys Res 108(A5):1218. 10.1029/2002JA009726

[CR359] Swisdak M, Liu Y-H, Drake JF (2008) Development of a turbulent outflow during electron-positron magnetic reconnection. Astrophys J 680(2):999–1008. 10.1086/588088

[CR360] Swisdak M, Opher M, Drake JF, et al. (2010) The vector direction of the interstellar magnetic field outside the heliosphere. Astrophys J 710(2):1769–1775. 10.1088/0004-637X/710/2/1769

[CR361] Swisdak M, Drake JF, Price L, et al. (2017) Localize and intense energy converson in the diffusion region of asymmetric magnetic reconnection. Geophys Res Lett 45:5260

[CR362] Tavani M, Bulgarelli A, Vittorini V, et al. (2011) Discovery of powerful gamma-ray flares from the Crab Nebula. Science 331(6018):736. 10.1126/science.1200083. arXiv:1101.2311 [astro-ph.HE] 21212318 10.1126/science.1200083

[CR363] TenBarge JM, Daughton W, Karimabadi H, et al. (2014) Collisionless reconnection in the large guide field regime: gyrokinetic versus particle-in-cell simulations. Phys Plasmas 21:020708

[CR364] Tenfjord P, Hesse M, Norgren C, et al. (2019) The impact of oxygen on the reconnection rate. Geophys Res Lett 46(12):6195–6203

[CR365] Tharp TD, Yamada M, Ji H, et al. (2013) Study of the effects of guide field on Hall reconnection. Phys Plasmas 20(5):055705. 10.1063/1.4805244

[CR366] Toledo-Redondo S, Vaivads A, André M, et al. (2015) Modification of the Hall physics in magnetic reconnection due to cold ions at the Earth’s magnetopause. Geophys Res Lett 42(15):6146–6154

[CR367] Toledo-Redondo S, André M, Khotyaintsev YV, et al. (2016) Cold ion demagnetization near the x-line of magnetic reconnection. Geophys Res Lett 43(13):6759–6767

[CR368] Toledo-Redondo S, Dargent J, Aunai N, et al. (2018) Perpendicular current reduction caused by cold ions of ionospheric origin in magnetic reconnection at the magnetopause: particle-in-cell simulations and spacecraft observations. Geophys Res Lett 45(19):10033–10042. 10.1029/2018GL079051

[CR369] Toledo-Redondo S, Hwang KJ, Escoubet CP, et al. (2021) Solar wind—magnetosphere coupling during radial interplanetary magnetic field conditions: simultaneous multi-point observations. J Geophys Res Space Phys 126(11):e2021JA029506

[CR370] Torbert RB, Burch JL, Giles BL, et al. (2016a) Estimates of terms in Ohm’s law during an encounter with an electron diffusion region. Geophys Res Lett 43(12):5918–5925. 10.1002/2016GL069553

[CR371] Torbert RB, Russell CT, Magnes W, et al. (2016b) The FIELDS instrument suite on MMS: scientific objectives, measurements, and data products. Space Sci Rev 199(1–4):105–135. 10.1007/s11214-014-0109-8

[CR372] Torbert RB, Burch JL, Phan TD, et al. (2018) Electron-scale dynamics of the diffusion region during symmetric magnetic reconnection in space. Science 362(6421):1391–1395. 10.1126/science.aat2998. arXiv:1809.06932 [physics.space-ph] 30442767 10.1126/science.aat2998

[CR373] Tóth G, Jia X, Markidis S, et al. (2016) Extended magnetohydrodynamics with embedded particle-in-cell simulation of Ganymede’s magnetosphere. J Geophys Res Space Phys 121(2):1273–1293. 10.1002/2015JA021997

[CR374] Trattner KJ, Mulcock JS, Petrinec SM, et al. (2007) Probing the boundary between antiparallel and component reconnection during souwthward interplanetary magneitc field conditions. J Geophys Res 112:A01201

[CR375] Typer E, Cattell C, Thaller S, et al. (2016) Partitioning of integrated energy fluxes in four tail reconnection events observed by cluster. J Geophys Res 121:11798

[CR376] Uzdensky DA (2011) Magnetic reconnection in extreme astrophysical environments. Space Sci Rev 160(1–4):45–71. 10.1007/s11214-011-9744-5. arXiv:1101.2472 [astro-ph.HE]

[CR377] Vasyliunas VM (1972) Nonuniqueness of magnetic field line motion. J Geophys Res 77:6271

[CR378] Vasyliunas VM (1975) Theoretical models of magnetic field line merging, 1. Rev Geophys Space Phys 13(1):303

[CR379] von Goeler S, Stodiek W, Sauthoff N (1974) Studies of internal disruptions and oscillations in tokamak discharges with soft-x-ray techniques. Phys Rev Lett 33:1201

[CR380] Vörös Z, Yordanova E, Varsani A, et al. (2017) MMS observation of magnetic reconnection in the turbulent magnetosheath. J Geophys Res Space Phys 122(11):11442–11467. 10.1002/2017JA024535

[CR381] Walsh BM, Kuntz KD, Busk S, et al. (2024) The Lunar Environment Heliophysics X-ray Imager (LEXI) Mission. Space Sci Rev 220:37. 10.1007/s11214-024-01063-4 38756703 10.1007/s11214-024-01063-4 PMC11093736

[CR382] Wang S, Chen LJ, Bessho N, et al. (2018) Energy conversion andpartition in the asymmetrc reconnection diffusion region. J Geophys Res 123:8185

[CR383] Wang S, Chen LJ, Bessho N, et al. (2019) Observational evidence of magnetic reconnection in the terrestrial bow shock transition region. Geophys Res Lett 46(2):562–570. 10.1029/2018GL080944. arXiv:1812.09337 [physics.space-ph]

[CR384] Wang Y, Bandyopadhyay R, Chhiber R, et al. (2021) Statistical survey of collisionless dissipation in the terrestrial magnetosheath. J Geophys Res Space Phys 126(6):e2020JA029000. 10.1029/2020JA029000

[CR385] Wilder FD, Ergun RE, Eriksson S, et al. (2017) Multipoint measurements of the electron jet of symmetric magnetic reconnection with a moderate guide field. Phys Rev Lett 118(26):265101. 10.1103/PhysRevLett.118.265101 28707935 10.1103/PhysRevLett.118.265101

[CR386] Wilder FD, Ergun RE, Burch JL, et al. (2018) The role of the parallel electric field in electron-scale dissipation at reconnecting currents inthe magnetosheath. J Geophys Res Space Phys 123(8):6533–6547. 10.1029/2018JA025529

[CR387] Wygant JR, Cattell CA, Lysak R, et al. (2005) Cluster observations of an intense normal component of the electric field at a thin reconnecting current sheet in the tail and its role in shock-like acceleration of the ion fluid into the separatrix region. J Geophys Res 110:A09206

[CR388] Wyper PF, Antiochos SK, DeVore CR (2017) A universal model for solar eruptions. Nature 544(7651):452–455. 10.1038/nature22050 28447632 10.1038/nature22050

[CR389] Yamada M (2022) Magnetic reconnection. A modern synthesis of theory, experiment, and observations. Princeton University Press. 10.1515/9780691232980

[CR390] Yamada M, Levinton FM, Pomphrey N, et al. (1994) Investigation of magnetic reconnection during a sawtooth crash in a high-temperature tokamak plasma. Phys Plasmas 1(10):3269–3276

[CR391] Yamada M, Ji H, Hsu S, et al. (1997) Study of driven magnetic reconnection in a laboratory plasma. Phys Plasmas 4(5):1936–1944. 10.1063/1.872336

[CR392] Yamada M, Kulsrud R, Ji H (2010) Magnetic reconnection. Rev Mod Phys 82(1):603

[CR393] Yamada M, Yoo J, Myers CE (2016) Understanding dynamics and energetics of magnetic reconnection in a laboratory plasma: review of recent progress on selected fronts. Phys Plasmas 23:055402

[CR394] Yamada M, Chen LJ, Yoo J, et al. (2018) The two-fluid dynamics and energetics of the asymmetric magnetic reconnection in laboratory and space plasmas. Nat Commun 9:5223 30523290 10.1038/s41467-018-07680-2 PMC6283883

[CR395] Yang Y, Matthaeus WH, Parashar TN, et al. (2017a) Energy transfer, pressure tensor, and heating of kinetic plasma. Phys Plasmas 24(7):072306. 10.1063/1.4990421. arXiv:1705.02054

[CR396] Yang Y, Matthaeus WH, Parashar TN, et al. (2017b) Energy transfer channels and turbulence cascade in Vlasov-Maxwell turbulence. Phys Rev E 95:061201. 10.1103/PhysRevE.95.061201 28709288 10.1103/PhysRevE.95.061201

[CR397] Yang Y, Wan M, Matthaeus WH, et al. (2019) Scale dependence of energy transfer in turbulent plasma. Mon Not R Astron Soc 482(4):4933–4940. 10.1093/mnras/sty2977

[CR398] Yang Y, Matthaeus WH, Roy S, et al. (2022) Pressure–strain interaction as the energy dissipation estimate in collisionless plasma. Astrophys J 929(2):142. 10.3847/1538-4357/ac5d3e

[CR399] Yoo J, Ji JY, Ambat MV, et al. (2020) Lower hybrid drift waves during guide field reconnection. Geophys Res Lett 47(21):e87192. 10.1029/2020GL087192

[CR400] Yoo J, Ng J, Ji H, et al. (2024) Anomalous resistivity and electron heating by lower hybrid drift waves during magnetic reconnection with a guide field. Phys Rev Lett 132(14):145101. 10.1103/PhysRevLett.132.145101 38640378 10.1103/PhysRevLett.132.145101

[CR401] Yordanova E, Vörös Z, Varsani A, et al. (2016) Electron scale structures and magnetic reconnection signatures in the turbulent magnetosheath. Geophys Res Lett 43(12):5969–5978. 10.1002/2016GL069191. arXiv:1706.04053 [physics.space-ph]

[CR402] Yordanova E, Vörös Z, Raptis S, et al. (2020) Current sheet statistics in the magnetosheath. Front Astron Space Sci 7:2. 10.3389/fspas.2020.00002

[CR403] Zenitani S, Nagai T (2016) Particle dynamics in the electron current layer in collisionless magnetic reconnection. Phys Plasmas 23(10):102102. 10.1063/1.4963008. arXiv:1605.07472 [astro-ph.SR]

[CR404] Zenitani S, Hesse M, Kimas A, et al. (2011a) New measure of the dissipation region in collisionless magnetic reconnection. Phys Rev Lett 106:195003 21668168 10.1103/PhysRevLett.106.195003

[CR405] Zenitani S, Hesse M, Klimas A, et al. (2011b) The inner structure of collisionless magnetic reconnection: the electron-frame dissipation measure and Hall fields. Phys Plasmas 18(12):122108. 10.1063/1.3662430. arXiv:1110.3103 [astro-ph.SR]

[CR406] Zhong ZH, Deng XH, Zhou M, et al. (2019) Energy conversion and dissipation at dipolarization fronts: a statistical overview. Geophys Res Lett 46(22):12693–12701. 10.1029/2019GL085409

[CR407] Zhou M, Man H, Yang Y, et al. (2021) Measurements of energy dissipation in the electron diffusion region. Geophys Res Lett 48(24):e2021GL096372. 10.1029/2021GL096372

[CR408] Zuccarello FP, Seaton DB, Mierla M, et al. (2014) Observational evidence of torus instability as trigger mechanism for coronal mass ejections: the 2011 August 4 filament eruption. Astrophys J 785(2):88. 10.1088/0004-637X/785/2/88. arXiv:1401.5936 [astro-ph.SR]

[CR409] Zweibel EG (1989) Magnetic reconnection in partially ionized gases. Astrophys J 340:550. 10.1086/167416

[CR410] Zweibel EG, Yamada M (2009) Magnetic reconnection in astrophysical and laboratory plasmas. Annu Rev Astron Astrophys 47:291–332. 10.1146/annurev-astro-082708-101726

[CR411] Zweibel EG, Lawrence E, Yoo J, et al. (2011) Magnetic reconnection in partially ionized plasmas. Phys Plasmas 18(11):111211. 10.1063/1.3656960

